# Synthesis, Structures, and Complexation with Phenolic Guests of Acridone‐Incorporated Arylene–Ethynylene Macrocyclic Compounds

**DOI:** 10.1002/asia.202201003

**Published:** 2022-12-03

**Authors:** Takashi Komori, Eiji Tsurumaki, Shinji Toyota

**Affiliations:** ^1^ Department of Chemistry School of Science Tokyo Institute of Technology 2-12-1 Ookayama, Meguro-ku 152-8551 Tokyo Japan

**Keywords:** Acridone, Macrocycles, Host-guest systems, Sonogashira coupling, Molecular structures

## Abstract

Acridone units were incorporated into the arylene–ethynylene structure as polar arene units. Cyclic trimers consisting of three acridone‐2,7‐diyl units and three 1,3‐phenylene units were synthesized by Sonogashira couplings via stepwise or direct route. X‐ray analysis revealed that the trimer had a nearly planar macrocyclic framework with a cavity surrounded by three carbonyl groups. In contrast, the corresponding tetramer had a nonplanar macrocyclic framework. ^1^H NMR measurements showed that the trimer formed a 1 : 1 complex as a macrocyclic host with dihydric phenol guests, and the association constants were determined to be ca. 1.0×10^3^ L mol^−1^ for hydroquinone or resorcinol guests in CDCl_3_ at 298 K. The calculated structures of these complexes by the DFT method supported the presence of two sets of OH⋅⋅⋅O=C hydrogen bonds between the host and guest molecules. The spectroscopic data of the cyclic trimers and tetramers are compared with those of reference acridone compounds.

## Graphical abstract

**Acridone units** were incorporated into π‐conjugated macrocyclic structures as polar aromatic units. Target compounds were synthesized by Sonogashira couplings. A macrocycle with three acridone units had a planar and rigid framework and formed complexes with dihydric phenol derivatives such as hydroquinone via hydrogen bonds. The host‐guest properties and the structures of the complexes were investigated by experimental and theoretical approaches.

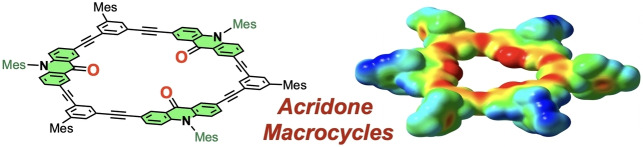

## Introduction

**Figure 1:**
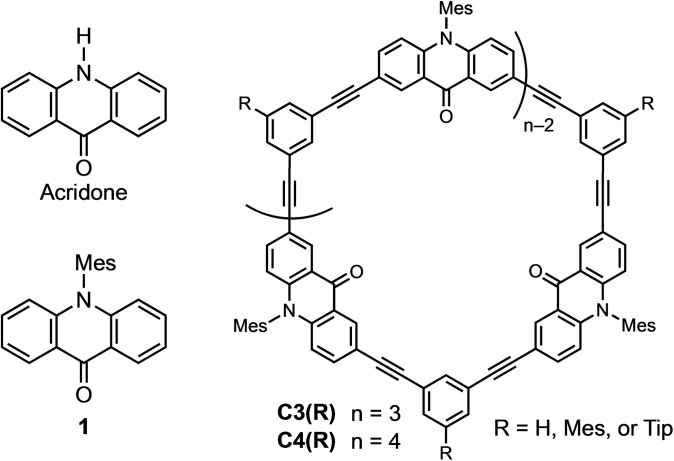
Structures of acridones and target macrocyclic compounds **C3** and **C4**. Mes: 2,4,6‐trimethylphenyl (mesityl), Tip: 2,4,6‐triisopropylphenyl.

Macrocyclic compounds based on aromatic units and acetylene linkers are versatile motifs for constructing various organic frameworks.^[1]^ The structures and properties of such compounds can be modified by changing the numbers and kinds of aromatic units. Cyclic oligomers consisting of six 1,3‐phenylene units and six acetylene linkers are typical examples of shape‐persistent hexagonal‐shaped compounds.^[2]^ Some of their derivatives possess characteristic electronic properties due to extended π‐conjugation and self‐assembly behavior due to π⋅⋅⋅π interactions.^[3]^ The size of the macrocyclic framework can be enlarged by incorporating large aromatic units that retain the geometrical requirement.^[4]^ Kobayashi et al. reported such a compound having three 2,7‐anthrylene units at alternating positions, which underwent self‐assembly in a solution or in the solid state.^[5]^ Recently, they synthesized an all 2,7‐anthrylene macrocycle, which included a [9]cycloparaphenylene molecule into the cavity.^[6]^ These compounds inspired us to incorporate other aromatic units to create novel macrocycles having various properties. We then adopted 9(10*H*)‐acridinone (acridone) units instead of anthracene units without changing the unit size, because the acridone structure has been applied in functional materials such as dyes, luminescent sensors, and electronic devices (Figure 1).^[7]^ It is remarkable that an acridone unit is highly polar with a large dipole moment (4.94 D) directing from the nitrogen side to the carbonyl side.^[8]^ In addition, the electron rich carbonyl‐oxygen atom can serve as a hydrogen bond acceptor or a Lewis base. Acridone units have been intelligently applied for the design of functional molecular cages for various guest species by Clever and the coworkers.^[9]^ Recently, we reported the synthesis of acyclic and cyclic acridone‐2,7‐diyl oligomers starting from 10‐mesitylacridone (**1**) by an iterative procedure.^[10]^ We herein designed macrocycles **C3**, where three acridone units were incorporated into the 1,3‐phenylene–ethynylene cyclic hexamer structure at alternating positions. Because the three carbonyl groups direct toward the macrocyclic center, the cavity should be surrounded by an electron‐rich region and interacting sites. Compounds having this framework are expected to be macrocyclic hosts as well as new types of π‐conjugated diethynylacridones. We herein report the synthesis, structures, and properties of cyclic trimers **C3** and tetramers **C4**, where we define oligomers as n‐mers depending on the number of acridone units. In particular, **C3(Mes)** having six mesityl (Mes) groups showed associative properties with phenolic guests such as hydroquinone via hydrogen bonds. The characteristic supramolecular behavior will be discussed on the basis of ^1^H NMR measurements and DFT calculations.

## Results and Discussion

### Synthesis and characterization

**Scheme 1:**
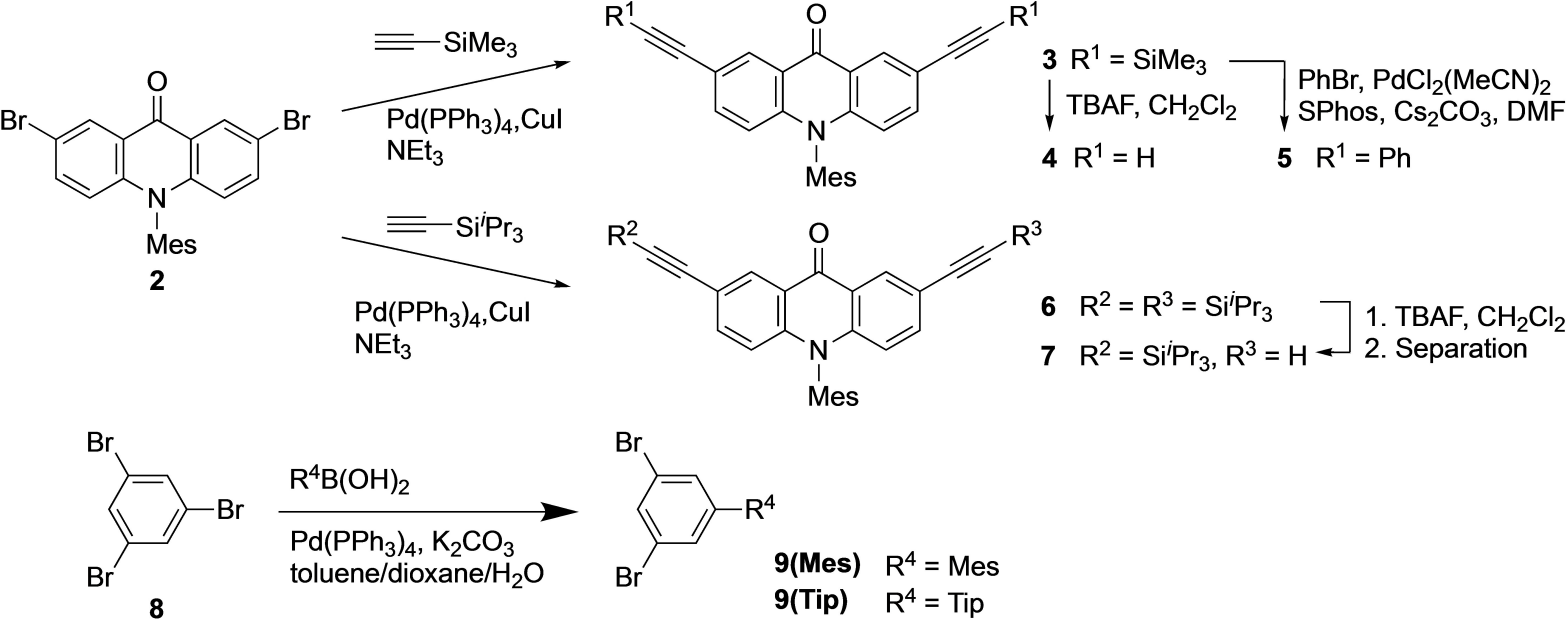
Preparation of 2,7‐diethynylacridone and 1,3‐phenylene units.

*Preparation of building units*. The target macrocycles were synthesized by the Sonogashira couplings from 2,7‐diethynylacridone derivatives and 1,3‐dihalobenzenes. Building units were prepared according to Scheme 1. We adopted 10‐mesitylacridone unit **1** to improve the solubility. The Sonogashira coupling of **2**, prepared by the known method,^[10]^ with (trimethylsilyl)ethyne afforded compound **3**, which was then desilylated with tetrabutylammonium fluoride (TBAF) to give terminal alkyne **4**. Reference compound **5** having two phenylethynyl groups was prepared by the Sonogashira coupling of **3** with bromobenzene. The singly silylated diethynylacridone unit was prepared by the Sonogashira coupling of **2** with (triisopropylsilyl)ethyne followed by desilylation of **6** with TBAF under a controlled condition, where the desired product **7** was separated from **4** and **6** by chromatography. Dibromobenzene derivatives **9(Mes)** ^[11]^ and **9(Tip)** were prepared by the Suzuki‐Miyaura coupling of 1,3,5‐tribromobenzene (**8**) and the corresponding boronic acids.

**Scheme 2:**
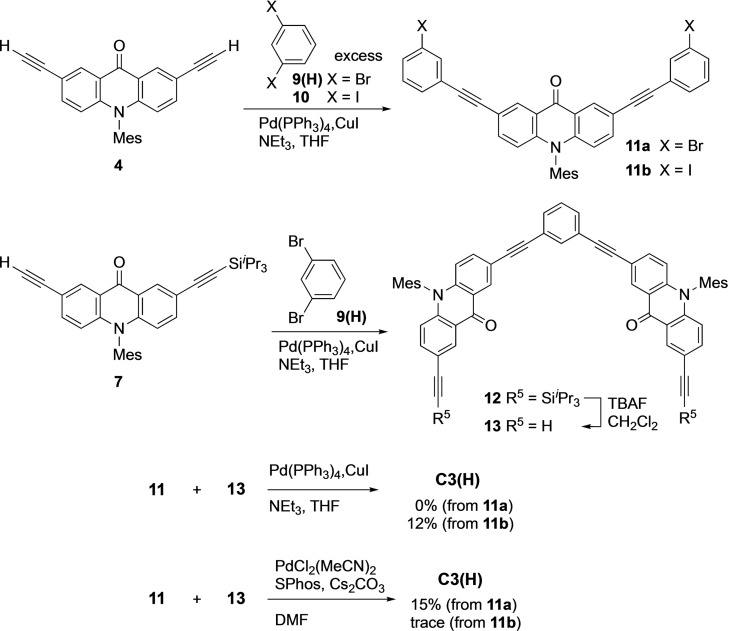
Synthesis of macrocycle **C3(H)** via stepwise routes.

*Synthesis via stepwise routes*. We first synthesized the target macrocycle in a stepwise manner following the reference approach (Scheme 2).^[5]^ Compounds **11 a** and **11 b** having two terminal 3‐halophenyl moieties were prepared by the Sonogashira coupling of **4** and an excess amount of the corresponding 1,3‐dihalobenzenes. Another unit **13** having two terminal alkynes was prepared by the Sonogahira coupling of **7** and 1,3‐dibromobenzene [**9(H)**] in a 2 : 1 ratio followed by the desilylation of **12**. Although the Sonogashira coupling of **11 a** and **13** was conducted under a standard condition with Pd(PPh_3_)_4_ and CuI, we could not find the formation of the desired cyclized product. When iodide **11 b** was used instead of bromide **11 a**, we were able to isolate **C3(H)** from the reaction mixture after repeated chromatographic separations as a yellow solid in 12% yield. The macrocyclization was also performed under the copper‐free condition reported by Gelman and Buchwald, which tended to suppress the undesired homocoupling reaction.^[12]^ The reaction of **11 a** and **13** with PdCl_2_(MeCN)_2_, SPhos, and Cs_2_CO_3_ in DMF afforded **C3(H)** in 15% isolated yield, whereas the reaction of **11 b** and **13** under the same conditions afforded a trace amount of the product.

**Scheme 3:**
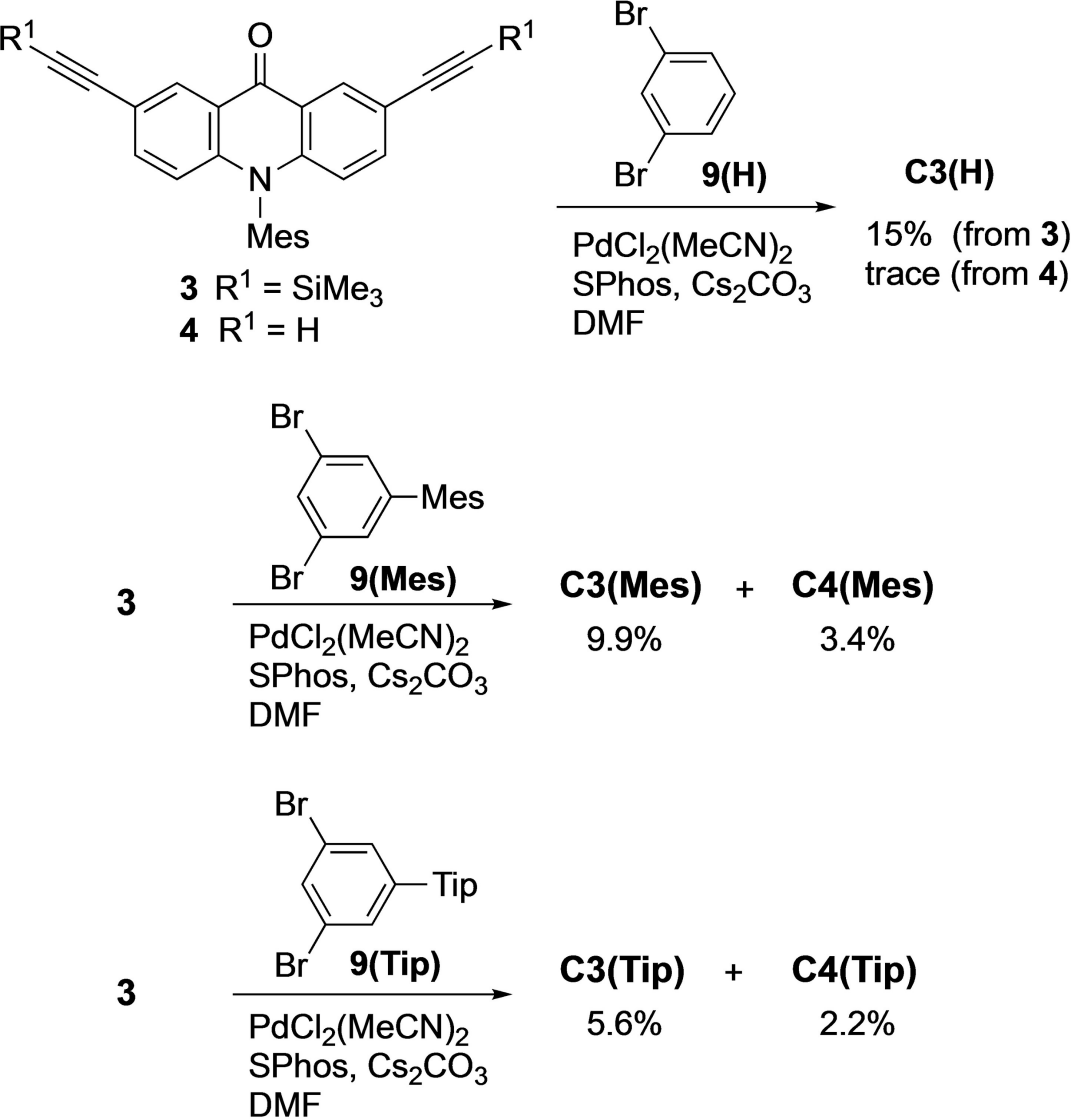
Synthesis of macrocycles **C3** and **C4** from monomeric units.

*Direct synthesis from monomeric units*. In order to shorten the overall steps, the six‐fold coupling directly from the monomeric units would be favorable over the stepwise route. Our early attempts to react **4** and **9(H)** under the standard Sonogashira coupling conditions were unsuccessful. We then applied the above copper‐free condition to the cyclization of **4** and **9(H)**, which gave a trace amount of the desired product (Scheme 3). However, the reaction of **3** and **9(H)** gave **C3(H)** in 15% isolated yield, where the desilylation and the coupling sequentially occurred under the reaction condition.^[13]^ Eventually, this direct synthesis was much more efficient than the stepwise synthesis mentioned above.

Because of the low solubility of **C3(H)**, we also synthesized derivatives with 5‐substituted 1,3‐phenylene units. The reaction of **3** and **9(Mes)** having a Mes group was carried out under the same condition. From the reaction mixture, we were able to isolate **C3(Mes)** and **C4(Mes)** in 9.9% and 3.4% yields, respectively, after separation by recycle gel permeation chromatography (GPC). Similarly, the reaction of **3** and **9(Tip)** afforded **C3(Tip)** and **C4(Tip)** in 5.6 and 2.2% yields, respectively.

*Characterization*. Macrocycle **C3(H)** was slightly soluble in CHCl_3_ and CH_2_Cl_2_. Its structure was characterized by the spectroscopic data and the X‐ray analysis. This compound showed a molecular ion peak at *m*/*z* 1305.5 in the mass spectrum, being consistent with the expected molecular weight C_96_H_63_N_3_O_3_. The ^1^H and ^13^C NMR spectra of **C3(H)** were simple, reflecting the formation of a cyclic structure of *D*_3h_ symmetry. In the ^1^H NMR spectrum, the signals due to the acridone moieties were observed as one set of ABX system, where the signal due to the 1,8‐H atoms was shifted downfield (8.90 ppm) because of the anisotropic effect of the adjacent carbonyl group. In the ^13^C NMR spectrum, the carbonyl and alkyne signals were observed at 177 ppm (one peak) and 89 ppm (two peaks), respectively. The IR spectrum of **C3(H)** showed an intense absorption at 1646 cm^−1^ due to the C=O stretching and a weak absorption at 2212 cm^−1^ due to the C≡C stretching (Figure S8).^[14]^ The other macrocycles were similarly characterized. For example, the mass spectra of **C3(Mes)** and **C4(Mes)** gave molecular ion peaks at *m*/*z* 1659.7 and 2213.0, respectively. As for the cyclic trimers, the solubility in ordinary organic solvents such as CHCl_3_ increased in the order of **C3(H)**, **C3(Mes)**, and **C3(Tip)**.

### Molecular structures

**Figure 2:**
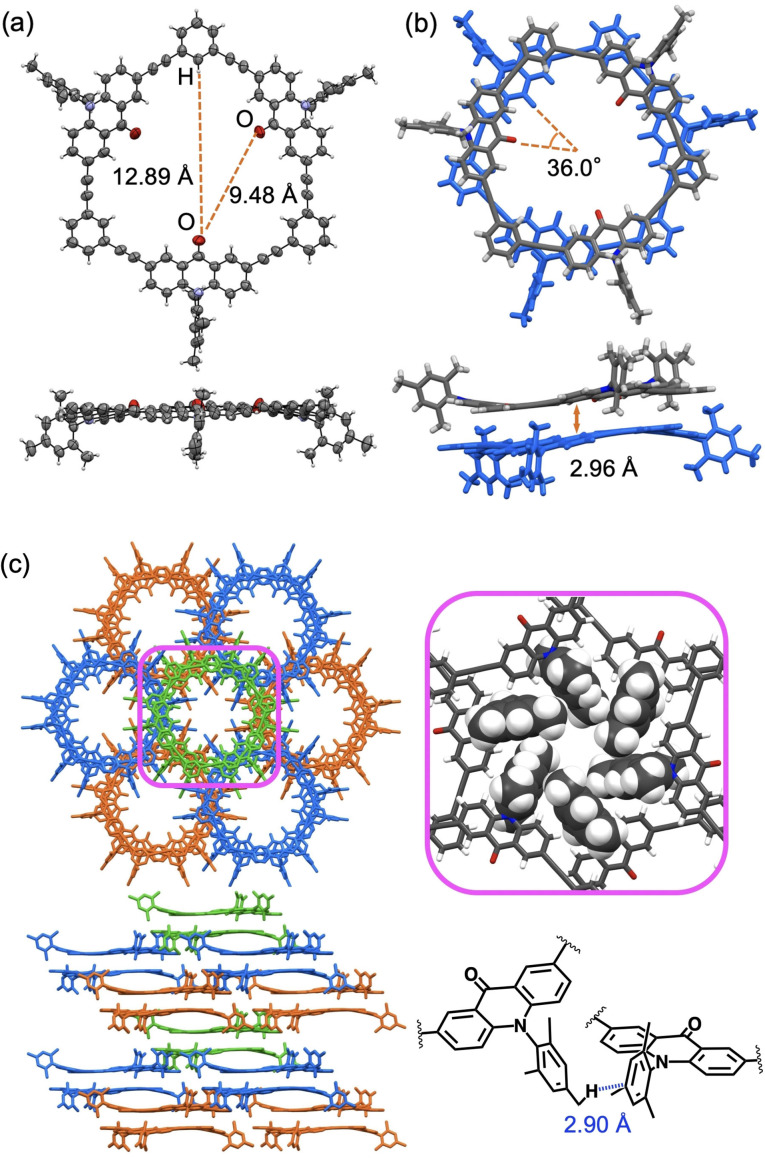
X‐ray structures of **C3(H)**. Solvent molecules are omitted for clarity. (a) ORTEP drawings of a single molecule. (b) Structures of a dimeric pair. (c) Packing diagrams. In an expanded diagram on the right, assembled mesityl groups are represented as a space‐filling model to highlight CH⋅⋅⋅π contacts.

We carried out X‐ray crystallographic analysis using a single crystal of **C3(H)** obtained from a 1,1,2,2‐tetrachloroethane/CH_3_CN solution. The structures of a single molecule and a dimeric stacked pair are shown in Figure 2. The molecule had a hexagonal‐like framework of nearly *D*_3h_ symmetry consisting of three acridone units, three 1,3‐phenylene units, and six acetylene linkers. The macrocyclic framework is slightly deformed from the planar structure, where the dihedral angles between the acridone and phenylene units across the acetylene linkers are ca. ±12°. The Mes groups take a nearly bisecting conformation relative to the acridone planes. A molecule has a triangular cavity, where the distance between the carbonyl‐oxygen atom and the hydrogen atom at the opposite side is 12.89 Å, and the distance between the oxygen atoms is 9.48 Å. The inner diameter of the cavity is estimated to be 7.9 Å assuming the van der Waals radius of oxygen atoms (1.52 Å). This size was smaller than the cavity size of acridone‐2,7‐diyl cyclic hexamer (ca. 9.1 Å).^[10]^

In the crystal, molecules form nearly fully stacked dimeric pairs at a separation of 2.96 Å (Figure 2b). The two molecules rotate by 36° each other along the axis passing through their centroids. This orientation should be preferable to maximize intermolecular π–π interactions and minimize the steric hindrance between the bulky Mes groups. In the crystal packing, dimeric pairs form a layer spreading along the *a*‐*b* plane in a hexagonal manner, and each layer stacks along the *c* axis in a sequence similar to the cubic closest packing structure (Figure 2c). Each cavity surrounded by host molecules is rather closed for **C3(H)**. This type of packing was occasionally observed for disk‐type molecules, for example, a 1,3‐phenylene–ethynylene cyclic hexamer with phenolic hydroxyl groups, and a similar pyridine‐containing macrocycle.[^2b^, ^15^] Interestingly, six peripheral mesityl groups each from different macrocyclic molecules form a cyclic network via CH⋅⋅⋅π interactions between the *p*‐Me groups and the benzene moieties (ca. 2.9 Å) as shown in Figure 2c. The X‐ray analysis revealed that **C3(Tip)** also formed dimeric pairs and a layer type packing in the crystal (Figure S9). In contrast to **C3(H)**, each layer stacks in a sequence similar to the hexagonal closest packing structure to form one dimensional channels.

**Figure 3:**
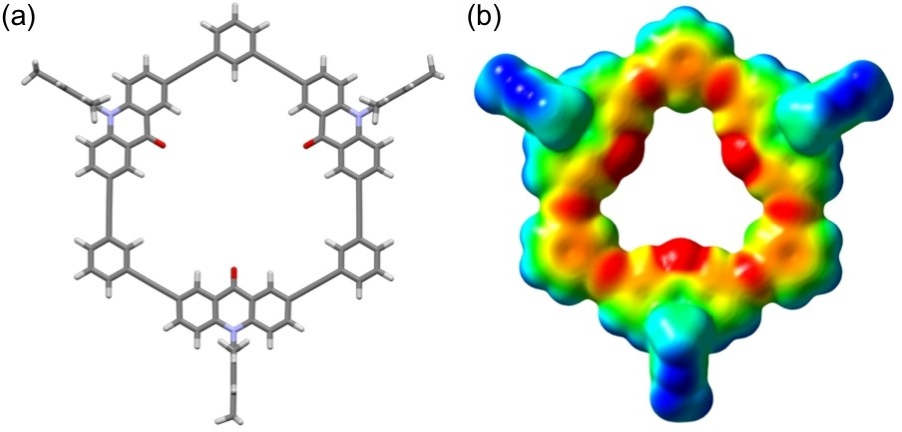
(a) Calculated structure of **C3(H)** at B3LYP/6‐31G(d) level and (b) its ESP map. Isodensity surface color: red (−0.03) to blue (+0.02).

The molecular structure of **C3(H)** was optimized by the DFT method at B3LYP/6‐31G(d) level (Figure 3a). In contrast to the X‐ray structure, the macrocyclic framework was completely planar. This difference means that the out‐of‐plane deformation in the observed structure is attributed to the packing effect. The electrostatic potential (ESP) map was visualized for the calculated structure of **C3(H)** as shown in Figure 3b.^[16]^ The electron‐rich red surface is located around the electronegative oxygen atoms and the acetylene moieties, resulting in a cavity surrounded by an electron‐rich wall. In contrast, the electron deficient blue surface spreads over the peripheral region involving the nitrogen atoms. The calculated dipole moment was nearly zero (*μ* 0.077 D) because of the formation of the symmetric cyclic structure.

**Figure 4:**
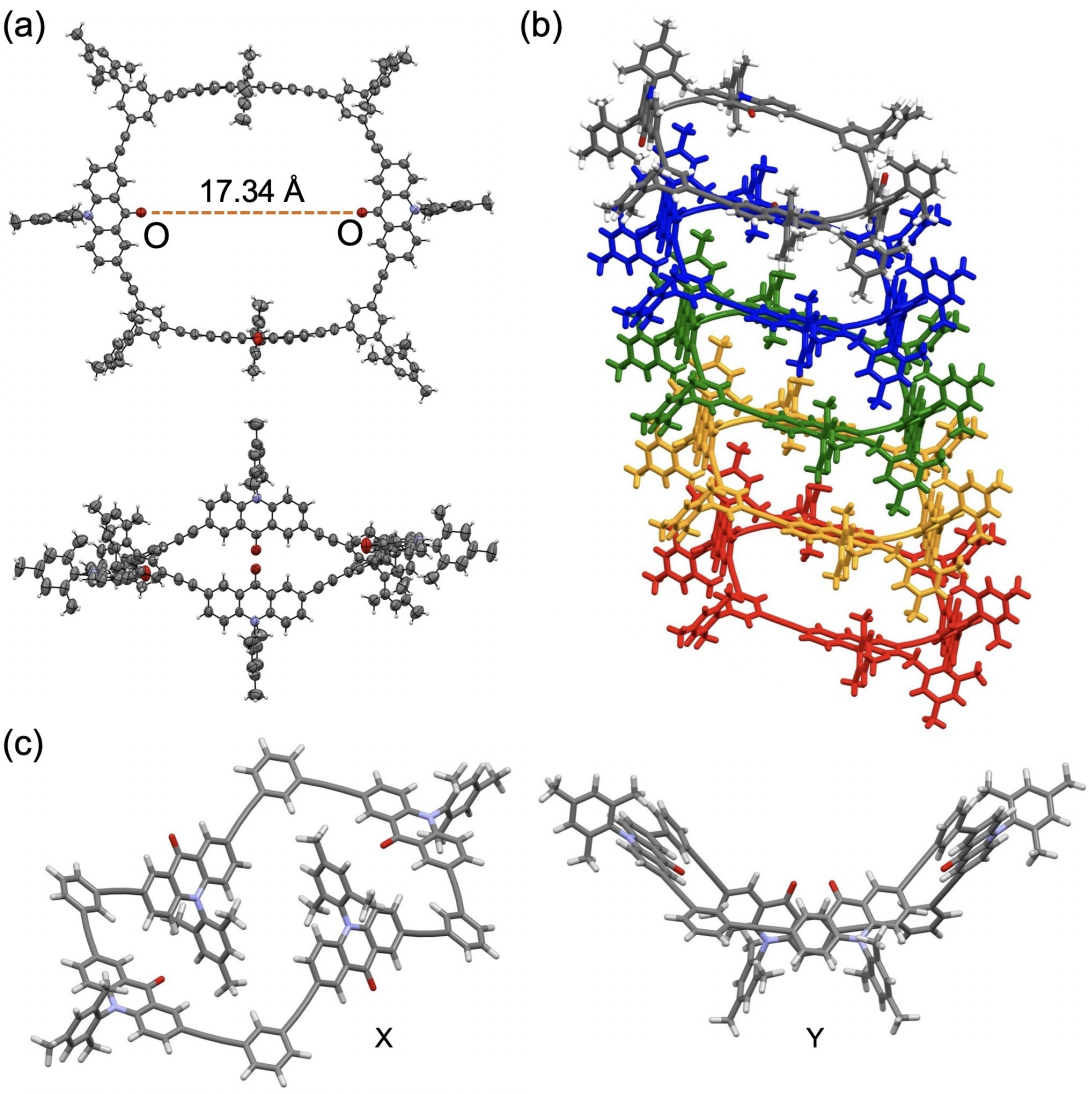
Molecular structures of **C4**. (a) X‐ray structures of a single molecule of **C4(Mes)**. (b) Packing diagram of the X‐ray structure of **C4(Mes)**. (c) Two energy‐minimum structures, X and Y, of **C4(H)** calculated at B3LYP/6‐31G(d) level.

We were able to obtain a single crystal of **C4(Mes)** from a THF solution as well. The X‐ray structures are shown in Figure 4. The macrocyclic framework takes a nonplanar conformation of nearly *C*_2h_ symmetry. Relative to the averaged macrocyclic plane, two acridone units at the opposite sides are nearly coplanar, and the other two units are nearly perpendicular toward the opposite directions. As a result, the dihedral angles between the acridone and phenylene units across four acetylene linkers are nearly 90°. This conformation is similar to a chair‐like conformation of 1,3‐phenylene–ethynylene cyclic octamer.^[17]^ The distance between the carbonyl‐oxygen atoms in the coplanar acridone units is 17.34 Å, resulting in a large cavity of ca. 14 Å diameter along this direction. The bond angles at the acetylene carbons are in the range of 171.1–178.5°, indicating small bending deformations at some sp carbons. In the crystal, molecules form a columnar type stacking at a separation of 11.5 Å along the *a* axis to form cavity channels along the stacking direction (Figure 4b). The structural optimization of **C4(H)** at the B3LYP/6‐31G(d) level afforded two energy‐minimum structures, chair‐like conformation X and boat‐like conformation Y, where the former was less stable by 1.2 kJ mol^−1^ than the latter (Figure 4c). The macrocyclic framework in the X‐ray structure of **C4(Mes**) is similar to the chair‐like conformation X of **C4(H)**. The small energy difference suggests that the two conformers can exist in a comparable ratio in a solution. These conformers should readily exchange each other via rotation about the acetylene axes.^[18]^

### UV‐vis and fluorescence spectra

**Figure 5:**
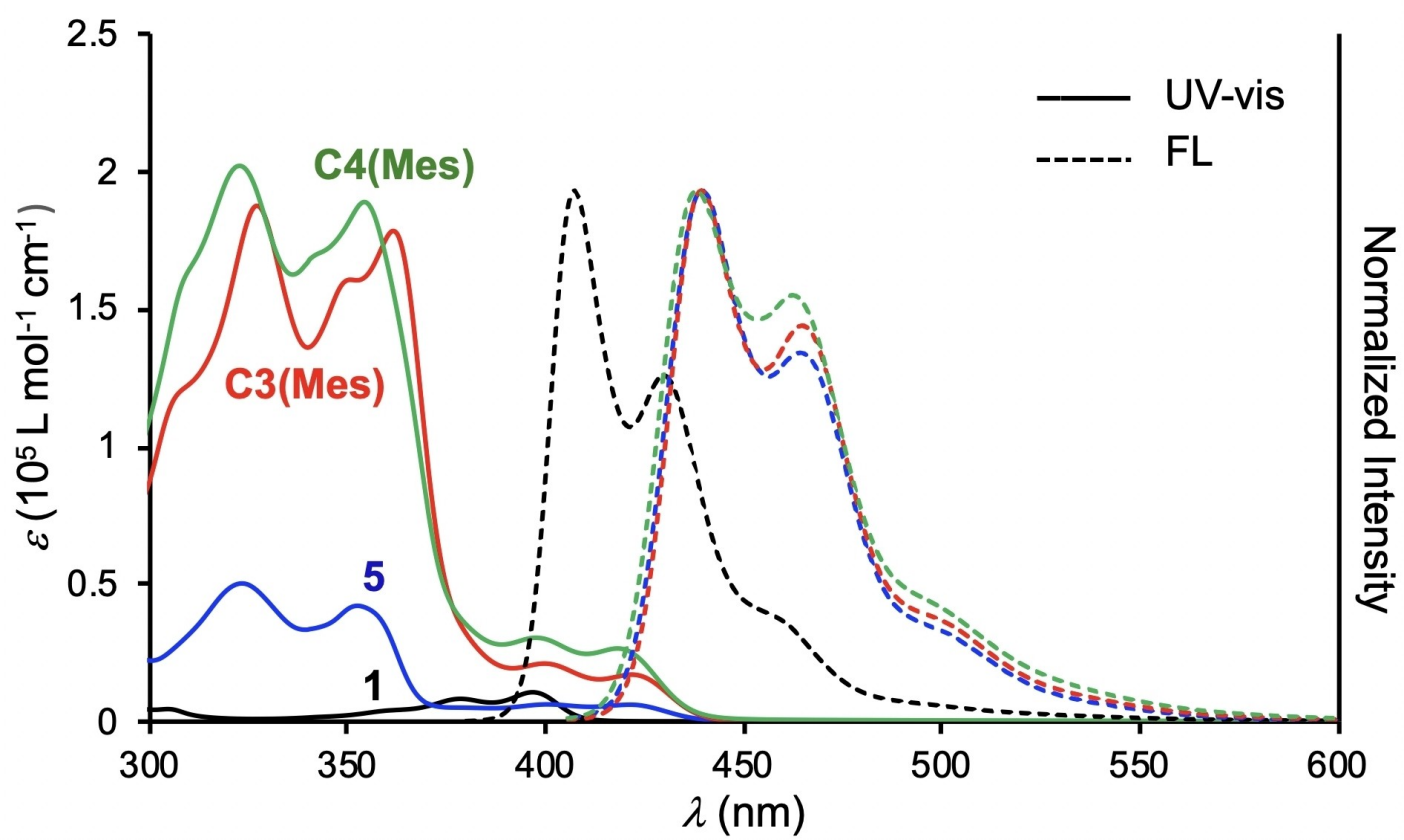
UV‐vis and fluorescence spectra of **C3(Mes)**, **C4(Mes)**, and related compounds measured in CHCl_3_ at room temperature. Concentration 1.0×10^−5^ mol L^−1^.

**Figure 6:**
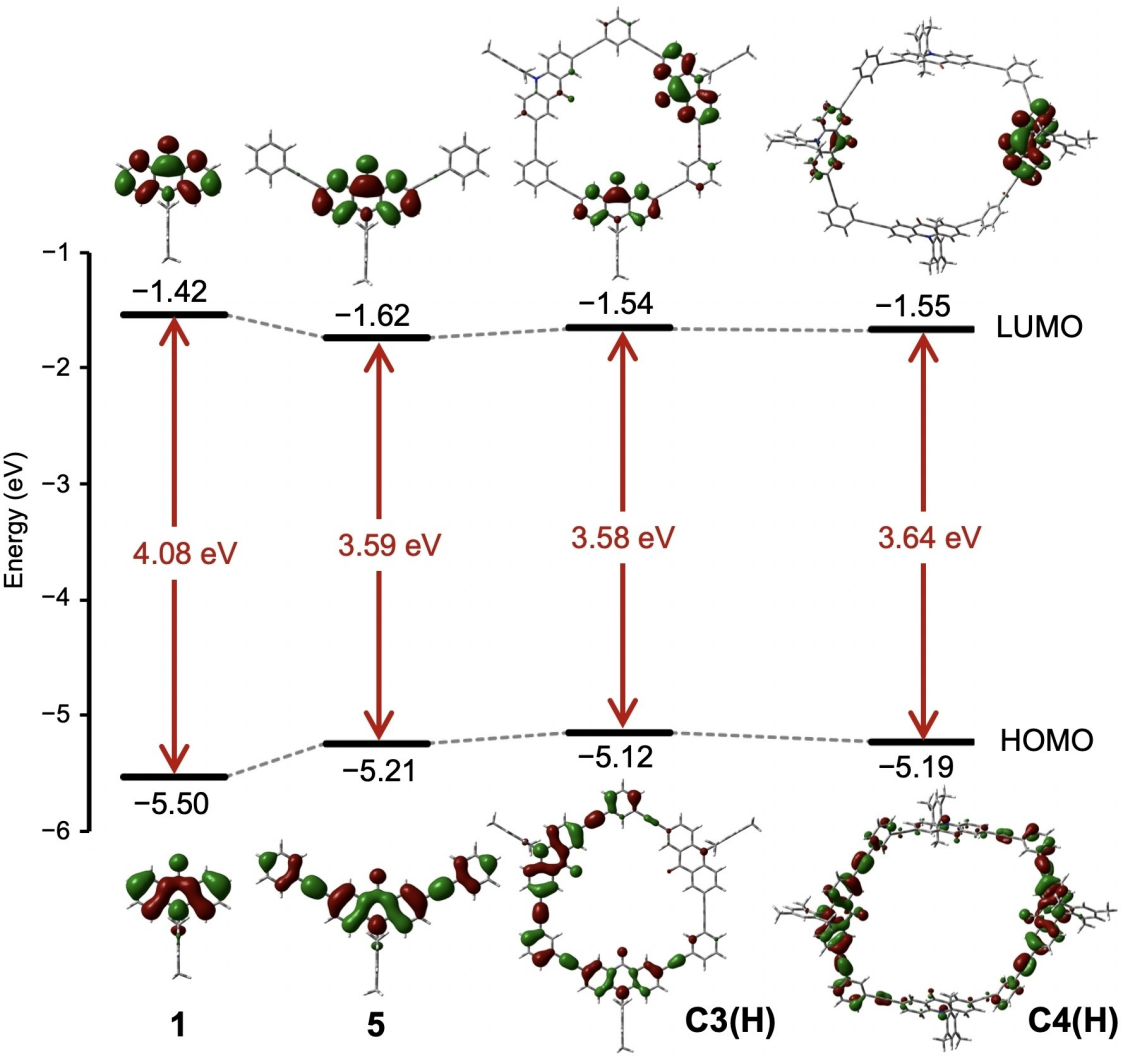
Frontier Kohn‐Sham orbitals and orbital energy diagrams of **1**, **5**, **C3(H)**, and **C4(H)** at the B3LYP/6‐31G(d) level.

UV‐vis absorption and fluorescence spectra of the macrocycles and the related compounds were measured in CHCl_3_ (Table 1). The spectra of **C3(Mes)** and **C4(Mes)** are compared with those of 10‐mesitylacridone (**1**) and 2,7‐bis(phenylethynyl)acridone (**5**) as reference compounds in Figure 5. In the UV‐vis spectra, compound **C3(Mes)** showed intense peaks at 300–380 nm and weak structured bands at 380–430 nm. The latter bands are assigned to the HOMO–LUMO excitation accompanying intramolecular charge transfer from the electron rich phenylethynyl moieties to the electron deficient acridone moieties according to the time‐dependent (TD)‐DFT calculation of **C3(H)** (Figures S10 and S11), as observed for other 2,7‐bis(phenylethynyl)acridone derivatives.^[19]^ The absorption maximum at the longest wavelength was observed at 422 nm, which was identical to that of **5** and red‐shifted by 20 nm relative to that of **1** (397 nm). The absorption bands of **C4(Mes)** slightly blue‐shifted relative to those of **C3(Mes)**. These tendencies are consistent with the calculated HOMO–LUMO gap energies of **1**, **5**, **C3(H)**, and **C4(H)**, as shown in Table 1 and Figure 6.

**Table 1:** UV‐vis and fluorescence spectral data of **C3**, **C4**, and related compounds measured in CHCl_3_ at room temperature.

	UV‐vis^[a]^	FL^[a]^			Stokes shift	*ΔE*_HOMO‐LUMO_
	*λ*_max_ [nm] (*ϵ* [L mol^−1^ cm^−1^])	*λ*_em_ [nm]	*Φ*_f_^[b]^	*τ*_f_ [ns]^[c]^	[cm^−1^]	[eV]^[d]^
**C3(H)**	421 (20600)	439	0.084	2.0	970	3.58
**C4(H)**	–	–	–	–	–	3.64
**C3(Mes)**	422 (16800)	439	0.091	2.1	920	3.58
**C4(Mes)**	418 (26500)	438	0.093	1.9 (0.74), 2.8 (0.26)	1100	–
**C3(Tip)**	422 (19300)	439	0.081	2.1	920	–
**C4(Tip)**	419 (26200)	438	0.092	2.1	1000	–
**1**	397 (10500)	405	0.39	5.7	380	4.08
**5**	422 (6190)	440	0.10	2.0	970	3.59

[a] Concentration at 1.0×10^−5^ mol L^−1^. [b] Absolute fluorescence quantum yield. [c] Fluorescence lifetime. Values in parentheses are ratios of emission components. [d] Calculated at B3LYP/6‐31G(d) level.

In the fluorescence spectra, the emission bands of **C3(Mes)** and **C4(Mes)** were observed at ca. 440 nm, which was comparable to that of **5** and red‐shifted by 34 nm relative to that of **1**. Compounds **C3(Mes)**, **C4(Mes)**, and **5** had similar values of the fluorescence quantum yields *Φ*_f_ (ca. 0.1), the fluorescence lifetimes *τ*_f_ (2.0 ns), and the Stokes shifts (ca. 1000 cm^−1^). The relatively small Stokes shifts indicate small structural changes at the excited state.^[20]^ The spectroscopic properties of other cyclic oligomers, **C3(H)**, **C3(Tip)**, and **C4(Tip)**, are similar to those of **C3(Mes)** and **C4(Mes)**. These data indicate that a 2,7‐bis(phenylethynyl)acridone substructure plays an important role in determining the photophysical properties of the macrocycles.

The UV‐vis and fluorescence spectra of soluble derivative **C3(Tip)** were measured in several organic solvents (Figures S5 and S6 and Table S1). The absorption band at the longest wavelength slightly red‐shifted with increasing the polarity from THF (392 nm) to CH_3_OH (399 nm). The emission band also showed a red‐shift from THF (398 nm) to CH_3_OH (415 nm). Even though the overall solvent effect was small, a significant red‐shift was observed in CH_3_OH followed by CHCl_3_ and DMSO. The solvent effect of CHCl_3_ tends to be large compared with that expected from the solvent polarity as scaled by *E*_T_(30).^[21]^ This fact indicates that not only the solvent polarity but also the acceptor property, which can be scaled, for example, by acceptor number *AN*,^[22]^ influences the overall solvent effect on the absorption and emission bands (Figure S7). It is understandable that solvents having large acceptor characters should interact with the carbonyl‐oxygen atoms regardless of their polarities.

### DFT calculations

The molecular orbitals of **C3(H)** and **C4(H)** were calculated by the DFT method at the B3LYP/6‐31G(d) level. The energies of the HOMO and LUMO levels are shown in Figure 6. The HOMO‐LUMO gap of **C3(H)** (3.58 eV) is comparable to that of **5** (3.59 eV), and these values are smaller by ca. 0.5 eV than that of **1**. For **C3(H)**, the orbitals spread over two acridone units and attached phenylethynyl moieties at the HOMO level, whereas the orbitals are located on two acridone units at the LUMO level. The HOMO‐LUMO gap of **C4(H)** (3.64 eV) is larger than that of **C3(H)** due to the stabilization of the HOMO level. This finding is attributed to the nonplanar conformation of **C4(H)**, which prevents the π‐conjugation across aromatic units. The small blue‐shift in the UV‐vis spectrum of **C4(Mes)** relative to **C3(Mes)** can be attributed to this difference.

### Supramolecular chemistry

The ^1^H NMR spectra of **C3(Tip)** were measured at variable concentrations in CDCl_3_. The chemical shifts were almost unaffected by the concentration in the range of 1.0×10^−4^–2.0×10^−2^ mol L^−1^. This observation means that self‐association should be negligibly weak in the solution, in contrast to other disk‐type macrocycles.[^2^, ^3^, ^4^, ^5^]

We then investigated the association of **C3(Mes)** with external guest molecules. The cavity of **C3(Mes)** is surrounded by the electron‐rich oxygen atoms, which can interact with electron‐deficient moiety. In fact, acridone is known to be a good hydrogen bond acceptor according to the p*K*_HB_ parameter.^[23]^ For these reasons, we selected phenol derivatives as guest candidates.^[24]^ For the preliminary screening, we measured the ^1^H NMR spectra of **C3(Mes)** in the presence of a large excess (10 eq.) of phenol derivatives in CDCl_3_ (Figure S16). As a result, we observed small downfield shifts of the signals due to the aromatic protons directing inward (1,8‐H and 2’‐H) when resorcinol (**RE**), hydroquinone (**HQ**), and pyrogallol were added. On the other hand, changes were very small for phenol (**PH**), 1‐naphthol, catechol (**CA**), and other phenols. Therefore, we chose **RE** and **HQ** as promising guest candidates, and their behavior was compared with that of **CA** and **PH**.

**Figure 7:**
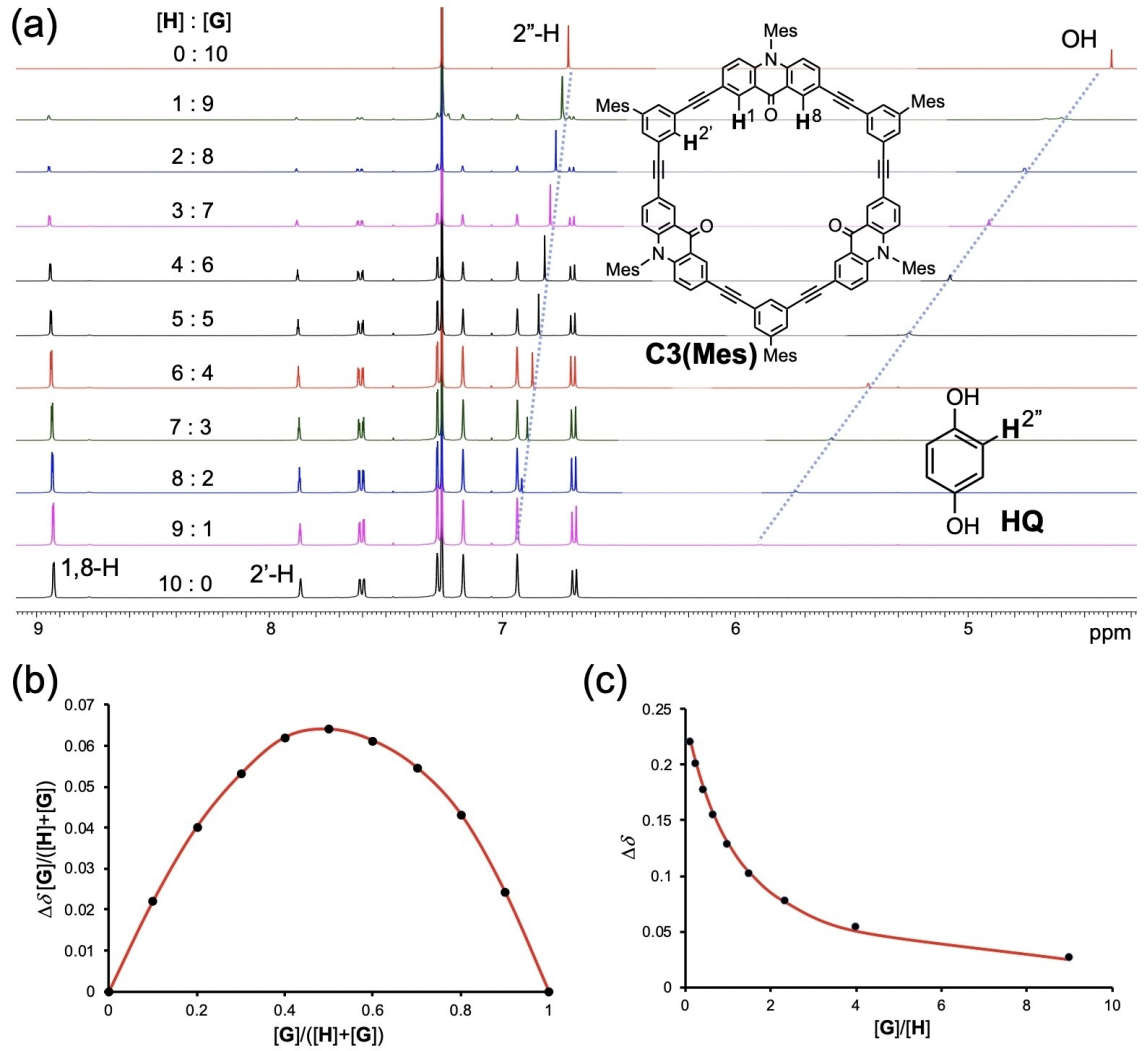
(a) ^1^H NMR spectra of mixtures of **C3(Mes)** (host: **H**) and hydroquinone **HQ** (guest: **G**) at various ratios in CDCl_3_ at 298 K. [**H**]+[**G**]=1.0×10^3^ mol L^−1^. (b) Job's plot. (c) Plot of chemical shift changes *Δδ* of **HQ** vs [**G**]/[**H**] ratio. The aromatic signal of **HQ** (2”‐H) was analyzed for (b) and (c).

We measured the ^1^H NMR spectra of mixtures of **C3(Mes)** and **HQ** at various ratios in CDCl_3_, where the sum of the host and guest concentrations was fixed at 1.0×10^−3^ mol L^−1^ (Figure 7a). As the host/guest ratio increased, the signals of **HQ** significantly shifted downfield (Δ*δ* 1.51 ppm for OH and Δ*δ* 0.22 ppm for 2”‐H). The large shielding of the phenolic protons supports the formation of hydrogen bonds. Because the chemical shift of the OH signal would be sensitive to various factors,^[25]^ that of the aromatic signal was used for the quantitative analysis (Figure 7bc). The Job's plot showing a maximum at 0.5 molar ratio suggests the formation of a 1 : 1 host‐guest complex. The least‐square fitting assuming the following equilibrium, **H**+**G** $\overset{\to }{\leftarrow }$ **H ⋅ G**, afforded the association constant *K*_a_ to be (1.00±0.09)×10^3^ L mol^−1^, corresponding to 17.1 kJ mol^−1^ in −Δ*G*_298_. This phenomenon was specific for **C3(Mes)**, and no significant changes in the chemical shifts were observed for **C4(Mes)**, **1**, or **5** upon addition of **HQ**.

We performed similar measurements with **C3(Mes)** and **RE** (Figure S17). The observed data supported the formation of a 1 : 1 complex, and the association constant *K*_a_ was (0.99±0.14)×10^3^ L mol^−1^ by monitoring the chemical shift of the 4’’,6’’‐H aromatic signal of **RE**.^[26]^ The analyses of other aromatic signals (2’’‐H or 5’’‐H) gave comparable association constants, confirming that all the probes give information of the identical process. Although the screening experiment had been almost negative, the measurements with **CA** showed small chemical shift changes by monitoring the chemical shift of the 3’’,6’’‐H aromatic signal of **CA** (Figure S18). The association constant *K*_a_ was estimated to be (2.3±0.3)×10^2^ L mol^−1^ according to the data measured at the total host and guest concentration of 2.0×10^−3^ mol L^−1^. No changes were observed during the measurements upon addition of **PH** (Figure S19).

The above data mean that macrocycle **C3(Mes)** associates with **HQ** and **RE** with a comparable strength, weakly with **CA**, and negligibly weakly with **PH**. This tendency means that multipoint interactions between the two OH groups in the guest and the carbonyl‐oxygen atoms in the macrocyclic host are important in the association behavior. In addition, the association energies depend on the position, namely distance and direction, of the two OH groups in the guest molecules.

**Figure 8:**
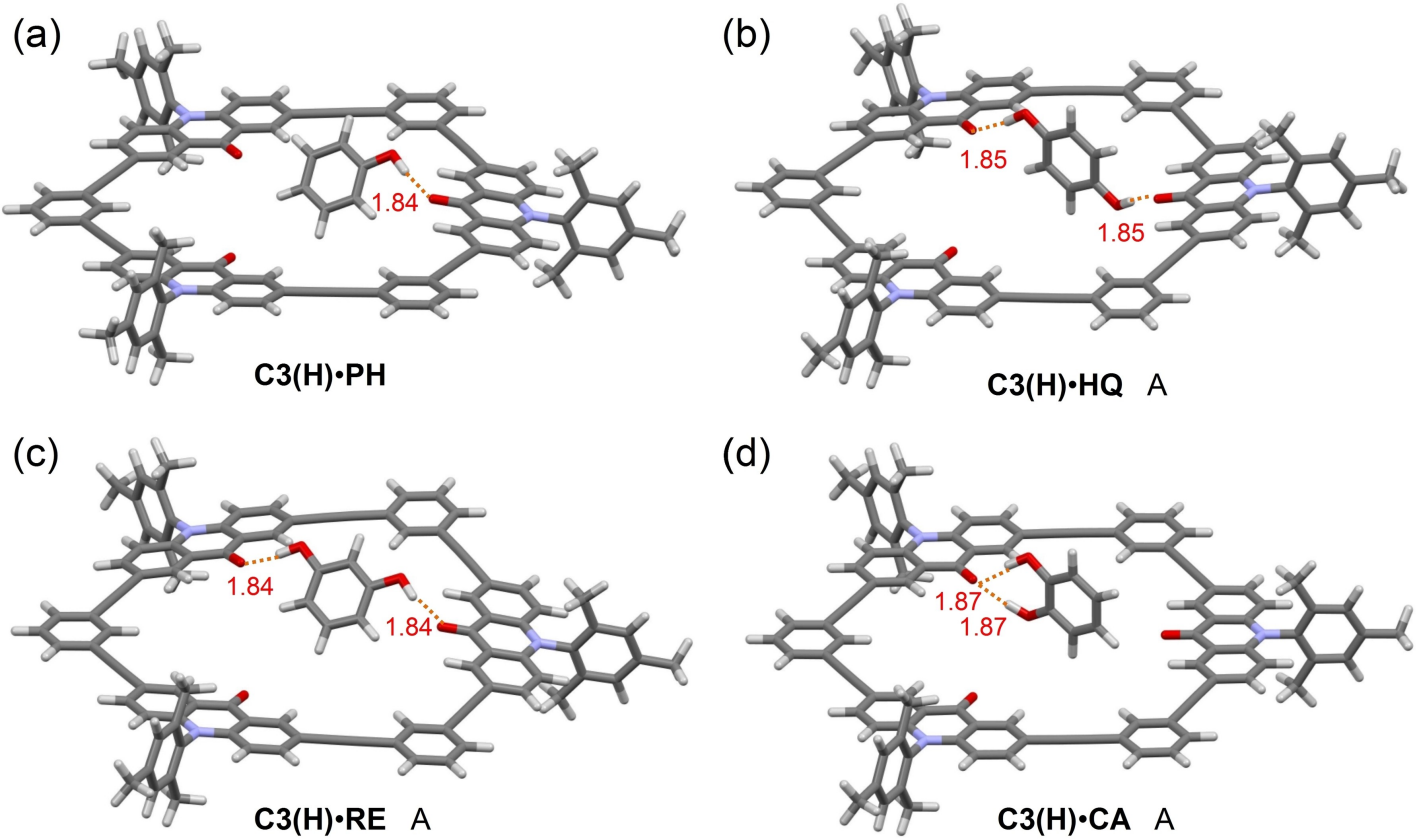
Optimized structures of (a) **C3(H) ⋅ PH**, (b) **C3(H) ⋅ HQ**, (c) **C3(H) ⋅ RE**, and (d) **C3(H) ⋅ CA** 1 : 1 complexes calculated at B3LYP/6‐31G(d) level. Red values in the structures are the OH⋅⋅⋅O=C distances in Å. As for the **HQ**, **RE**, and **CA** complexes, only the most stable complexes are shown. Other structures are provided in Figure S13.

In order to obtain further information on the host‐guest interactions, we calculated the structures and energies of the complexes of **C3(H)** by the DFT method. We preliminary searched possible structures of 1 : 1 complexes by CONFLEX program (Figure S12).^[27]^ The major structures were further optimized at the B3LYP/6‐31G(d) level in the gas phase. Selected structures are shown in Figure 8. Table 2 lists complexation energies (−Δ*E*, −Δ*H*_298_, and −Δ*G*_298_) obtained from the thermodynamic parameters calculated for the complex and the free host and guest molecules. To estimate the energy of a single hydrogen bond, we also calculated complex **C3(H) ⋅ PH**, where the stabilization energy was −Δ*E* 48.1 kJ mol^−1^ (Figure 8a). Because this energy is almost the same as that of 10‐mesitylacridone(**1**) ⋅ **PH** complex (48.6 kJ mol^−1^), a carbonyl group in **C3(H)** behaves independently toward a **PH** molecule as a hydrogen bond acceptor. These values are in the range of moderate hydrogen bonds (17–63 kJ mol^−1^) classified by Emsley^[28]^ as exemplified by the calculated hydrogen bond energy of *N*‐methylacetamide ⋅ **PH** complex (49.7 kJ mol^−1^).^[29]^

**Table 2:** Calculated thermodynamic parameters for complexation of **C3(H)** with phenolic guests, phenol (**PH**), hydroquinone (**HQ**), resorcinol (**RE**), and catechol (**CA**), at the B3LYP/6‐31G(d) level.^[a]^

	−Δ*E* [kJ mol^−1^]	−Δ*H*_298_ [kJ mol^−1^]	−Δ*G*_298_ [kJ mol^−1^]	−Δ*E*_H_ [kJ mol^−1^]^[b]^	−Δ*E*_G_ [kJ mol^−1^]^[c]^
**PH**	48.1	40.9	−13.2	−1.9	−0.9
**PH** ^[d]^	48.6	41.5	1.9	–	–
**HQ** A	73.8	63.1	15.9	−3.1	−2.4
**HQ** B	67.0	57.3	7.4	–	–
**HQ** C	63.6	53.6	9.5	–	–
**RE** A	80.0	71.6	15.9	−3.4	−2.2
**RE** B	66.7	59.0	7.8	–	–
**RE** C	64.8	57.2	11.5	–	–
**CA** A	45.8	39.2	−10.6	−3.1	−33.0
**CA** B	35.7	29.8	−7.8	–	–

[a] Structures are shown in Figures 8 and S13. Full thermodynamic data are provided in Table S4. [b] Energies required for the structural change of the host moiety upon complexation. [c] Energies required for the structural change of the guest moiety upon complexation. [d] Complex with 10‐mesitylacridone (**1**).

**Figure 9:**
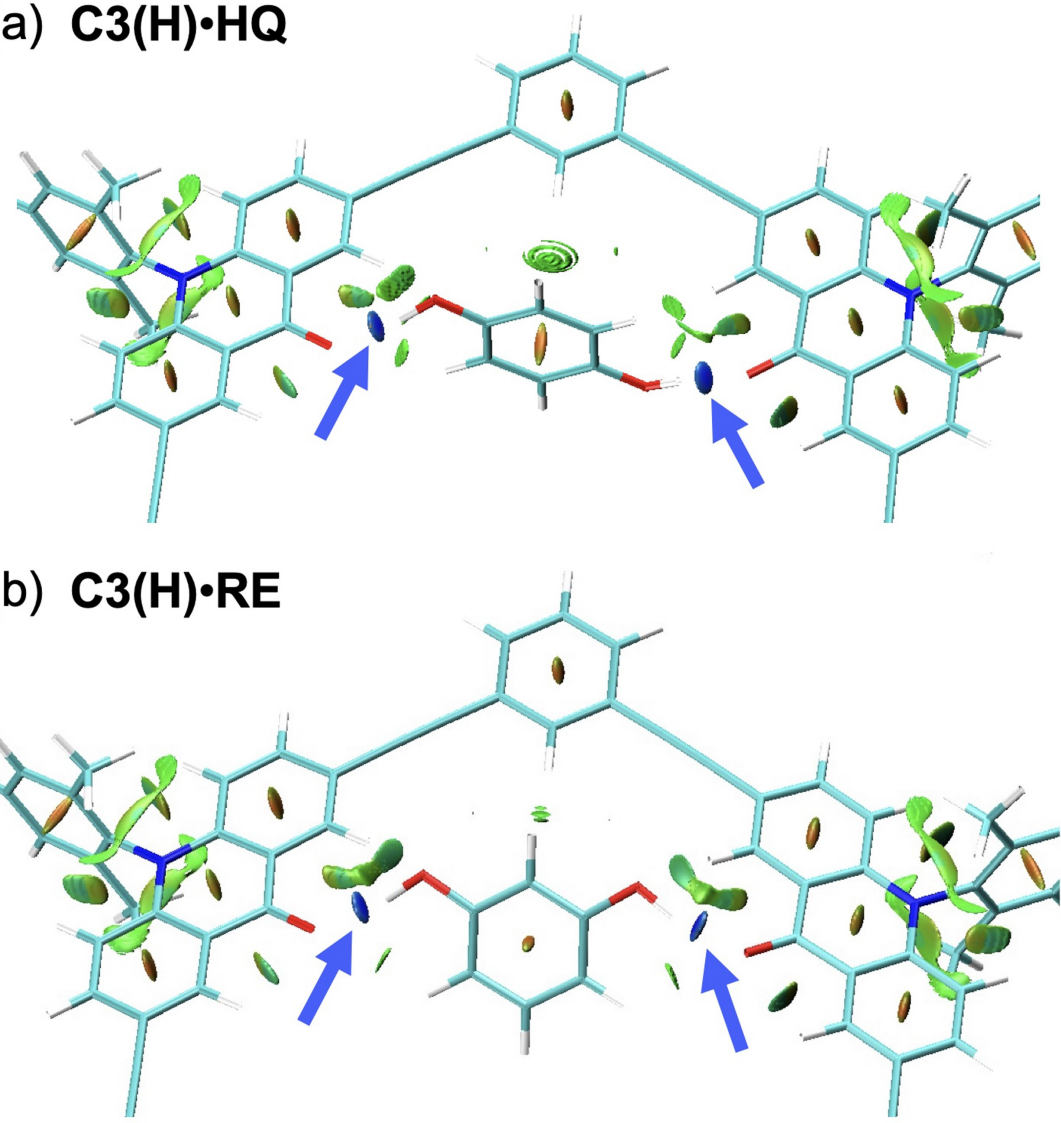
Noncovalent interaction (NCI) plots for partial structures around guest molecules of (a) **C3(H) ⋅ HQ** A and (b) **C3(H) ⋅ RE** A 1 : 1 complexes calculated at B3LYP/6‐31G(d) level (isosurface value 0.6). The colors of the isosurface are blue (attractive interactions), green (weak vdW interactions), and red (repulsive interactions). Blue arrows indicate isosurfaces for hydrogen bonds. NCI plots of other complexes are provided in Figure S14. The two dimensional reduced density gradient (RDG) vs. sign(*λ*_2_)*ρ* plots are shown in Figure S15.

We found three energy‐minimum structures of **C3(H) ⋅ HQ** with two OH⋅⋅⋅O=C hydrogen bonds, and their bond distances (1.85–1.89 Å) were in the range of moderate hydrogen bonds (1.5–2.2 Å).^[28]^ In the global‐minimum structure A (Figure 8b), the two OH groups, which are coplanar with the benzene plane and *anti* with each other, bond to different C=O groups from the opposite sides of the macrocyclic plane. As a result, the benzene ring of the **HQ** guest is nearly standing relative to the macrocyclic plane. Upon complexation, the destabilizations by structural changes of the host (−Δ*E*_H_ 3.1 kJ mol^−1^) and guest (−Δ*E*_G_ 2.4 kJ mol^−1^) moieties are rather insignificant as shown in Table 2. The association energy of **C3(H) ⋅ HQ**, 73.8 kJ mol^−1^, is smaller than twice that of **C3(H) ⋅ PH**. This finding means that each hydrogen bond cannot be maximized for the structural constraint in the cyclic host‐guest system. The significantly small −Δ*G* value (15.9 kJ mol^−1^) relative to the −Δ*E* value is attributed to a large contribution of the entropic term resulting from the formation of one complex from two independent molecules. The presence of hydrogen bonds in this complex was visualized by the noncovalent interaction (NCI) plot (Figure 9a).[^24e^, ^30^] The blue isosurfaces between the interacting O and H atoms indicate strong attractive interactions due to the hydrogen bonds in these regions. The other two structures, B and C, which have different orientations between the host and guest molecules, are less stable than the global minimum A.

The complexes with **RE** and **CA** were similarly calculated. The most stable structures are shown in Figure 8. For **C3(H) ⋅ RE**, we obtained three energy‐minimum structures, where the two OH groups interact with different C=O moieties in various modes. The association energy of the most stable **C3(H) ⋅ RE** complex A (80.0 kJ mol^−1^) is slightly larger than that of **C3(H) ⋅ HQ** (73.8 kJ mol^−1^), but the free energies of association are identical for the two complexes. The presence of hydrogen bonds is supported by the NCI plot of **C3(H) ⋅ RE** as shown in Figure 9b. For **C3(H) ⋅ CA**, the two OH groups almost equally interact with one C=O moiety (1.87 Å), because the distance between them is too short to bridge two C=O moieties in the macrocyclic host. The association energy of the most stable **C3(H) ⋅ CA** complex A (45.8 kJ mol^−1^) is much smaller than those of the other complexes. This small association energy is attributed to not only the contribution of only one C=O moiety but also the destabilization of the guest molecule (33 kJ mol^−1^). The latter factor involves the dissociation of an intramolecular hydrogen bond in **CA**, which requires at least 15–20 kJ mol^−1^ as suggested by the DFT calculation.^[31]^ These calculated data are consistent with the tendency in the observed association constants, **HQ**≈**RE**>**CA**.

## Conclusion

We synthesized new acridone incorporated arylene–ethynylene cyclic oligomers by Sonogashira coupling. Cyclic trimers had a planar and rigid macrocyclic framework, where the cavity was surrounded by three carbonyl groups. In contrast, cyclic tetramers had a nonplanar and nonrigid macrocyclic framework. Cyclic trimer **C3(Mes)** associates with phenolic guest molecules, **HQ** and **RE**, in 1 : 1 ratio in solutions. Their association constants were ca. 1.0×10^3^ L mol^−1^, as determined by the ^1^H NMR measurements. The DFT calculations revealed that the complexes were stabilized by two OH⋅⋅⋅O=C hydrogen bonds between the host and guest molecules. Because the guest molecules examined in this study are smaller than the cavity size, the cavity space (size‐fitting) as well as the noninteracting carbonyl group (multipoint recognition) is not fully utilized well for the molecular recognition. We are seeking for such guest molecules that can bind to the macrocyclic hosts tightly or selectively. We will be able to transform carbonyl moieties in acridone units into other groups to tune the size and the electronic demand of the cavity. Further studies of the synthesis of other acridone cyclic oligomers and their supramolecular properties by applying the polar and donor characters are in progress.

## Experimental Section

The synthetic procedures of selected key macrocyclization steps and the physical and spectroscopic data of the final products are described here. Other materials are provided in Supporting Information.

**Macrocyclization of 11 b and 13**. In a 100 mL round‐bottom flask, a mixture of **11 b** (23.8 mg, 31.1 μmol), **13** (24.4 mg, 30.6 μmol), Pd(PPh_3_)_4_ (7.3 mg, 6.3 μmol), and CuI (1.1 mg, 5.8 μmol) in a degassed mixture of Et_3_N (15 mL) and THF (15 mL) was heated at 60 °C for 22 h under N_2_ atmosphere. After cooling to room temperature, the mixture was treated with water (100 mL) and the organic materials were extracted with CHCl_3_ (30 mL×3). The combined organic layer was dried over Na_2_SO_4_ and evaporated. The crude product was purified by chromatography on silica gel with CHCl_3_/EtOAc 1 : 1 eluent and recycle gel permeation chromatography (GPC) with CHCl_3_ eluent to give 4.9 mg (12%) of **C3(H)** as a yellow solid. **C3(H)**: mp 285–286 °C; *R*_f_ 0.40 (CH_2_Cl_2_); ^1^H NMR (500 MHz, CDCl_3_): *δ*=8.90 (d, *J*=2.0 Hz, 6H), 7.88 (s, 3H), 7.64 (dd, *J*=9.0, 2.0 Hz, 6H), 7.48 (dd, *J*=8.0, 2.0 Hz, 6H), 7.33 (t, *J=*8.0 Hz, 3H), 7.19 (s, 6H), 6.70 (d, *J*=9.0 Hz, 6H), 2.48 (s, 9H), 1.88 (s, 18H); ^13^C NMR (125 MHz, CDCl_3_): *δ*=176.83, 145.95, 141.26, 140.00, 137.15, 136.46, 133.03, 132.19, 130.88, 130.67, 128.50, 123.89, 122.36, 117.06, 116.03, 89.59, 89.13, 21.42, 17.38; UV‐vis (CHCl_3_, 1.0×10^−5^ mol L^−1^): *λ*_max_ (*ϵ*) 421 (20600), 400 (23800), 360 (178000), 349 (162000), 326 nm (205000 L mol^−1^ cm^−1^); FL (CHCl_3_, 1.0×10^−5^ mol L^−1^): *λ*_em_ 439 nm, *λ*_ex_ 422 nm (*Φ*_f_ 0.084); IR (KBr): $\tilde{v}$ =1646 (C=O), 2212 cm^−1^ (C≡C); HRMS (FAB): *m/z* calcd for C_96_H_63_N_3_O_3_: 1305.4869 [*M*]^+^; found 1305.4867.

**Macrocyclization of 11 a and 13 (copper‐free condition)**. In a 50 mL round‐bottom flask, a mixture of **11 a** (20.2 mg, 30.1 μmol), **13** (24.0 mg, 30.1 μmol), PdCl_2_(MeCN)_2_ (1.1 mg, 4.1 μmol), SPhos (2.7 mg, 6.7 μmol), and Cs_2_CO_3_ (43.5 mg, 134 μmol) in degassed DMF (10 mL) was heated at 90 °C for 22 h under N_2_ atmosphere. After cooling to room temperature, the mixture was treated with water (100 mL). The organic materials were extracted with CHCl_3_ (30 mL×3). The combined organic layer was dried over Na_2_SO_4_ and evaporated. The crude product was purified by chromatography on silica gel with CH_2_Cl_2_/EtOAc 1 : 1 eluent and recycle GPC with CHCl_3_ eluent to give 5.8 mg (15%) of **C3(H)** as a yellow solid.

**Macrocyclization of 3 and 9(H) (typical procedure)**. In a 100 mL round‐bottom flask, a mixture of **3** (100 mg, 199 μmol), 1,3*‐*dibromobenzene (**9(H)**, 24.1 μL, 200 μmol), PdCl_2_(MeCN)_2_ (5.2 mg, 20 μmol), SPhos (16.5 mg, 40.2 μmol), and Cs_2_CO_3_ (259 mg, 796 μmol) in degassed DMF (67 mL) was heated at 90 °C for 23 h under N_2_ atmosphere. After cooling to room temperature, the mixture was treated with water (50 mL). The organic materials were extracted with CH_2_Cl_2_ (30 mL×3). The combined organic layer was dried over Na_2_SO_4_ and evaporated. The crude product was purified by chromatography on silica gel with CH_2_Cl_2_/EtOAc 30 : 1 eluent and recycle GPC with CHCl_3_ eluent (Figure S1) to give 13.3 mg (15%) of **C3(H)** as a yellow solid.

**Macrocyclization of 3 and 9(Mes)**. This reaction was carried out according to the typical procedure from **3** (60.7 mg, 120 μmol) and **9(Mes)** (35.5 mg, 100 μmol). The crude products were separated by chromatography on silica gel with CH_2_Cl_2_ eluent and recycle GPC with CHCl_3_ eluent (Figure S2) to give **C3(Mes)** and **C4(Mes)** as yellow solids. **C3(Mes)**: Yield 5.5 mg (9.9%); mp 301–304 °C (dec.); *R*_f_ 0.65 (CH_2_Cl_2_); ^1^H NMR (500 MHz, CDCl_3_): *δ*=8.93 (d, *J=*2.0 Hz, 6H), 7.87 (s, 3H), 7.60 (dd, *J=*9.0, 2.0 Hz, 6H), 7.28 (d, *J=*2.0 Hz, 6H), 7.17 (s, 6H), 6.94 (s, 6H), 6.69 (d, *J=*9.0 Hz, 6H), 2.46 (s, 9H), 2.33 (s, 9H), 2.05 (s, 18H), 1.87 (s, 18H); ^13^C NMR (125 MHz, CDCl_3_): *δ*=176.85, 141.72, 141.25, 139.99, 137.55, 137.10, 136.45, 136.05, 133.86, 132.99, 132.20, 131.83, 130.66, 128.21, 124.07, 122.35, 117.02, 116.05, 89.67, 89.27, 21.40, 21.20, 20.92, 17.37 (one aromatic peak was overlapped); UV‐vis (CHCl_3_, 1.0×10^−5^ mol L^−1^): *λ*_max_ (*ϵ*) 422 (16800), 400 (20800), 362 (179000), 351 (161000), 327 nm (188000 L mol^−1^ cm^−1^); FL (CHCl_3_, 1.0×10^−5^ mol L^−1^): *λ*_em_ 439 nm, *λ*_ex_ 423 nm (*Φ*_f_ 0.091); IR (KBr): $\tilde{v}$ =1650 (C=O), 2211 cm^−1^ (C≡C); HRMS (FAB): *m/z* calcd for C_123_H_93_N_3_O_3_: 1659.7217 [*M*]^+^; found 1659.7217. **C4(Mes)**: Yield 1.9 mg (3.4%); mp 288–290 °C (dec.); *R*_f_ 0.90 (CH_2_Cl_2_); ^1^H NMR (500 MHz, CDCl_3_): *δ*=8.81 (d, *J*=2.0 Hz, 8H), 7.74 (t, *J*=2.0 Hz, 4H), 7.62 (dd, *J*=9.0, 2.0 Hz, 8H), 7.28 (d, *J*=2.0 Hz, 8H), 7.17 (s, 8H), 6.94 (s, 8H), 6.69 (d, *J*=9.0 Hz, 8H), 2.45 (s, 12H), 2.32 (s, 12H), 2.05 (s, 24H), 1.85 (s, 24H); ^13^C NMR (125 MHz, CDCl_3_): *δ*=176.86, 141.74, 141.26, 140.02, 137.47, 137.14, 137.08, 136.91, 136.05, 133.24, 132.99, 132.43, 131.53, 130.68, 128.25, 123.98, 122.25, 117.04, 116.11, 89.45, 89.11, 21.40, 21.19, 20.93, 17.35; UV‐vis (CHCl_3_, 1.0×10^−5^ mol L^−1^): *λ*_max_ (*ϵ*) 418 (26500), 398 (30500), 355 (189000), 343 (170000), 323 nm (202000 L mol^−1^ cm^−1^); FL (CHCl_3_, 1.0×10^−5^ mol L^−1^): *λ*_em_ 438 nm, *λ*_ex_ 419 nm (*Φ*_f_ 0.093); IR (KBr):$\tilde{v}$ =1650 (C=O), 2207 cm^−1^ (C≡C); HRMS (FAB): *m/z* calcd for C_164_H_124_N_4_O_4_: 2212.9623 [*M*]^+^; found 2212.9624.

**Macrocyclization of 3 and 9(Tip)**. This reaction was carried out according to the typical procedure from **3** (101 mg, 200 μmol), **9(Tip)** (87.7 mg, 200 μmol). The crude products were separated by chromatography on silica gel with CH_2_Cl_2_ eluent and recycle GPC with CHCl_3_ eluent (Figure S3) to give **C3(Tip)** and **C4(Tip)** as yellow solids. **C3(Tip)**: Yield 7.2 mg (5.6%); mp 318–320 °C (dec.); *R*_f_ 0.83 (CH_2_Cl_2_); ^1^H NMR (500 MHz, CDCl_3_): *δ*=8.94 (d, *J*=2.0 Hz, 6H), 7.88 (t, *J*=2.0 Hz, 3H), 7.60 (dd, *J*=9.0, 2.0 Hz, 6H), 7.32 (d, *J*=2.0 Hz, 6H), 7.17 (s, 6H), 7.06 (s, 6H), 6.69 (d, *J*=9.0 Hz, 6H), 2.94 (sept, *J*=7.0 Hz, 3H), 2.66 (sept, *J*=7.0 Hz, 6H), 2.45 (s, 9H), 1.86 (s, 18H), 1.30 (d, *J*=7.0 Hz, 18H), 1.12 (d, *J*=7.0 Hz, 36H); ^13^C NMR (125 MHz, CDCl_3_): *δ*=176.86, 148.48, 146.66, 141.42, 141.25, 139.99, 137.09, 136.50, 135.62, 133.79, 133.01, 132.22, 130.66, 123.61, 122.37, 120.71, 117.05, 116.04, 89.60, 89.41, 34.48, 30.43, 24.40, 24.25, 21.40, 17.35 (one aromatic peak was overlapped); UV‐vis (CHCl_3_, 1.0×10^−5^ mol L^−1^): *λ*_max_ (*ϵ*) 422 (19300), 400 (24300), 363 (206000), 352 (183000), 328 nm (210000 L mol^−1^ cm^−1^); FL (CHCl_3_, 1.0×10^−5^ mol L^−1^): *λ*_em_ 439 nm, *λ*_ex_ 423 nm (*Φ*_f_ 0.081); HRMS (FAB): *m/z* calcd for C_141_H_129_N_3_O_3_: 1912.0034 [*M*]^+^; found 1912.0078. **C4(Tip)**: Yield 2.8 mg (2.2%); mp 307–310 °C (dec.); *R*_f_ 0.95 (CH_2_Cl_2_); ^1^H NMR (500 MHz, CDCl_3_): *δ*=8.81 (d, *J*=2.0 Hz, 8H), 7.75 (t, *J*=2.0 Hz, 4H), 7.62 (dd, *J*=9.0, 2.0 Hz, 8H), 7.32 (d, *J*=2.0 Hz, 8H), 7.16 (s, 8H), 7.06 (s, 8H), 6.69 (d, *J*=9.1 Hz, 8H), 2.93 (sept, *J*=7.0 Hz, 4H), 2.66 (sept, *J*=7.0 Hz, 8H), 2.45 (s, 12H), 1.85 (s, 24H), 1.30 (d, *J=*7.0 Hz, 24H), 1.12 (d, *J*=7.0 Hz, 48H); ^13^C NMR (125 MHz, CDCl_3_): *δ*=176.87, 148.49, 146.66, 141.44, 141.25, 140.02, 137.06, 136.91, 135.52, 133.20, 132.97, 132.75, 131.56, 130.67, 123.50, 122.24, 120.75, 117.05, 116.09, 89.37, 89.21, 34.46, 30.43, 24.41, 24.23, 21.40, 17.34; UV‐vis (CHCl_3_, 1.0×10^−5^ mol L^−1^): *λ*_max_ (*ϵ*) 419 (26200), 398 (30400), 355 (203000), 323 nm (213000 L mol^−1^ cm^−1^); FL (CHCl_3_, 1.0×10^−5^ mol L^−1^): *λ*_em_ 438 nm, *λ*_ex_ 420 nm (*Φ*_f_ 0.092); HRMS (FAB): *m/z* calcd for C_188_H_172_N_4_O_4_: 2549.3379 [*M*]^+^; found 2549.3330.

**X‐ray crystallography**. Deposition numbers 2171120 for **C3(H)**, 2171123 for **C4(Mes)**, and 2171121 for **C3(Tip)** contain the supplementary crystallographic data for this paper. These data are provided free of charge by the joint Cambridge Crystallographic Data Centre and Fachinformationszentrum Karlsruhe Access Structures service.

**Computational methods**. The conformational search of host‐gest complexes was carried out by using CONFLEX program.^[27]^ The selected stable structures were further optimized by DFT method at B3LYP/6‐31G(d) level with Gaussian 16 program (Figure 8).^[32]^ The frequency analysis was carried out for each optimized structure, giving no imaginary wavenumber for energy minimum structures.

## Conflict of interest

The authors declare no conflict of interest.

1

## Supporting information

As a service to our authors and readers, this journal provides supporting information supplied by the authors. Such materials are peer reviewed and may be re‐organized for online delivery, but are not copy‐edited or typeset. Technical support issues arising from supporting information (other than missing files) should be addressed to the authors.

Supporting Information

## Data Availability

The data that support the findings of this study are available in the supplementary material of this article.
