# Supplementary material for: Synthesis, Structures, and Complexation with Phenolic Guests of Acridone‐Incorporated Arylene–Ethynylene Macrocyclic Compounds
Source: Chem Asian J. 2022 Dec 3;18(1):e202201003. doi: 10.1002/asia.202201003 (PMC10107286; doi:10.1002/asia.202201003)
Supplement: Supplementary file 1 — Supporting Information [file ASIA-18-0-s001.pdf]

CHEMISTRY
AN ASIAN JOURNAL

Supporting Information

Synthesis, Structures, and Complexation with Phenolic Guests of Acridone-Incorporated Arylene–Ethynylene Macrocyclic Compounds

Takashi Komori, Eiji Tsurumaki, and Shinji Toyota*© 2022 The Authors. Chemistry - An Asian Journal published by Wiley-VCH GmbH. This is an open access article under the terms of the Creative Commons Attribution License, which permits use, distribution and reproduction in any medium, provided the original work is properly cited.

Contents

1. Experimental Details **S1**

2. UV-vis and Fluorescence Spectra **S9**

3. IR Spectra **S14**

4. X-ray Crystallography **S16**

5. Computational Chemistry **S18**

6. NMR Measurements with Guest Molecules **S27**

7. NMR Charts **S33**

References **S48**

1. Experimental Details

General. Melting points are uncorrected. NMR spectra were measured on a JEOL JNM-ECX500 spectrometer (^{1}H: 500 MHz, ^{13}C: 125 MHz), or a JEOL JNM-ECZ500 spectrometer (^{1}H: 500 MHz, ^{13}C: 125 MHz). High-resolution FAB mass spectra were measured on a JEOL JMS-700 MStation mass spectrometer. Column chromatography was carried out with Kanto Chemical silica gel 60N (40–50 mesh). Recycle GPC was carried out on a Japan Analytical Industry LC-5060 recycling preparative HPLC system with 20 mmϕ × 600 mm JAIGEL-2HR and 2.5HR columns with $CHCl_3$ as eluent. TLC was carried out with Merck Silica Gel 60 F254 (0.2 mm) plate. UV-vis spectra were measured on a JASCO V-670 spectrometer with a 10 mm cell. Fluorescence spectra were measured on a JASCO FP-6500 spectrofluorometer with a 10 mm cell. Absolute fluorescence quantum yields were recorded on a Hamamatsu photonics C9920-02. Fluorescence lifetimes were measured on a time correlated single-photon counting system (HORIBA Fluoro Cube) with a NanoLED laser (379 nm). IR spectra were measured on a JASCO FT/IR-4100 spectrometer.

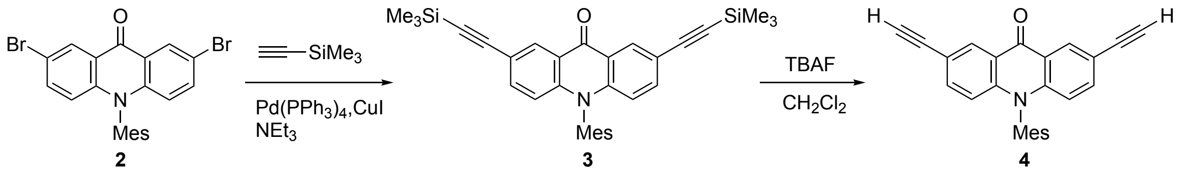

10-Mesityl-2,7-bis[(trimethylsilyl)ethynyl]acridone (3). In a 10 mL Schlenk flask, a mixture of **2** (235 mg, 498 μmol), [S1] (trimethylsilyl)acetylene (283 μL, 2.00 mmol), $Pd(PPh_3)_4$ (29.3 mg, 25.4 μmol), and CuI (4.6 mg, 24.2 μmol) in a degassed Et_3N (2.5 mL) was heated at 70 °C for 4 h under N_2 atmosphere. After cooling to room temperature, the mixture was treated with

water (50 mL) and the organic materials were extracted with CH_2Cl_2 (30 mL × 3). The combined organic layer was dried over Na_2SO_4 and evaporated. The crude product was purified by chromatography on silica gel with hexane/CH_2Cl_2 1:1 eluent to give the desired compound as a yellow solid. Yield 237 mg (94%); mp 253–256 °C; R_f 0.55 (hexane/CH_2Cl_2 1:1); ^{1}H NMR (500 MHz, $CDCl_3$): δ = 8.70 (d, J = 2.0 Hz, 2H), 7.54 (dd, J = 9.0, 2.0 Hz, 2H), 7.15 (s, 2H), 6.62 (d, J = 9.0 Hz, 2H), 2.45 (s, 3H), 1.79 (s, 6H), 0.26 (s, 18H); ^{13}C NMR (125 MHz, $CDCl_3$): δ = 176.83, 141.23, 140.04, 136.96, 132.89, 132.02, 130.67, 121.96, 117.13, 115.90, 104.26, 94.87, 21.39, 17.23, 0.11 (one aromatic peak was overlapped); HRMS (FAB): *m/z* calcd for $C_{32}H_{35}NOSi_2$: 505.2257 [M]$^+$; found 505.2266.

2,7-Diethynyl-10-mesitylacridone (4). In a 30 mL screw tube, a tetrabutylammonium fluoride (TBAF) solution (0.77 mL of 1.0 mol L^{-1} THF solution, 0.77 mmol) was added to a solution of **3** (129 mg, 256 μmol) in CH_2Cl_2 (5.0 mL). The solution was stirred for 30 min at room temperature. After addition of brine (10 mL) and then water (100 mL) to the reaction mixture, the organic materials were extracted with CH_2Cl_2 (50 mL × 3). The organic layer was dried over Na_2SO_4 and evaporated. The crude products were separated by chromatography on silica gel with CH_2Cl_2 to give the desired compound as a yellow solid. Yield 87.4 mg (94%); mp 215–217 °C (dec.); R_f 0.17 (hexane/CH_2Cl_2 1:1); ^{1}H NMR (500 MHz, $CDCl_3$): δ = 8.73 (d, J = 2.0 Hz, 2H), 7.58 (dd, J = 9.0, 2.0 Hz, 2H), 7.16 (s, 2H), 6.66 (d, J = 9.0 Hz, 2H), 3.11 (s, 2H), 2.45 (s, 3H), 1.82 (s, 6H); ^{13}C NMR (125 MHz, $CDCl_3$): δ = 176.70, 141.44, 140.11, 137.22, 136.85, 132.71, 132.04, 130.66, 121.94, 116.10, 116.05, 82.83, 77.71, 21.32, 17.20; HRMS (FAB): *m/z* calcd for $C_{26}H_{19}NO$: 361.1467 [M]$^+$; found 361.1447.

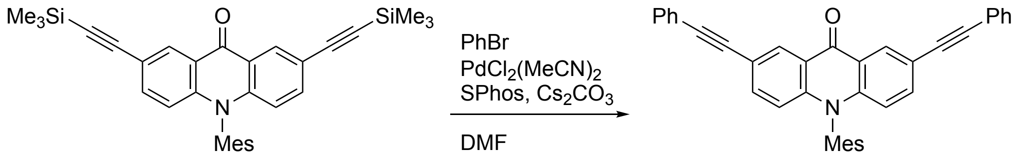

3 **5**

10-Mesityl-2,7-bis(phenylethynyl)acridone (5). In a 10 mL Schlenk flask, a mixture of **3** (50.8 mg, 100 μmol), bromobenzene (52.5 μL, 500 μmol), $PdCl_2(MeCN)_2$ (2.59 mg, 10.0 μmol), SPhos (8.4 mg, 20.5 μmol), and Cs_2CO_3 (128 mg, 392 μmol) in anhydrous DMF (20 mL) was heated at 90 °C for 8 h under N_2 atmosphere. After cooling to room temperature, the mixture was treated with water (50 mL), the organic materials were extracted with CH_2Cl_2 (30 mL × 3), dried over Na_2SO_4, and evaporated. The crude product was purified by chromatography on silica gel with hexane/CH_2Cl_2 3:2 eluent to give the desired compound as a yellow solid. Yield 32.8 mg (64%); mp 110–113 °C; R_f 0.37 (hexane/CH_2Cl_2 1:1); ^{1}H NMR (500 MHz, $CDCl_3$): δ = 8.79 (d, J = 2.0 Hz, 2H), 7.63 (dd, J = 9.0, 2.0 Hz, 2H), 7.56–7.54 (m, 4H), 7.38–7.32 (m, 6H), 7.18 (s, 2H), 6.70 (d, J = 9.0 Hz, 2H), 2.47 (s, 3H), 1.85 (s, 6H); ^{13}C NMR (125 MHz, $CDCl_3$): δ = 176.97, 141.14, 140.07, 137.04, 136.78, 132.97, 131.77, 131.42, 130.69, 128.52,

128.44, 123.33, 122.16, 117.34, 116.11, 89.94, 88.75, 21.40, 17.33; UV-vis ($CHCl_3$, 1.0 × 10^{-5} mol L^{-1}): λ_{max} (ε) 422 (6190), 401 (6290), 383 (5000), 353 nm (42400), 324 nm (50600 L mol^{-1} cm^{-1}); FL ($CHCl_3$, 1.0 × 10^{-5} mol L^{-1}): λ_{em} 440 nm, λ_{ex} 422 nm (Φ_f 0.10); IR (KBr): 1650 cm^{-1} (C=O), 2213 (C≡C); HRMS (FAB): *m/z* calcd for $C_{38}H_{27}NO$: 513.2093 $[M]^+$; found 513.2105.

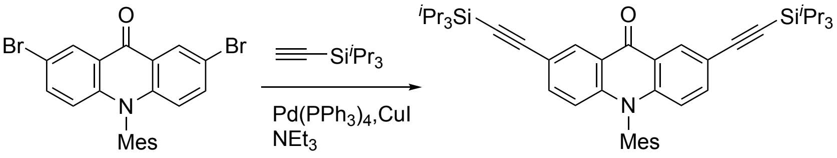

2 **6**

10-Mesityl-2,7-bis[(triisopropylsilyl)ethynyl]acridone (6). In a 20 mL round-bottom flask, a mixture of **2** (1.02 g, 2.16 mmol), (triisopropylsilyl)acetylene (1.42 mL, 6.37 mmol), $Pd(PPh_3)_4$ (120 mg, 103 μmol), and CuI (19.8 mg, 104 μmol) in a degassed Et_3N (20 mL) was heated at 70 °C for 2 h under N_2 atmosphere. After cooling to room temperature, the mixture was treated with water (100 mL) and the organic materials were extracted with CH_2Cl_2 (50 mL × 3). The combined organic layer was dried over Na_2SO_4 and evaporated. The crude product was purified by chromatography on silica gel with hexane/CH_2Cl_2 2:1 eluent to give the desired compound as a yellow solid. Yield 1.27 g (88%); mp 275–277 °C; R_f 0.63 (hexane/CH_2Cl_2 2:1); ^1H NMR (500 MHz, $CDCl_3$): δ = 8.70 (d, J = 2.0 Hz, 2H), 7.56 (dd, J = 9.0, 2.0 Hz, 2H), 7.15 (s, 2H), 6.63 (d, J = 9.0 Hz, 2H), 2.45 (s, 3H), 1.79 (s, 6H), 1.14 (s, 42H); ^{13}C NMR (125 MHz, $CDCl_3$): δ = 176.93, 141.13, 140.02, 137.36, 136.96, 132.92, 131.74, 130.63, 121.94, 117.53, 115.91, 106.18, 91.24, 21.39, 18.83, 17.18, 11.45; HRMS (FAB): *m/z* calcd for $C_{44}H_{59}NOSi_2$: 673.4135 $[M]^+$; found 673.4119.

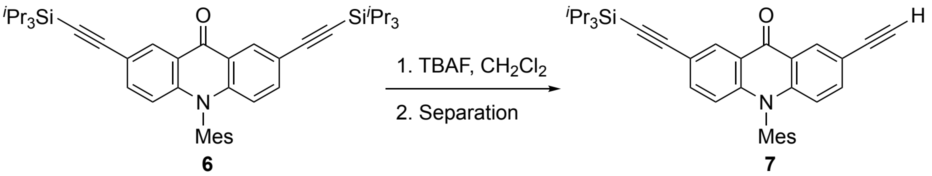

6 **7**

2-Ethynyl-10-mesityl-7-[(triisopropylsilyl)ethynyl]acridone (7). In a 30 mL screw tube, the TBAF solution (0.74 mL, 0.74 mmol) was added to a solution of **6** (500 mg, 0.741 mmol) in CH_2Cl_2 (7.4 mL). This solution was stirred for 30 min at room temperature. After addition of brine (10 mL) and then (100 mL) to the reaction mixture, the organic materials were extracted with CH_2Cl_2 (50 mL × 3). The combined organic layer was dried over Na_2SO_4 and evaporated. The crude products were separated by chromatography on silica gel with hexane/CH_2Cl_2 1:1 eluent to give **6** as the second fraction as a yellow solid. The starting material **5** (209 mg, 42%) was recovered as the first fraction, and compound **6** was obtained as the third fraction (34.1 mg, 13%). Yield 167 mg (43%); mp 205–207 °C; R_f 0.43 (hexane/CH_2Cl_2 1:1); ^1H NMR (500 MHz,

CDCl$_3$): δ = 8.74 (d, J = 2.0 Hz, 1H), 8.70 (d, J = 2.0 Hz, 1H), 7.57 (dt, J = 9.0, 2.0 Hz, 2H), 7.15 (s, 2H), 6.65 (t, J = 9.0 Hz, 2H), 3.11 (s, 1H), 2.45 (s, 3H), 1.81 (s, 6H), 1.14 (s, 21H); ^{13}C NMR (125 MHz, CDCl$_3$): δ = 176.84, 141.46, 141.17, 140.08, 137.39, 137.16, 136.95, 132.85, 132.14, 131.78, 130.66, 122.00, 121.95, 117.65, 116.06, 116.00, 115.94, 106.11, 91.30, 82.93, 77.66, 21.38, 18.82, 17.23, 11.44; HRMS (FAB): *m/z* calcd for $C_{35}H_{39}NOSi$: 517.2801 [M]$^+$; found 517.2809.

3',5'-Dibromo-2,4,6-trimethyl-1,1'-biphenyl [9(Mes)]. This compound was prepared by the known method from 2,4,6-tribromobenzene and 2,4,6-trimethylphenylboronic acid by the Suzuki-Miyaura coupling. [S2]

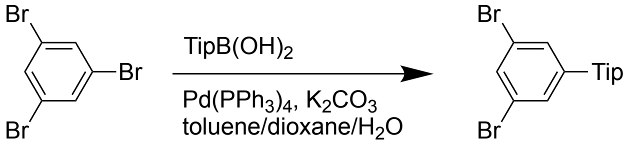

3',5'-Dibromo-2,4,6-triisopropyl-1,1'-biphenyl [9(Tip)]. In a 10 mL Schlenk flask, a mixture of 1,3,5-tribromobenzene (**8**, 186 mg, 591 µmol), 2,4,6-triisopropylphenylboronic acid (49.8 mg, 201 µmol), [S3] K_2CO_3 (41.8 mg, 302 µmol), and Pd(PPh$_3$)$_4$ (11.4 mg, 9.87 µmol) in a degassed mixture of toluene (0.50 mL), 1,4-dioxane (0.20 mL), and H_2O (0.10 mL) was heated to 90 °C for 22 h under N_2 atmosphere. After cooling to room temperature, the solvent was evaporated and the mixture was diluted with hexane (50 mL). The organic layer was washed with water (50 mL), dried over Na_2SO_4, and evaporated. The crude product was purified by chromatography on silica gel with hexane to give the desired compound as a white solid. Yield 37.0 mg (42%); mp 154–156 °C; R_f 0.60 (hexane); ^{1}H NMR (500 MHz, CDCl$_3$): δ = 7.66 (t, J = 2.0 Hz, 1H), 7.29 (d, J = 2.0 Hz, 2H), 7.04 (s, 2H), 2.92 (sept, J = 7.0 Hz, 1H), 2.53 (sept, J = 7.0 Hz, 2H), 1.29 (d, J = 7.0 Hz, 6H), 1.10 (d, J = 7.0 Hz, 12H); ^{13}C NMR (125 MHz, CDCl$_3$): δ = 149.06, 146.42, 144.83, 134.29, 132.41, 131.65, 122.69, 120.91, 34.47, 30.50, 24.31, 24.19; HRMS (FAB): *m/z* calcd for $C_{21}H_{26}{}^{79}Br_2$: 436.0401 [M]$^+$; found 436.0360.

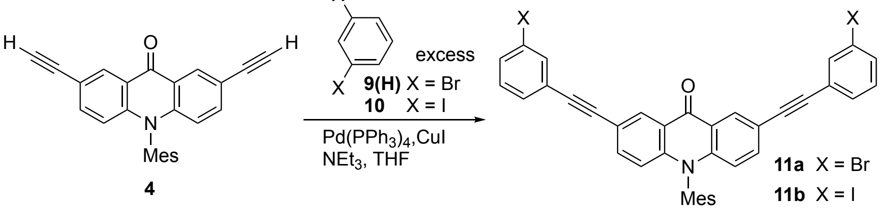

2,7-Bis[(3-bromophenyl)ethynyl]-10-mesitylacridone (11a). In a 10 mL Schlenk flask, a mixture of **4** (92.5 mg, 256 µmol), 1,3-dibromobenzene (**9(H)**, 0.308 mL, 2.56 mmol), Pd(PPh$_3$)$_4$ (29.7 mg, 25.6 µmol), and CuI (4.8 mg, 25.6 µmol) in a degassed mixture of Et_3N

(3.0 mL) and THF (1.0 mL) was heated at 60 °C for 4 h under N_2 atmosphere. After cooling to room temperature, the mixture was treated with water (50 mL) and the organic materials were extracted with CH_2Cl_2 (30 mL × 3). The organic layer was dried over Na_2SO_4 and evaporated. The crude product was purified by chromatography on silica gel with hexane/CH_2Cl_2 1:1 eluent to give the desired compound as a yellow solid. Yield 119 mg (69%); mp 180–182 °C (dec.); R_f 0.48 (hexane/CH_2Cl_2 1:1); ^{1}H NMR (500 MHz, $CDCl_3$): δ = 8.77 (d, J = 2.0 Hz, 2H), 7.70 (t, J = 2.0 Hz, 2H), 7.62 (dd, J = 9.0, 2.0 Hz, 2H), 7.47 (dd, J = 8.0, 1.5 Hz, 4H), 7.23 (t, J = 8.0 Hz, 2H), 7.18 (s, 2H), 6.71 (d, J = 9.0 Hz, 2H), 2.47 (s, 3H), 1.85 (s, 6H); ^{13}C NMR (125 MHz, $CDCl_3$): δ = 176.85, 141.33, 140.15, 136.97, 136.77, 134.39, 132.84, 131.62, 131.57, 130.72, 130.31, 129.97, 125.31, 122.33, 122.16, 116.81, 116.23, 90.02, 88.39, 21.41, 17.33; HRMS (FAB): *m/z* calcd for $C_{38}H_{25}{}^{79}Br_2NO$: 669.0303 $[M]^+$; found 669.0286.

2,7-Bis[(3-iodophenyl)ethynyl]-10-mesitylacridone (11b). In a 10 mL Schlenk flask, a mixture of **4** (73.1 mg, 202 µmol), 1,3-diiodobenzene (**10**, 330 mg, 1.00 mmol), $Pd(PPh_3)_4$ (23.0 mg, 19.9 µmol), and CuI (3.7 mg, 19.4 µmol) in a degassed mixture of Et_3N (1.5 mL) and THF (0.5 mL) was heated at 60 °C for 5 h under N_2 atmosphere. After cooling to room temperature, the mixture was treated with water (50 mL) and the organic materials were extracted with CH_2Cl_2 (30 mL × 3). The combined organic layer was dried over Na_2SO_4 and evaporated. The crude product was purified by chromatography on silica gel with hexane/CH_2Cl_2 1:1 eluent to give the desired compound as a yellow solid. Yield 133 mg (86%); mp 165–167 °C (dec.); R_f 0.50 (hexane/CH_2Cl_2 1:1); ^{1}H NMR (500 MHz, $CDCl_3$): δ = 8.77 (d, J = 2.0 Hz, 2H), 7.91 (s, 2H), 7.67 (d, J = 9.0, 2H), 7.61 (dd, J = 9.0, 2.0 Hz, 2H), 7.51 (d, J = 8.0 Hz, 2H), 7.18 (s, 2H), 7.09 (t, J = 8.0 Hz, 2H), 6.70 (d, J = 9.0 Hz, 2H), 2.47 (s, 3H), 1.85 (s, 6H); ^{13}C NMR (125 MHz, $CDCl_3$): δ = 176.84, 141.31, 140.22, 140.14, 137.42, 136.97, 136.75, 132.85, 131.60, 130.88, 130.72, 130.03, 125.39, 122.16, 116.84, 116.22, 93.87, 90.06, 88.23, 21.40, 17.34; HRMS (FAB): *m/z* calcd for $C_{38}H_{25}I_2NO$: 765.0026 $[M]^+$; found 765.0048.

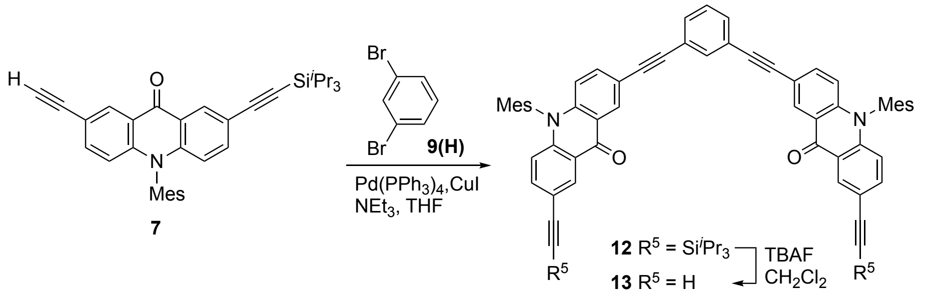

Compound 12. In a 10 mL Schlenk flask, a mixture of **7** (131 mg, 252 µmol), 1,3-dibromobenzene (**9(H)**, 10.1 µL, 84.0 µmol), $Pd(PPh_3)_4$ (10.0 mg, 8.65 µmol), and CuI (1.6 mg, 8.4 µmol) in a degassed mixture of Et_3N (1.4 mL) and THF (0.7 mL) was heated at 60 °C for 23 h under N_2 atmosphere. After cooling to room temperature, the mixture was treated with

water (50 mL) and the organic materials were extracted with CH_2Cl_2 (30 mL × 3). The organic layer was dried over Na_2SO_4 and evaporated. The crude product was purified by chromatography on silica gel with hexane/CH_2Cl_2 1:1 eluent to give the desired compound as a yellow solid. Yield 53.7 mg (58%); mp 210–212 °C; R_f 0.53 (CH_2Cl_2); ^{1}H NMR (500 MHz, $CDCl_3$): δ = 8.78 (d, J = 2.0 Hz, 2H), 8.73 (d, J = 2.0 Hz, 2H), 7.75 (s, 1H), 7.63 (dd, J = 9.0, 2.0 Hz, 2H), 7.57 (dd, J = 9.0, 2.0 Hz, 2H), 7.52 (dd, J = 8.0, 2.0 Hz, 2H), 7.36 (t, J = 8.0 Hz, 1H), 7.16 (s, 4H), 6.70 (d, J = 9.0 Hz, 2H), 6.65 (d, J = 9.0 Hz, 2H), 2.46 (s, 6H), 1.82 (s, 12H), 1.14 (s, 42H); ^{13}C NMR (125 MHz, $CDCl_3$): δ = 176.92, 141.23, 141.14, 140.06, 137.32, 136.97, 136.82, 134.65, 132.90, 131.81, 131.51, 130.66, 128.68, 123.68, 122.10, 122.00, 117.59, 117.01, 116.14, 115.95, 106.17, 91.24, 89.38, 89.11, 21.39, 18.83, 17.84, 11.45 (one aromatic peak was overlapped); HRMS (FAB): *m/z* calcd for $C_{76}H_{80}N_2O_2Si_2$: 1108.5758 $[M]^+$; found 1108.5745.

Compound 13. In a 20 mL screw tube, to a solution of **12** (60.1 mg, 54.2 μmol) in CH_2Cl_2 (1.0 mL), the TBAF solution (0.22 mL, 0.22 mmol) was added. The solution was stirred at room temperature for 3 h. After addition of brine (3 mL) and then water (50 mL) to the reaction mixture, the organic materials were extracted with CH_2Cl_2 (30 mL × 3). The organic layer was dried over Na_2SO, and evaporated. The crude product was purified by chromatography on silica gel with CH_2Cl_2 to give the desired compound as a yellow solid. Yield 37.3 mg (86%); mp 161–163 °C (dec.); R_f 0.53 (CH_2Cl_2); ^{1}H NMR (500 MHz, $CDCl_3$): δ = 8.77 (d, J = 2.0 Hz, 2H), 8.75 (d, J = 2.0 Hz, 2H), 7.73 (s, 1H), 7.63 (dd, J = 9.0, 2.0 Hz, 2H), 7.58 (dd, J = 9.0, 2.0 Hz, 2H), 7.51 (dd, J = 8.0, 2.0 Hz, 2H), 7.36 (t, J = 8.0 Hz, 1H), 7.16 (s, 4H), 6.70 (d, J = 9.0 Hz, 2H), 6.67 (d, J = 9.0 Hz, 2H), 3.11 (s, 2H), 2.45 (s, 6H), 1.83 (s, 12H); ^{13}C NMR (125 MHz, $CDCl_3$): δ = 176.83, 141.47, 141.26, 140.12, 137.19, 136.96, 136.90, 134.63, 132.84, 132.12, 131.52, 131.46, 130.69, 128.69, 123.67, 122.15, 122.01, 117.13, 116.17, 116.11, 116.06, 89.33, 89.14, 82.91, 77.67, 21.39, 17.84; HRMS (FAB): *m/z* calcd for $C_{58}H_{40}N_2O_2$: 796.3090 $[M]^+$; found 796.3100.

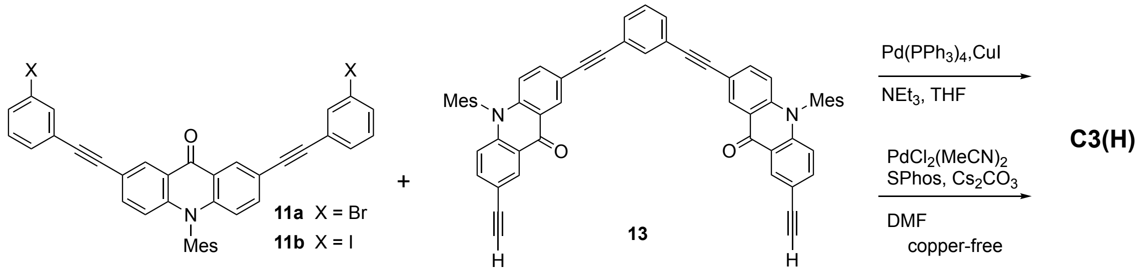

Macrocyclization of 11b and 13. The procedure is described in Experimental Section.

Macrocyclization of 11a and 13. The reaction was similarly carried out. The reaction from **11a** (26.8 mg, 39.9 μmol) and **13** (31.9 mg, 40.0 μmol) in Et_3N (40 mL) THF (40 mL) for 42 h gave no desired product. This reaction gave insoluble material and most of **11a** was recovered.

Macrocyclization of 11a and 13 (copper-free). The procedure is described in Experimental Section.

Macrocyclization of 11b and 13 (copper-free). The reaction was similarly carried out. The reaction from **11b** (11.5 mg, 15.0 μmol), **13** (12.0 mg, 15.1 μmol), and SPhos in DMF (15 mL) for 12 h gave a trace amount of **C3(H)**.

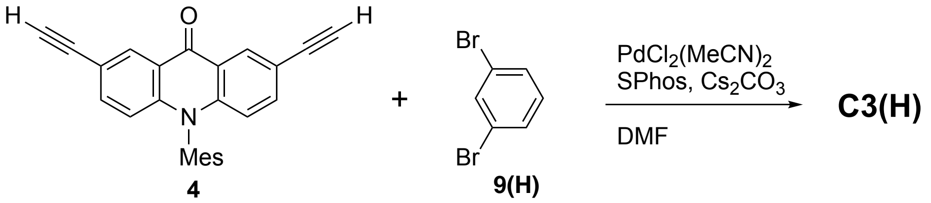

Macrocyclization of 4 and 9(H). The reaction was similarly carried out as described in Experimental Section (macrocyclization of **3** and **9(H)**). The reaction from **4** (179 mg, 496 μmol) and **9(H)** (60.2 μL, 500 μmol) for 23 h gave a trace amount (0.6%) of **C3(H)**.

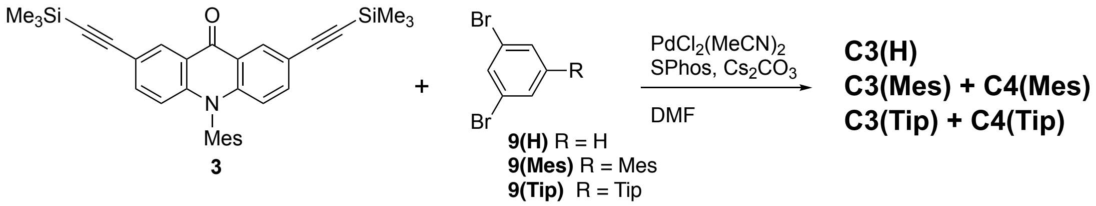

Macrocyclization of 3 and 9(H). The procedure is described in Experimental Section. The chromatogram chart is shown in Figure S1.

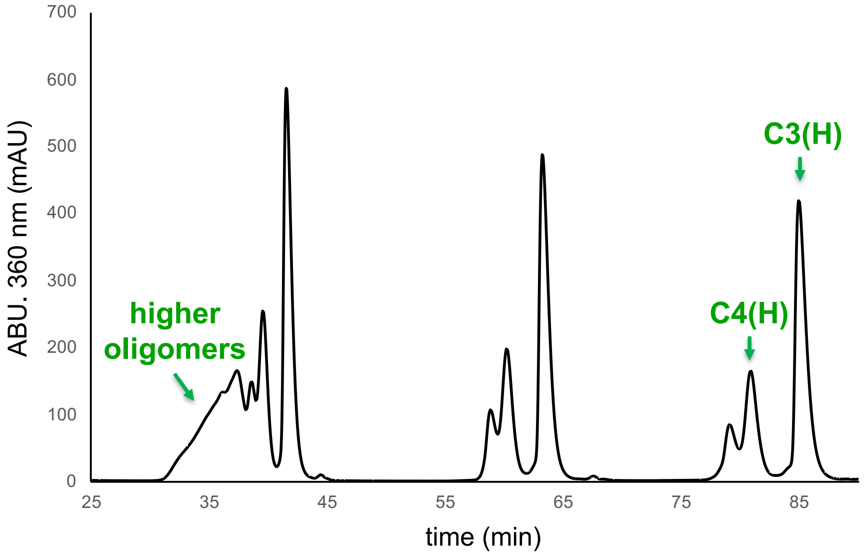

Figure S1. Chromatogram of recycle-GPC ($CHCl_3$ eluent) for separation of the crude products by macrocyclization of **3** and **9(H)**. The elution was monitored by UV absorption at 360 nm. Higher oligomers were removed after the first recycle.

Macrocyclization of 3 and 9(Mes). The procedure is described in Experimental Section. The chromatogram chart is shown in Figure S2.

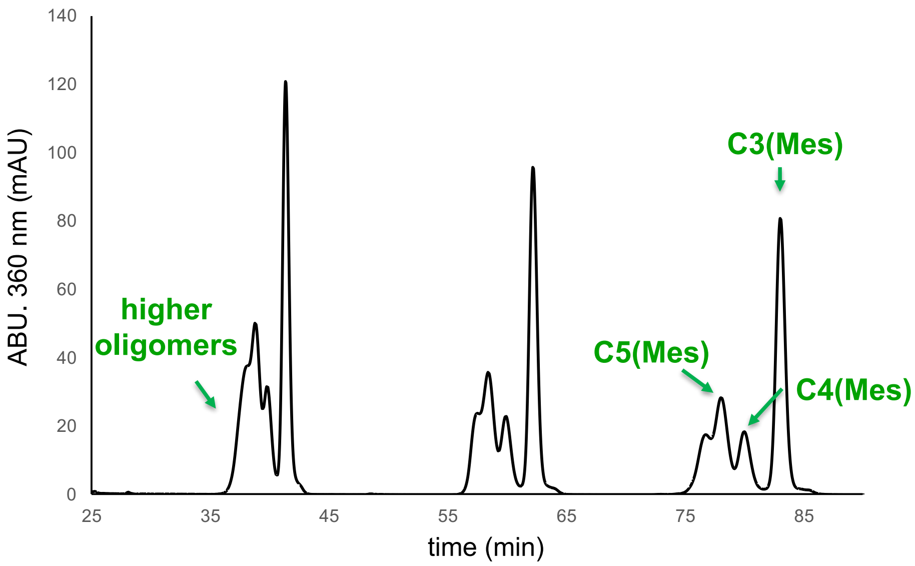

Figure S2. Chromatogram of recycle-GPC ($CHCl_3$ eluent) for separation of the crude products by macrocyclization of **3** and **9(Mes)**. The elution was monitored by UV absorption at 360 nm. Higher oligomers were removed after the first recycle.

Macrocyclization of 3 and 9(Tip). The procedure is described in Experimental Section. The chromatogram chart is shown in Figure S3.

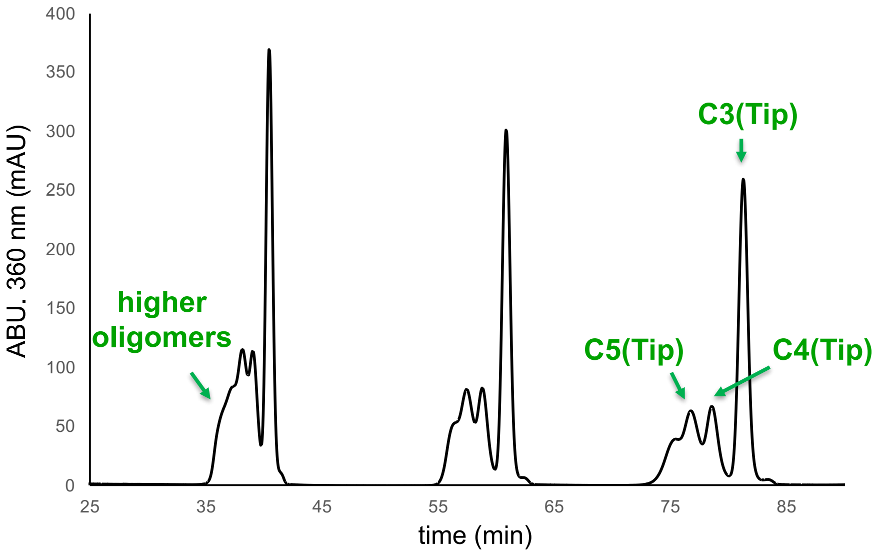

Figure S3. Chromatogram of recycle-GPC ($CHCl_3$ eluent) for separation of the crude products by macrocyclization of **3** and **9(Tip)**. The elution was monitored by UV absorption at 360 nm. Higher oligomers were removed after the first recycle.

2. UV-vis and Fluorescence Spectra

UV-vis and fluorescence spectra of **1**, **5**, **C3(Mes)**, and **C4(Mes)** are shown in Figure 5. The spectra of the other compounds are shown in Figures S4.

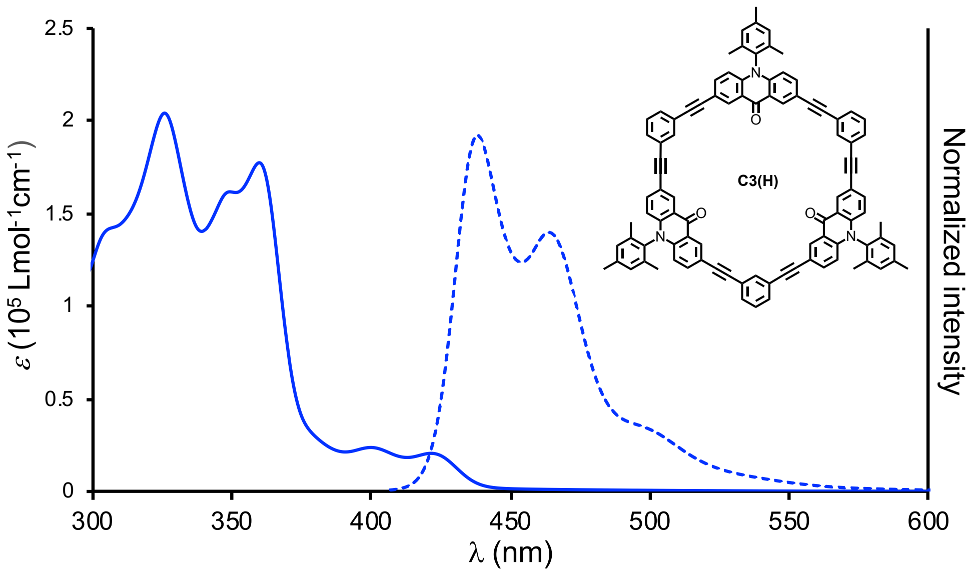

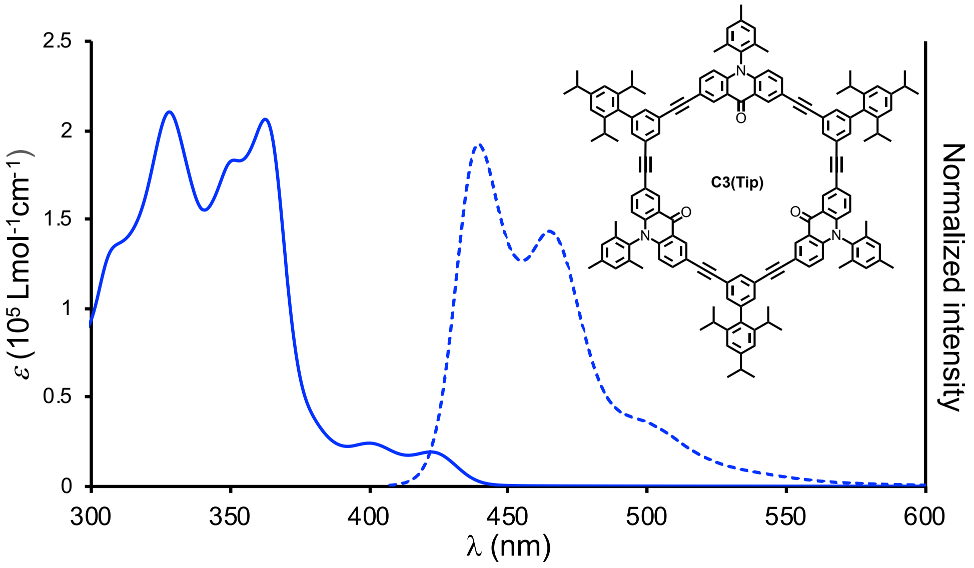

Figure S4. UV-vis (solid line) and fluorescence spectra (dashed line) of **C3(H)**, **C3(Tip)**, and **C4(Tip)** measured in $CHCl_3$. Concentration: 1.0×10^{-5} mol L^{-1}.

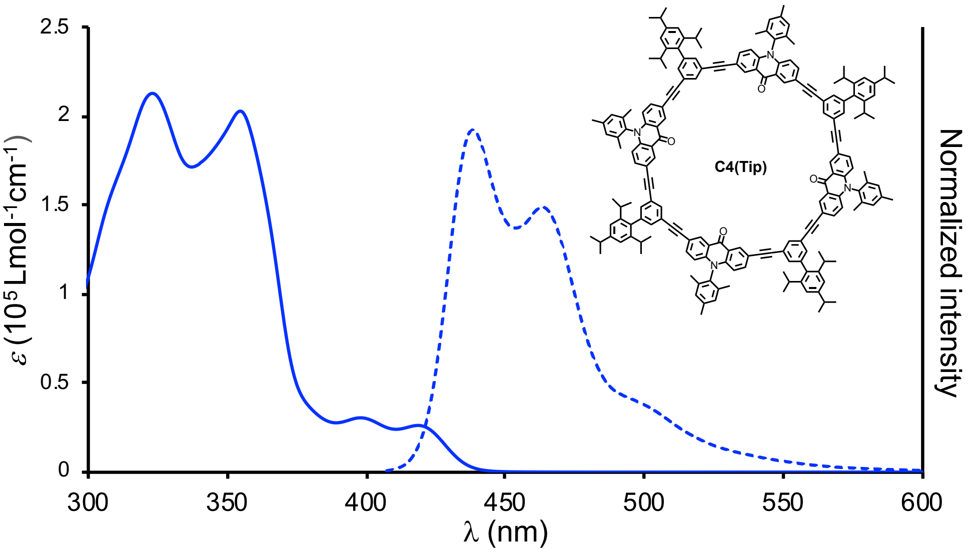

Figure S4 (Continued). UV-vis (solid line) and fluorescence spectra (dashed line) of **C3(H)**, **C3(Tip)**, and **C4(Tip)** measured in $CHCl_3$. Concentration: 1.0×10^{-5} mol L^{-1}.

Solvent effects of UV-vis and FL spectra of ***C3(Tip)***.

The UV-vis and fluorescence spectra of **C3(Tip)** measured in various solvents are shown in Figures S5 and S6. Their spectral data and selected solvent parameters are compiled in Table S1. The correlation of the Stokes shifts vs acceptor number (*AN*) as solvent parameter is shown in Figure S7.

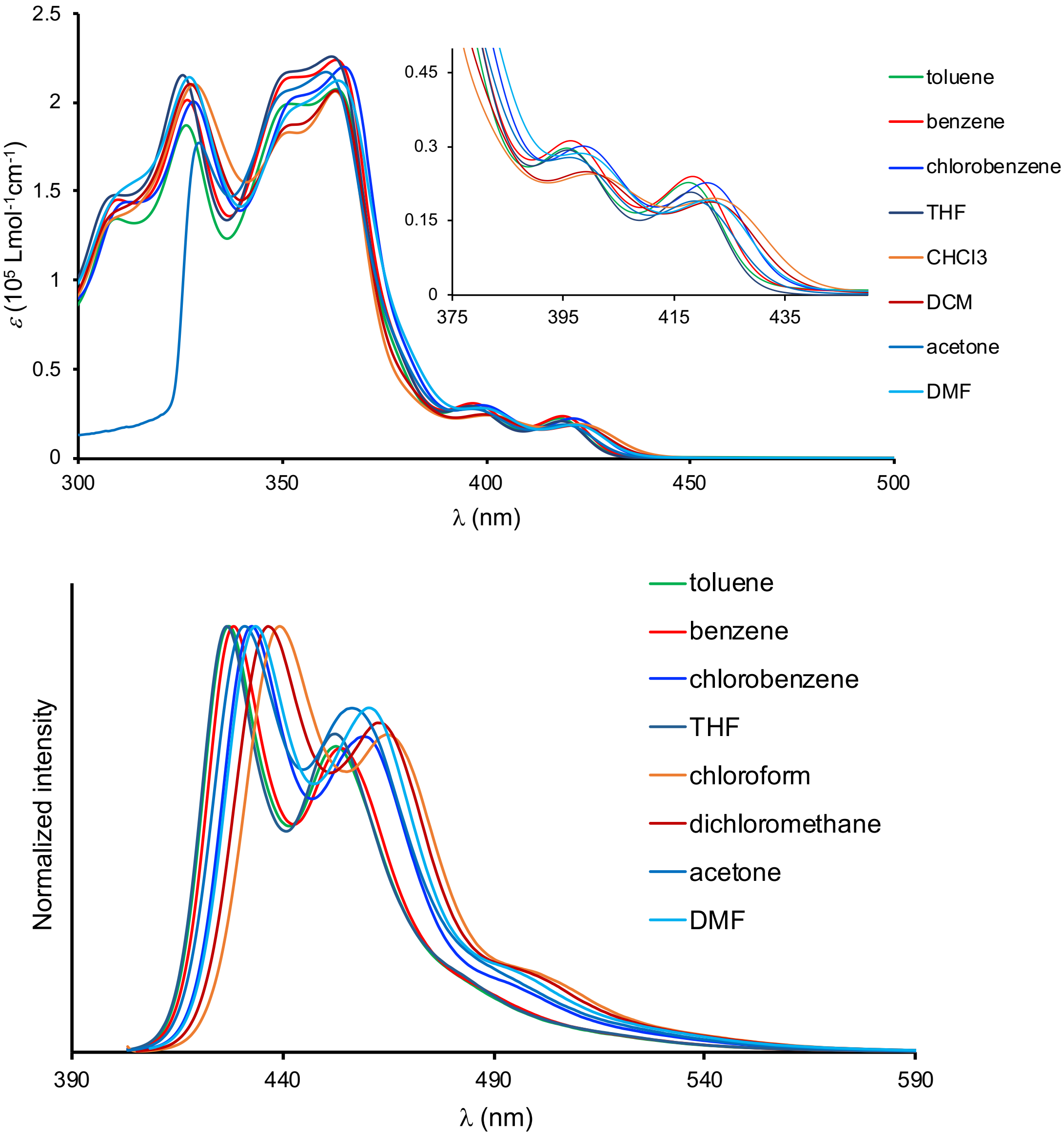

Figure S5. UV-vis (top) and fluorescence spectra (center) of **C3(Tip)** measured in various solvents. Concentration: 1.0×10^{-5} mol L^{-1}.

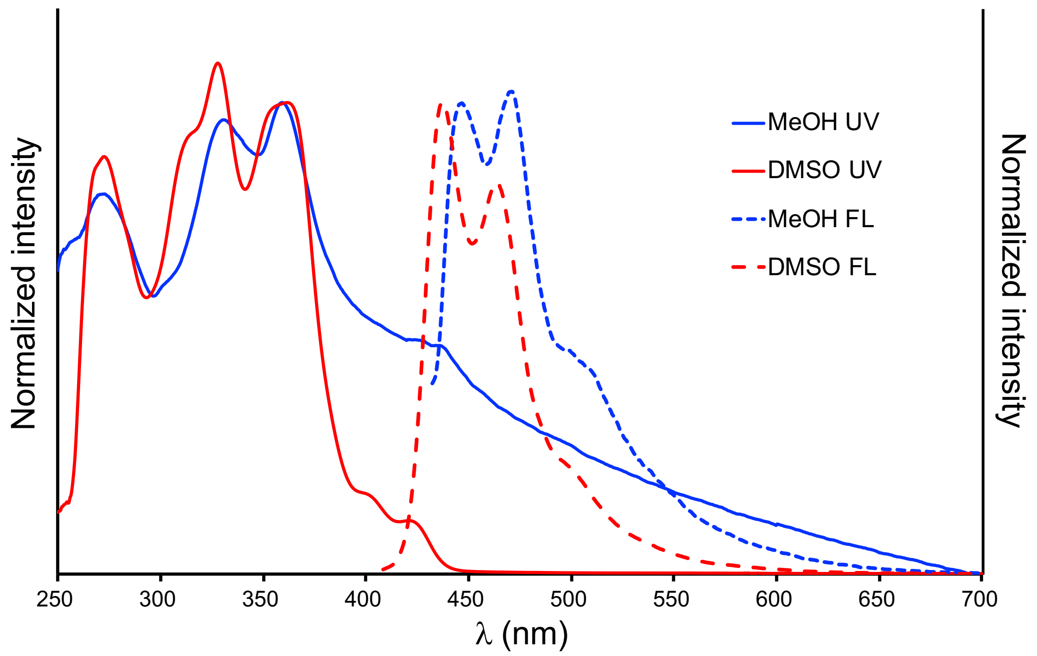

Figure S6. UV-vis (solid line) and fluorescence spectra (dashed line) of **C3(Tip)** measured in DMSO and MeOH. Because of the low solubility of **C3(Tip)** in these solvents, the absorption intensities are expressed as absorbance.

Table S1. UV-vis and fluorescence spectral data of **C3(Tip)** in various solvents and their solvent parameters.

Solvent	$E_T(30)$ [a] [kcal mol^{-1}]	*AN* [b]	UV-vis λ_{max} [nm] (ε [L mol^{-1} cm^{-1}])	FL λ_{em} [nm]	Stokes shift [cm^{-1}]
toluene	33.9	– [c]	418 (22500)	427	530
benzene	34.3	8.2	419 (23700)	428	530
chlorobenzene	36.8	– [c]	421 (22500)	433	630
THF	37.4	8.0	418 (20600)	427	480
$CHCl_3$	39.1	23.1	422 (19300)	439	920
CH_2Cl_2	40.7	20.4	422 (18600)	437	820
acetone	42.2	12.5	419 (18900)	431	660
DMF	43.2	16.0	418 (18900)	434	660
DMSO	45.1	19.3	421 (–) [d]	437	900
methanol	55.4	41.5	424 (–) [d]	447	1200

[a] A empirical parameters of solvent polarity. Ref. S4. [b] Acceptor number. Ref. S5. [c] Not available. [d] Not determined.

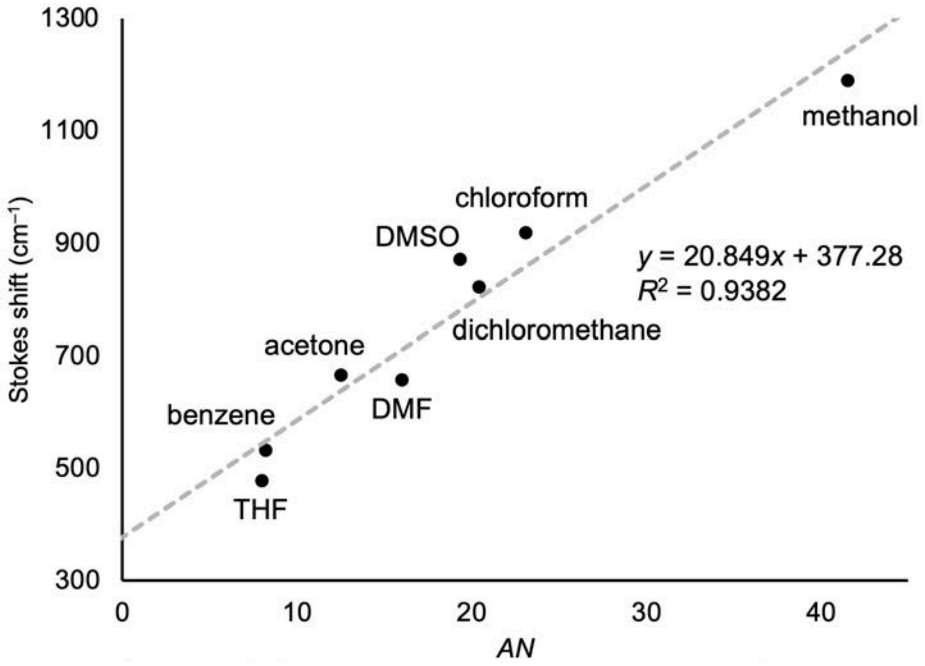

Figure S7. Correlation between acceptor number (*AN*) solvent parameters vs Stokes shifts of **C3(Tip)**.

3. IR Spectra

IR spectra were recorded using a KBr pellets. The observed and calculated IR spectra of **C3(H)** are shown in Figure S8. The observed IR spectra of **C3(H)**, **HQ**, and a mixture of **C3(H)** and **HQ** are shown in Figure S8-2. For **C3(Mes)** and **C4(Mes)**, typical absorptions, ν(C=O) and ν(C≡C), are reported in Experimental Section.

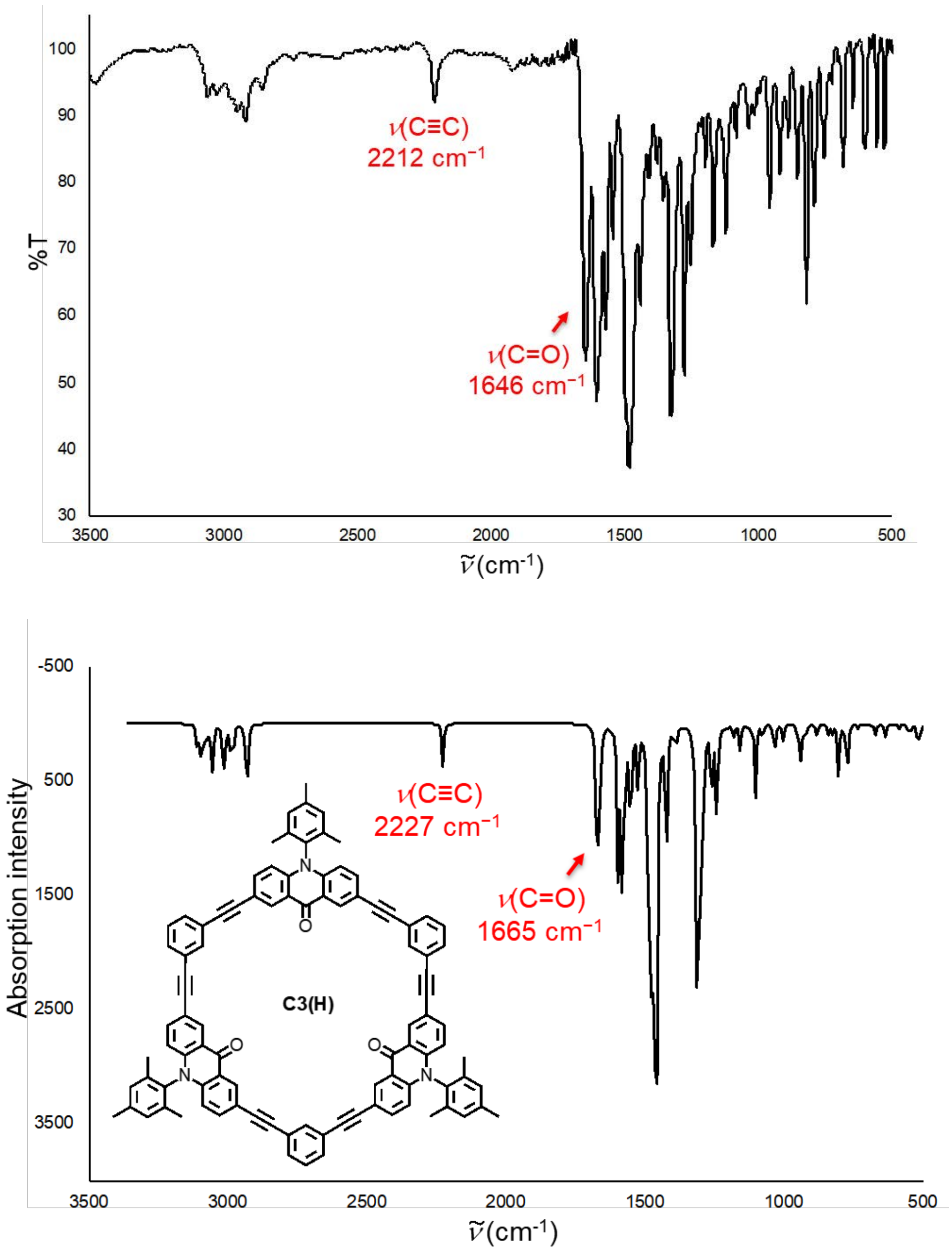

Figure S8. Observed (top) and calculated (bottom) IR spectra of **C3(H)**. The calculated spectrum was obtained by the frequency analysis at the B3LYP/6-31G(d) level with the structure optimized at the same level. The calculated wavenumbers were corrected by the scale factor (0.9614) according to the conventional vibrational scaling factors. [S6]

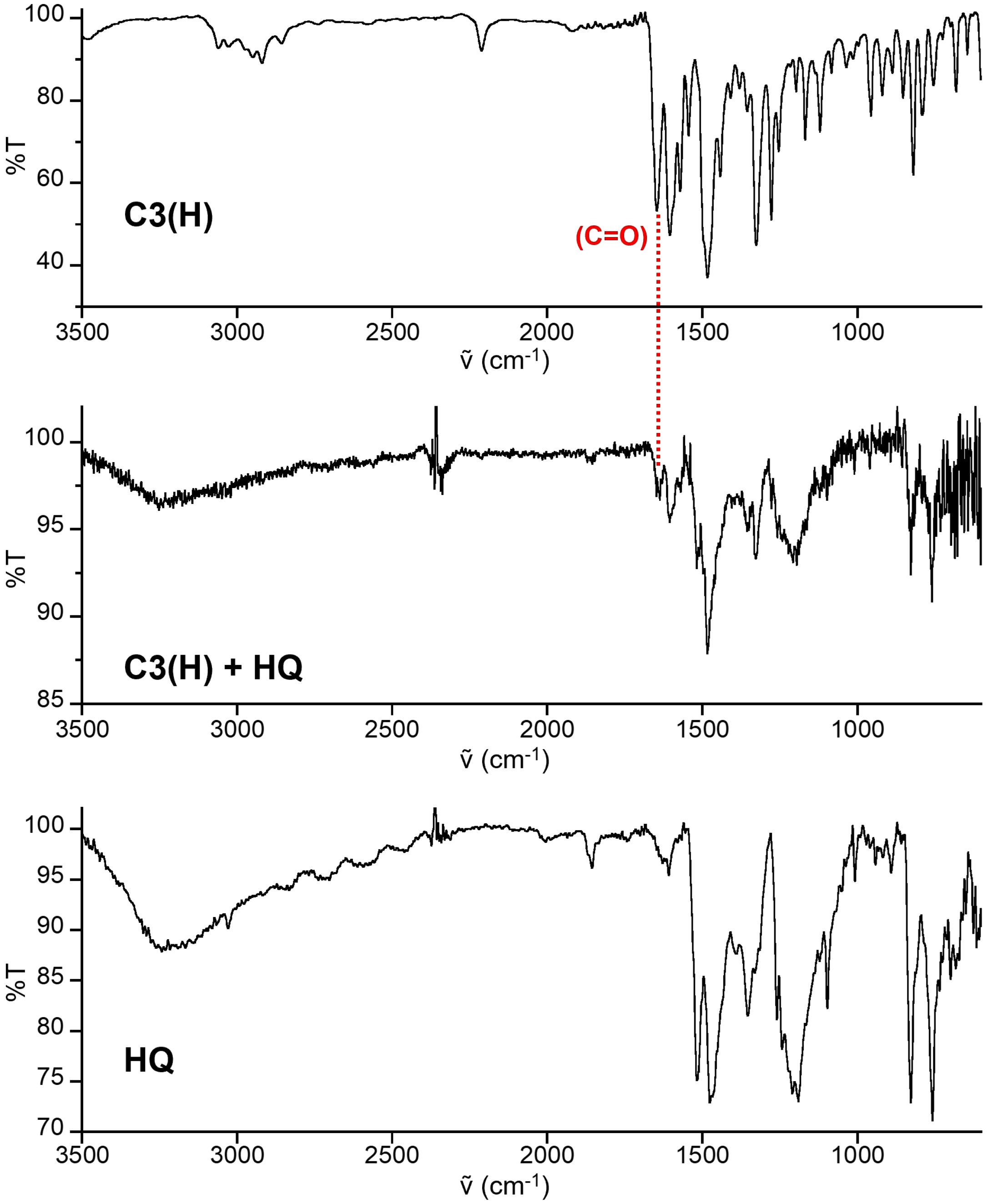

Figure S8-2. IR spectra of **C3(H)** (top), **HQ** (bottom), and a mixture of **C3(H)** and **HQ** (middle). The mixture was obtained by recrystallization from an acetone solution of **C3(H)** and **HQ** (ca. 1:5). The spectrum of the mixture is mostly a superposition of those of **C3(H)** and **HQ**.

4. X-ray Crystallography

Diffraction data of **C3(H)** and **C4(Mes)** were collected at –150 °C on a Rigaku XtaLAB Synergy R/DW HyPix diffractometer with multi-layer mirror monochromated Cu Kα radiation (λ = 1.54184 Å, T = 123 K). Diffraction data of **C3(Tip)** were collected on a diffractometer equipped in a beamline BL-5A at the High Energy Accelerator Research Organization (KEK), Japan with a Pilatus3 S6M detector (synchrotron, λ = 0.7500 Å, T = 95 K). For the three diffraction data, equivalent reflections were merged, and the images were processed with CrysAlisPro1.171 (Rigaku Oxford Diffraction, 2018). The structures were solved by the intrinsic phasing methods (ShelXT-2015)[S7] for **C3(H)** and **C4(Mes)**, and by the direct methods (ShelXS-2015)[S7] for **C3(Tip)**, and all the structures were refined by full-matrix least squares calculations on F^2 (ShelXL-2018)[S7] using the Olex2.1.2 (2009) package as a graphical user interface. All non-hydrogen atoms were refined with anisotropic displacement parameters. All hydrogen atoms were created with ideal geometry and refined using a riding model. Since the crystalline solvents were severely disordered in the large inner cavities of the macrocyclic compounds, the solvent mask was applied with PLATON/SQUEEZE[S7] to the cavities where the solvent could not be assigned. Therefore, large solvent accessible voids remained in the structures of **C4(Mes)** and **C3(Tip)**, resulting in their low calculated crystal densities. Crystallographic data have been deposited with Cambridge Crystallographic Data Centre:

C3(H) (CCDC 2171120): A yellow prism single crystal was obtained by recrystallization from a 1,1,2,2-tetrachloroethane/CH_3CN solution. Crystallographic data: formula $C_{96}H_{63}N_3O_3$• 3($C_2H_2Cl_4$), FW 1810.00, trigonal, $R\bar{3}c$, a = 29.6402(5), b = 29.6402(5), c = 39.6739(9) Å, V = 30186(12) Å^3, Z = 12, D_c = 1.195 g cm^{-3}, $2\theta_{max}$ = 136.5°, unique reflections used for refinement 6132, R_1 = 0.1097 [$I > 2.0\sigma(I)$], wR_2 = 0.3858 (all data), GOF = 1.659.

C4(Mes) (CCDC 2171123): Yellow prism single crystals were obtained by recrystallization from a THF solution. Diffractions were so weak due to severe disorder of solvent molecules in the large central cavity that only low resolution analysis was carried out. Crystallographic data: formula $C_{164}H_{124}N_4O_4$, FW 2214.66, triclinic, $P\bar{1}$, a = 11.5116(4), b = 21.2367(7), c = 21.6437(7) Å, α = 84.411(3)°, β = 76.895(3)°, γ = 83.972(3)°, V = 5110.1(3) Å^3, Z = 1, D_c = 0.720 g cm^{-3}, $2\theta_{max}$ = 100.9°, unique reflections used for refinement 10648, R_1 = 0.0988 [$I > 2.0\sigma(I)$], wR_2 = 0.3239 (all data), GOF = 1.074.

C3(Tip) (CCDC 2171121): A yellow prism single crystal was obtained by recrystallization from a chloroform/MeOH solution. Crystallographic data: formula $C_{141}H_{129}N_3O_3$, FW 1913.45, trigonal, $P31c$, a = 33.685(2), b = 33.685(2), c = 20.655(5) Å, V = 20297(5) Å^3, Z = 4, D_c =

0.626 g cm^{-3}, 2θ_{max} = 71.4°, unique reflections used for refinement 21734, R_1 = 0.0647 [I > 2.0σ(I)], wR_2 = 0.2203 (all data), GOF = 0.947. X-ray structures of this compound are given in Figure S9.

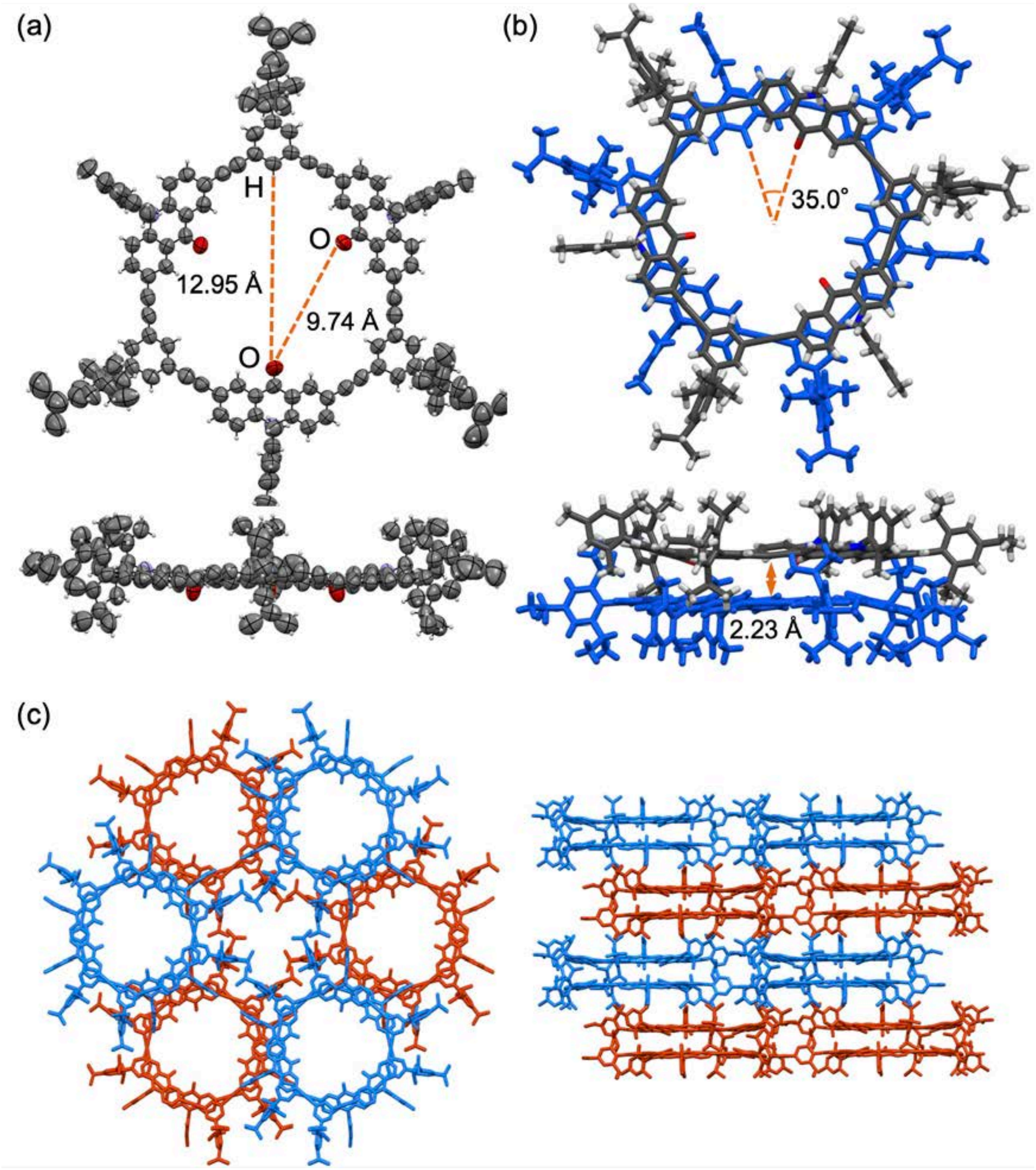

Figure S9. X-ray structures of **C3(Tip)**. Solvent molecules are omitted for clarity. (a) ORTEP drawings of a single molecule. (b) Structures of a dimeric pair. (c) Packing diagrams.

5. Computational Chemistry

DFT calculations were carried out with Gaussian16 program. [S8] The structures were optimized at the B3LYP/6-31G(d) level. The frequency analysis was carried out for each optimized structure, giving no imaginary wavenumber. The excited states were calculated by the time-dependent (TD)-DFT method (**C3(H)**: NSTATE = 50, **5**: NSTATE = 20). The calculated spectra and selected data are shown in Figures S10 and S11 and Tables S2 and S3. The electrostatic potential map of **C3(H)** was simulated by GaussView 6.1.1 [S9] from the output data.

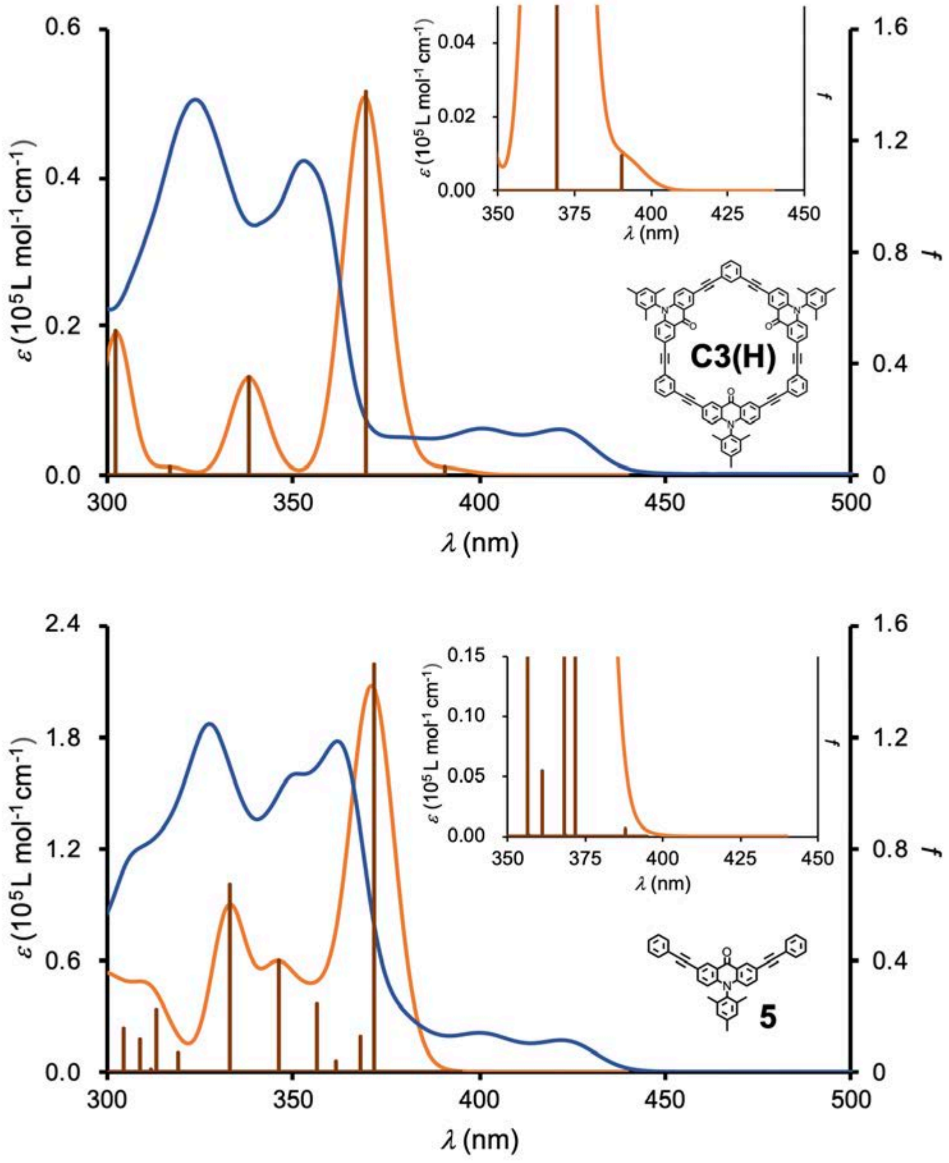

Figure S10. Calculated UV-vis spectra (orange lines) with oscillator strengths (brown bars) at TD-B3LYP/6-31G(d)//B3LYP/6-31G(d) level and observed spectra (blue lines) of **C3(H)** (top) and **5** (bottom). Half band width was set to 500 cm^{-1} in the calculated spectra.

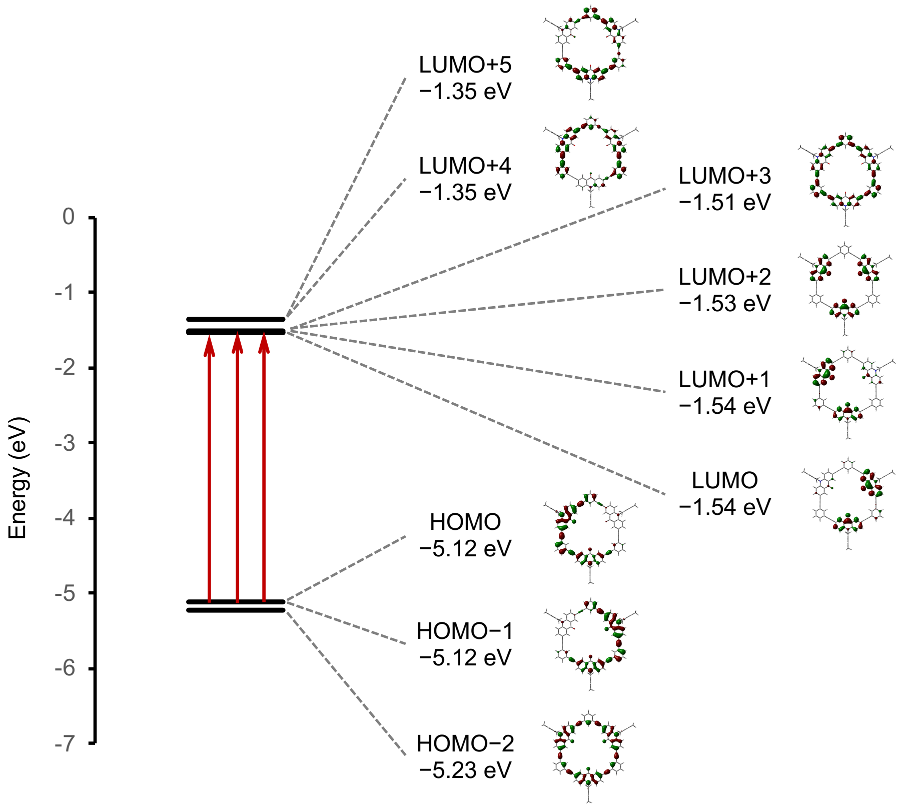

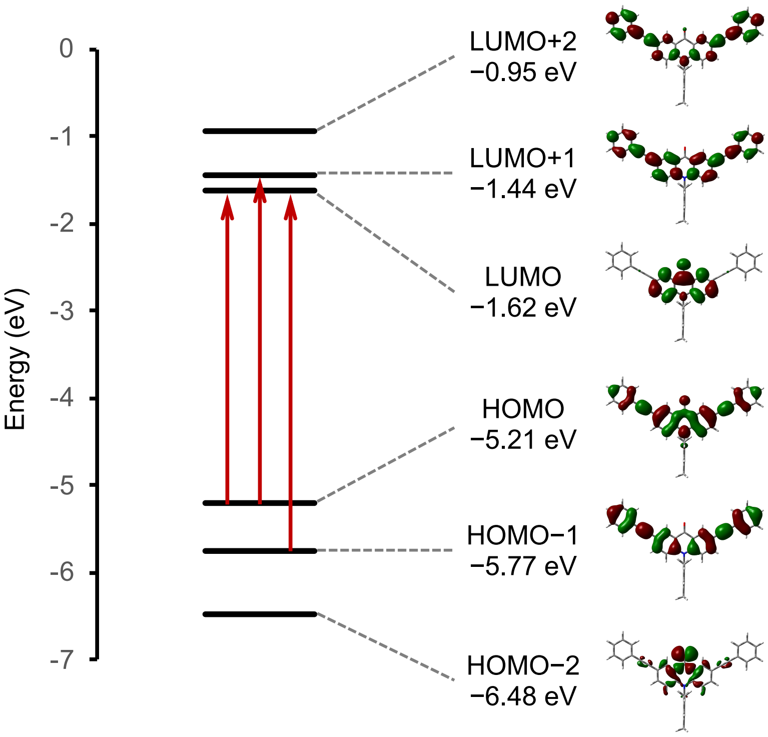

Figure S11. Kohn-Sham orbitals and orbital energy diagrams of **C3(H)** (top) and **5** (bottom) calculated at TD-B3LYP/6-31G(d)//B3LYP/6-31G(d) level. Selected excitation data and assignments are shown.

Table S2. Selected excitation data and assignments for the absorption of **C3(H)** calculated by TD-DFT method at TD-B3LYP/6-31G(d)//B3LYP/6-31G(d) level.

State	λ [nm] [a]	*f* [b]	Transition assignment [c]	
1	394.9	0.0000	H–2 → L+3	42.2%
			H–1 → L+5	24.1%
			H → L+4	24.1%
2	388.1	0.0039	H–2 → L+2	34.3%
			H–1 → L+2	15.9%
			H → L	15.4%
			H–2 → L+1	14.3%
3	388.1	0.0039	H–1 → L+2	32.9%
			H–1 → L	16.0%
			H → L+1	15.7%
			H–2 → L	14.4%
4	387.0	0.0000	H–1→ L	40.7%
			H → L+1	40.6%
			H–2 → L+2	14.4%
5	371.7	1.4615	H → L+3	77.6%
			H–2 → L+4	7.7%
6	371.6	1.4614	H–1 → L+3	77.5%
			H–2 → L+5	7.7%
7	368.6	0.0005	H → L	51.3%
			H–1 → L+1	42.6%
8	368.3	0.1288	H → L+2	45.1%
			H → L+1	15.6%
			H–1 → L	15.2%
			H–1 → L+1	11.6%
			H → L	8.4%
9	368.2	0.1260	H–1 → L+2	46.9%
			H–1 → L+1	17.1%
			H → L	12.7%
			H → L+1	9.9%
			H–1 → L	9.4%
10	361.2	0.0362	H–2 → L	76.5%
			H–1 → L+2	6.7%

[a] Excitation wavelength. [b] Oscillator strength. [c] H: HOMO, L: LUMO.

Table S3. Selected excitation data and assignments for the absorption of **5** calculated by TD-DFT method at TD-B3LYP/6-31G(d)//B3LYP/6-31G(d) level.

State	λ [nm] [a]	f [b]	Transition assignment [c]	
1	390.5	0.0253	H → L	
2	369.3	1.3760	H → L+1	
3	347.6	0.0000	H–2 → L	
4	338.1	0.3550	H–1→ L	
5	316.7	0.0287	H → L+2	70.5%
			H–1 → L+1	28.2%

[a] Excitation wavelength. [b] Oscillator strength. [c] H: HOMO, L: LUMO.

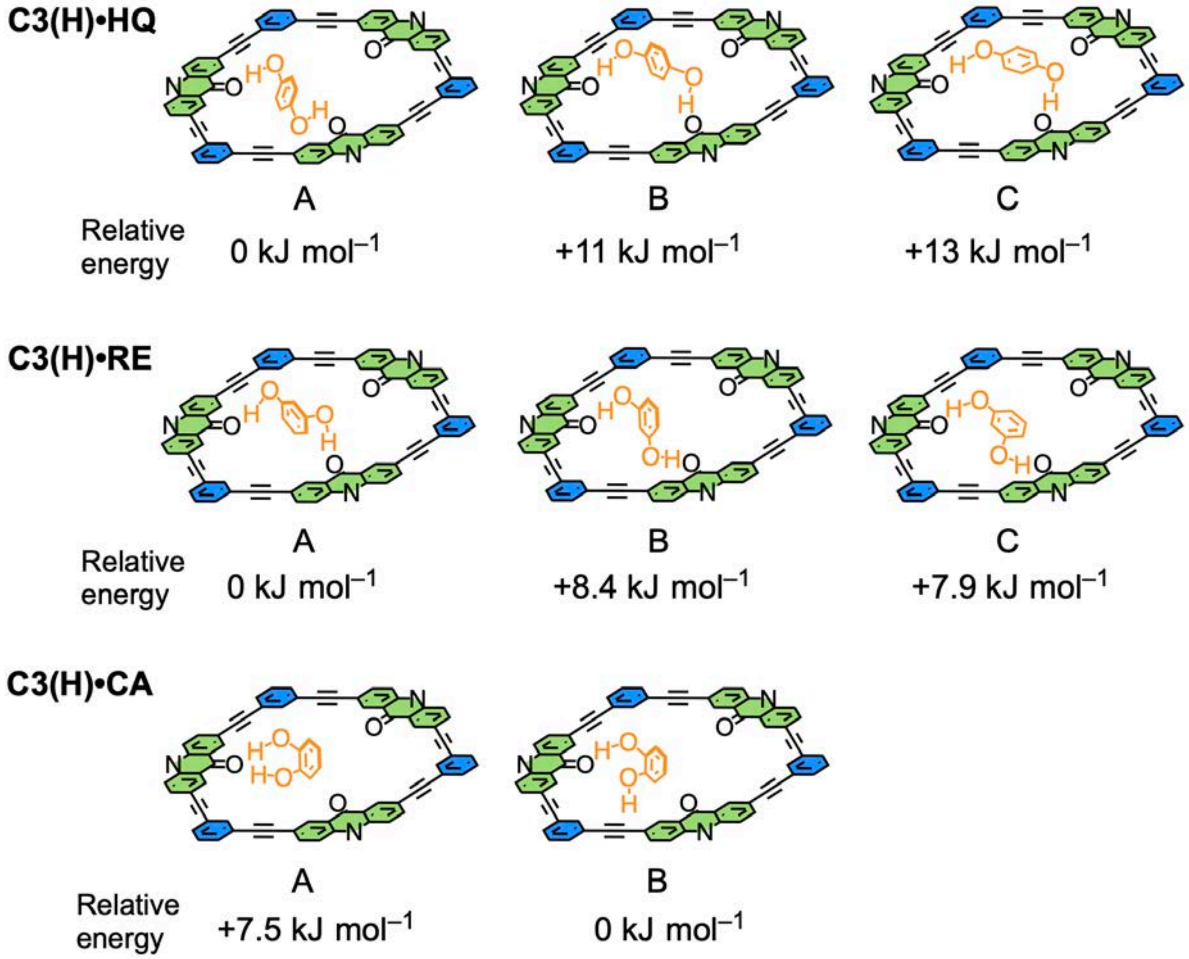

Figure S12. Schematic presentations of possible structures of host-guest 1:1 complexes, **C3(H)•HQ**, **C3(H)•RE**, and **C3(H)•CA** obtained by the conformational search by using CONFLEX program with the MMFF94s force field. In each complex, structures within 20 kJ mol^{-1} relative to the most stable structure at 298 K are given. Mesityl groups are omitted for clarity.

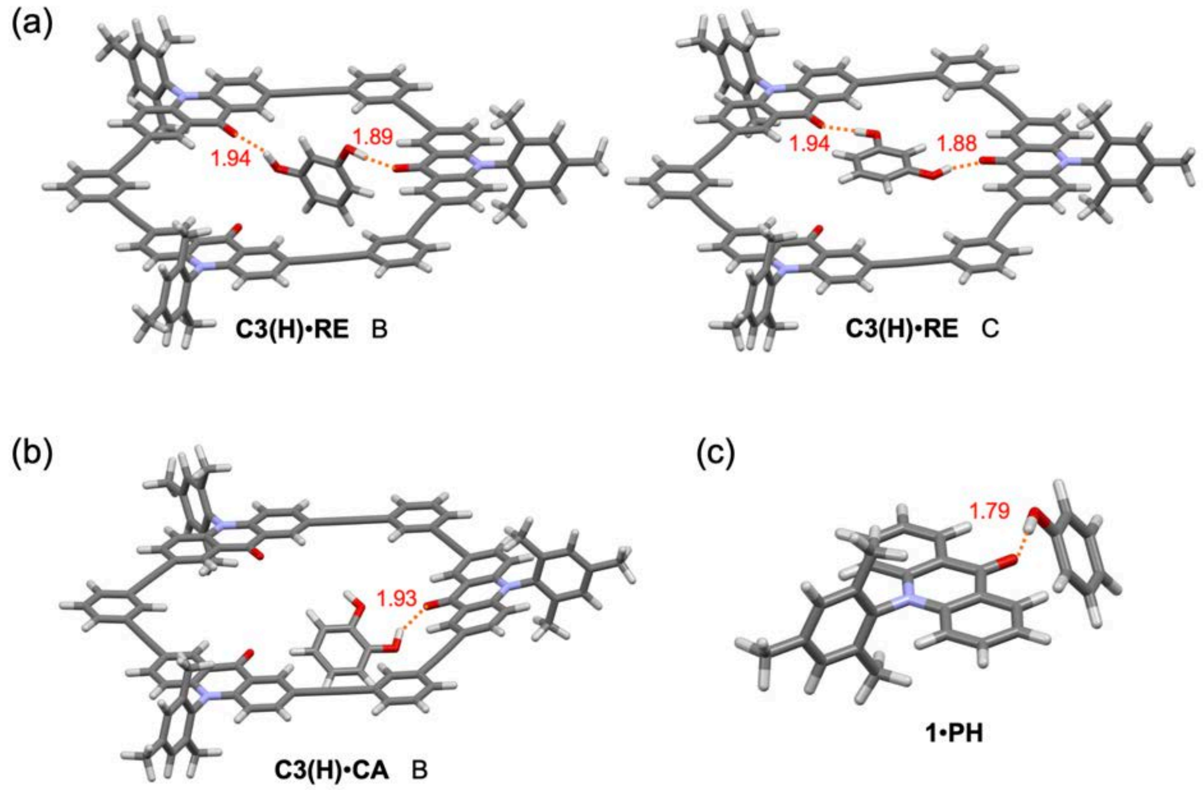

Figure S13. Optimized structures of (a) **C3(H)•RE** B, (b) **C3(H)•RE** C, (c) **C3(H)•CA** B, and (c) **1•PH** 1:1 complexes calculated at B3LYP/6-31G(d) level. Red values in the structures are the distances of OH···O=C bonds in Å. See also Figure 8 and Table 2 in the main part.

Table S4. Calculated thermodynamic data of host **C3(H)** and guests [phenol (**PH**), hydroquinone (**HQ**), resorcinol (**RE**), and catechol (**CA**)] at B3LYP/6-31G(d) level.

	ΔE [a.u.] [a]	ΔH_{298} [a.u.] [a]	ΔG_{298} [a.u.] [a]
**C3(H)**	–4085.959096	–4084.574355	–4084.804326
**HQ**	–382.678132	–382.561811	–382.600088
**C3(H)•HQ** A	–4468.665317	–4467.161143	–4467.410477
**C3(H)•HQ** A host [b]	–4085.957907	–	–
**C3(H)•HQ** A guest [c]	–382.677207	–	–
**C3(H)•HQ** B	–4468.662738	–4467.158950	–4467.407218
**C3(H)•HQ** C	–4468.661441	–4467.157509	–4467.408040
**RE**	–382.681627	–382.565144	–382.603163
**C3(H)•RE** A	–4468.671182	–4467.166783	–4467.413530
**C3(H)•RE** A host [b]	–4085.957784	–	–
**C3(H)•RE** A guest [c]	–382.680771	–	–
**C3(H)•RE** B	–4468.666138	–4467.161979	–4467.410473
**C3(H)•RE** C	–4468.665408	–4467.161297	–4467.411876
**CA**	–382.681614	–382.565095	–382.603339
**C3(H)•CA** A	–4468.658158	–4467.154366	–4467.403630
**C3(H)•CA** A host [b]	–4085.957914	–	–
**C3(H)•CA** A guest [c]	–382.669027	–	–
**C3(H)•CA** B	–4468.654322	–4467.150782	–4467.401391
**PH**	–307.464861	–307.353624	–307.389042
**C3(H)•PH**	–4393.442257	–4391.943545	–4392.191581
**C3(H)•PH** host [b]	–4085.958372	–	–
**C3(H)•PH** guest [c]	–307.464501	–	–
**1**	–979.809503	–979.436560	–979.507876
**1•PH**	–1287.292869	–1286.805983	–1286.897637

[a] 1 a.u. = 2625.5 kJ mol^{-1} = 27.2116 eV.

[b] Energy for the host moiety only in the optimized complex structure.

[c] Energy for the guest moiety only in the optimized complex structure.

Noncovalent Interaction (NCI) Analysis

The input files (.wfx format) for the optimized structures at the B3LYP/6-31G(d) level were prepared by the Gaussian program. The calculations for the NCI analysis were carried out with NCIPLOT 4.0 program. [S10] The output file (.dat format) were used to generate two dimensional reduced density gradient (RDG) vs. sign(λ_2)ρ plot (Figure S15). The 3D RDG isosurface was visualized by using other output files (grad.cube, dens.cube, and .vmd formats) with VMD program. [S11]

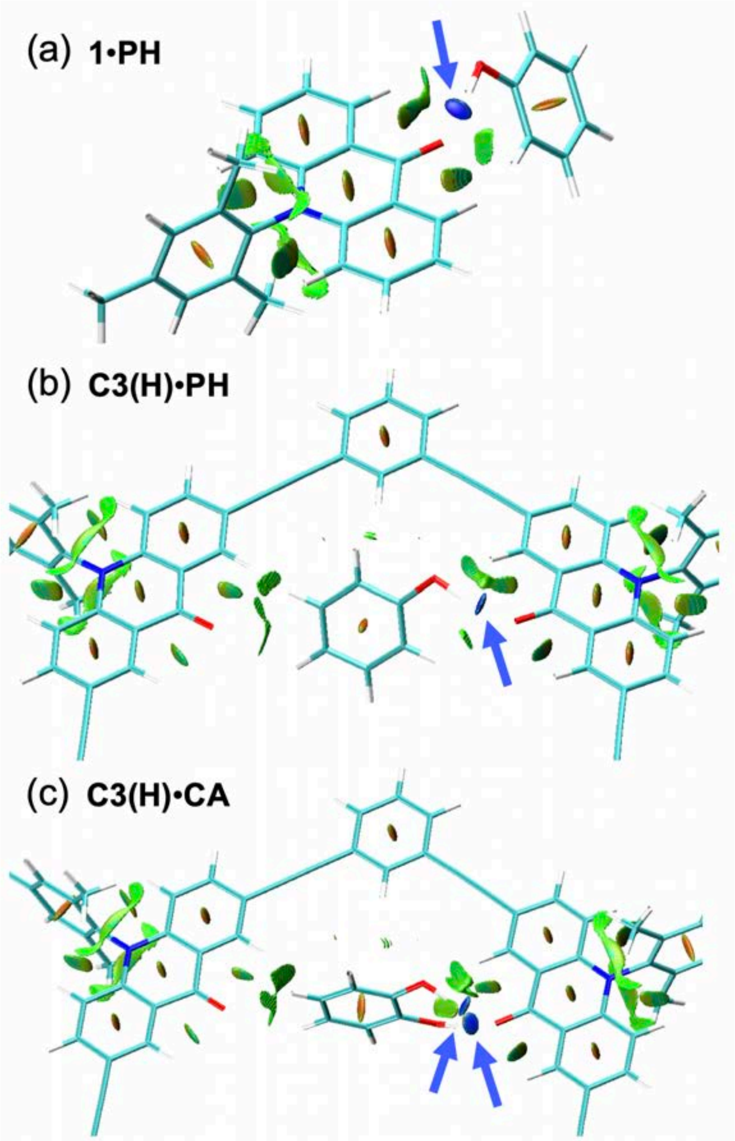

Figure S14. Calculated structures of (a) **1•PH**, (b) **C3(H)•PH**, and (c) **C3(H)•CA** A 1:1 complexes calculated at B3LYP/6-31G(d) level with NCI plots (isosurface value 0.6). The colors of the isosurface are blue (attractive interactions), green (weak vdW interactions), and red (repulsive interactions). Blue arrows indicate isosurfaces for hydrogen bonds. The two dimensional reduced density gradient (RDG) vs. sign(λ_2)ρ plots are shown in Figure S15.

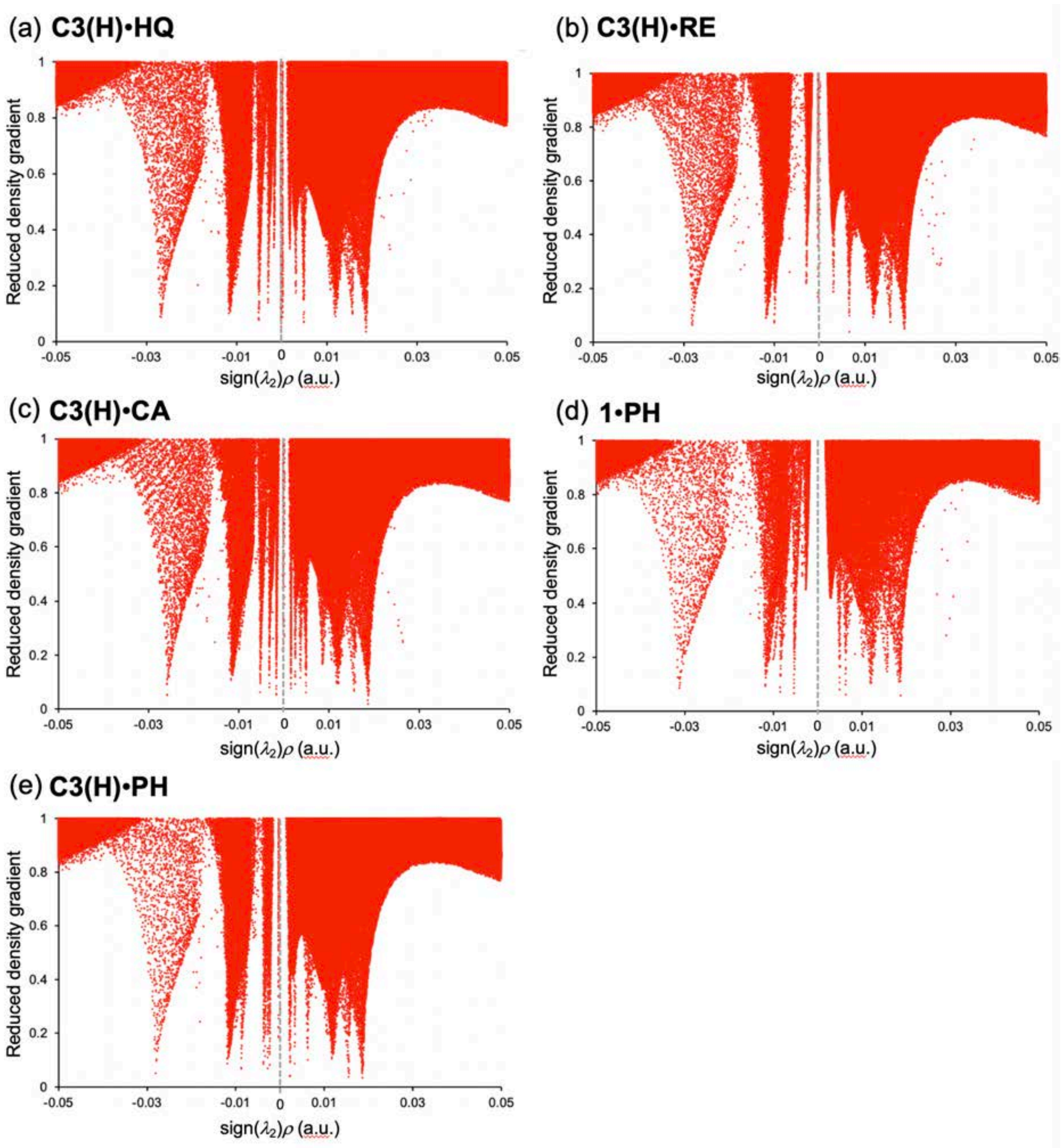

Figure S15. Two dimensional reduced density gradient (RDG) vs. sign(λ_2)ρ plots for the NCI plot analysis of (a) **C3(H)•HQ**, (b) **C3(H)•RE**, (c) **C3(H)•CA** (d) **1•PH**, and (e) **C3(H)•PH** 1:1 complexes. The spikes around –0.03 indicate the presence of hydrogen bonds. The spikes in the range of –0.01 ~ +0.02 indicate the presence of other weak interactions such as van der Waals interactions.

6. NMR Measurements with Guest Molecules

Guest Screening

^{1}H NMR spectra of a mixture of **C3(Mes)** (0.167 mg, 0.128 μmol) and a large excess (10 eq.) of phenolic guest candidates **G1**–**G8** (Figure S16) were measured in $CDCl_3$ (0.50 mL) at 298 K. The chemical shifts of the signals due to the inner protons (1,8-H and 2'-H) were compared with those in the guest-free spectrum. Small chemical shift changes were observed for **G6**, **G7**, and **G8.**

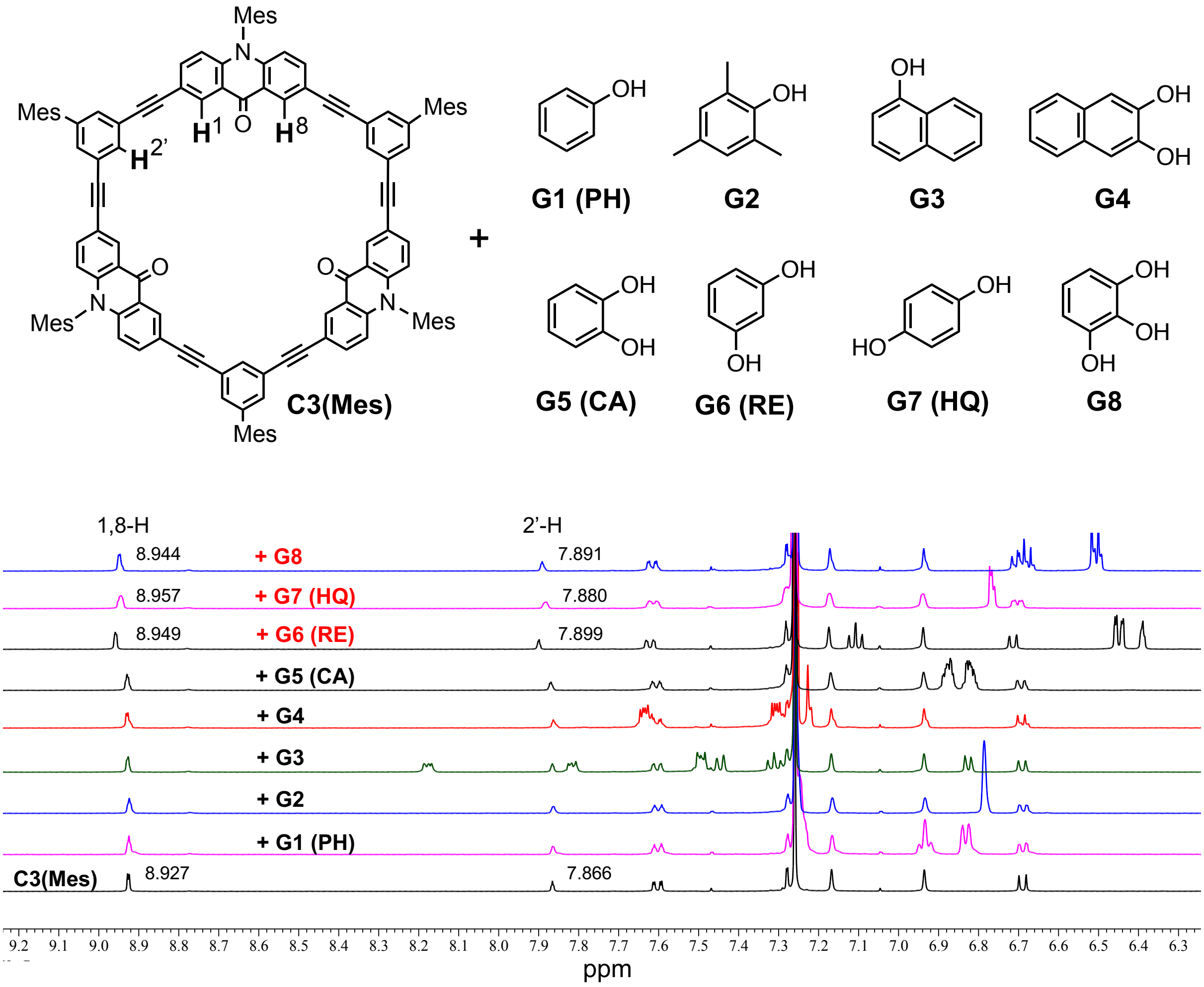

Figure S16. ^{1}H NMR spectra of **C3(Mes)** and a mixture of **C3(Mes)** and phenolic guest candidates **G1**–**G8** in $CDCl_3$ at 298 K. [**C3(Mes)**] = 1.0×10^{-4} mol L^{-1}, [Guest] = 1.0×10^{-3} mol L^{-1}.

We also measured the ^1H NMR of **C3(Mes)** with the following guest candidates in $CDCl_3$. No or very small spectral changes were observed. For some guests, the compounds were insoluble in the NMR solvent.

No or very weak interactions

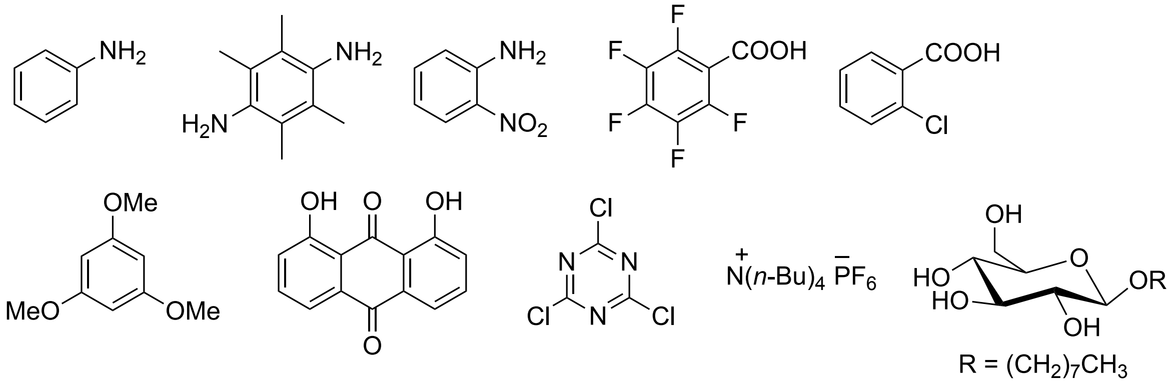

Insoluble

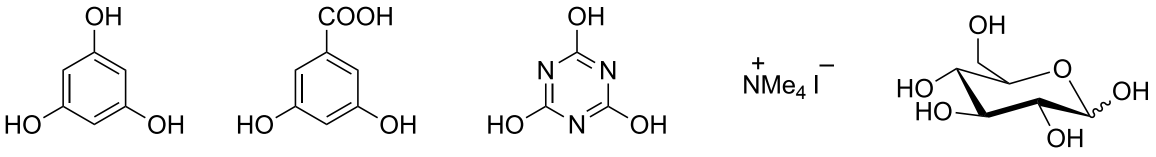

Quantitative measurements

Quantitative measurements were performed with **C3(Mes)** as host (**H**) and the following guests (**G**): resorcinol (**RE**), hydroquinone (**HQ**), and catechol (**CA**). A solution of **H** in $CDCl_3$ (1.00×10^{-3} mol L^{-1}, **A**) and a solution of **G** in $CDCl_3$ (1.00×10^{-3} mol L^{-1}, **B**) were prepared. By mixing the two solutions **A** and **B**, a solution of a mixture of **H** and **G** at the constant total concentration C (= 1.00×10^{-3} mol L^{-1}) containing variable molar fractions of **G** (x) was prepared. For **CA** as guest, the total concentration was set at $C = 2.00 \times 10^{-3}$ mol L^{-1}. The total concentration and the molar fractions were expressed as the following equations.

Total concentration: $[\mathbf{H}] + [\mathbf{G}] = C$

Molar fraction of **G**: $[\mathbf{G}]/([\mathbf{H}] + [\mathbf{G}]) = [\mathbf{G}]/C = x$

Molar fraction of **H**: $[\mathbf{H}]/([\mathbf{H}] + [\mathbf{G}]) = [\mathbf{H}]/C = 1 - x$

^{1}H NMR spectra of the mixed solutions were measured at 25 °C. The observed chemical shift was referenced to the signal due to residual $CHCl_3$ at 7.26 ppm. From the observed spectra, chemical shift differences $\Delta\delta$ (= $\delta - \delta_0$), where δ and δ_0 are the chemical shift at $[\mathbf{G}]/C = x$ and 0, respectively, were read for the signals due to the aromatic protons in **G**. The Job's plot was obtained by plotting $\Delta\delta(x)$ versus x. The nonlinear least-square fitting of $\Delta\delta$ and x values to eq. (1) gave association constant K_a for the 1:1 complex formation, where is δ_c is the chemical shift difference of the signal in the complex relative to that in the host-free guest. [S12]

$$\mathbf{H} + \mathbf{G} \underset{}{\overset{K_a}{\rightleftharpoons}} \mathbf{H{\bullet}G} \quad \cdots\cdots (1)$$

The fitting curves afforded association constants K_a. The free energy difference (ΔG) was calculated from the association constant according to the following equation: $\Delta G = -RT\ln K_a$.

The ^{1}H NMR spectra, the Job's plot, and the fitting curve for **C3(Mes)** and **HQ** are shown in Figure 7 in the main text. These data for **C3(Mes)** and **RE** are shown in Figure S17. These data for **C3(Mes)** and **CA** are shown in Figure S18. The ^{1}H NMR spectra for **C3(Mes)** and **PH** are shown in Figure S19.

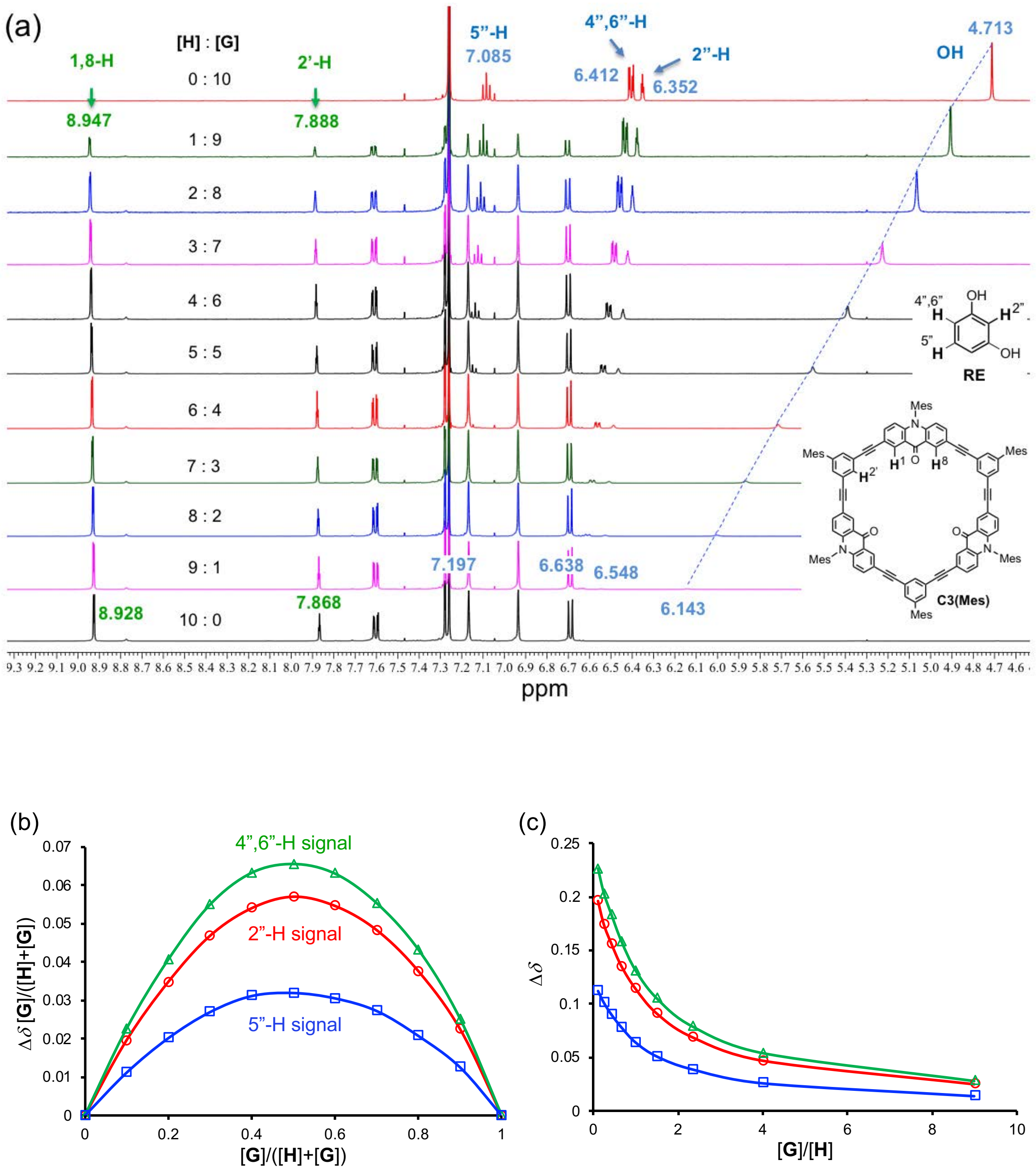

Figure S17. (a) ^{1}H NMR spectra of mixtures of **C3(Mes)** (host: **H**) and resorcinol **RE** (guest: **G**) at various ratios in $CDCl_3$ at 298 K. [**H**] + [**G**] = 1.0×10^{-3} mol L^{-1}. (b) Job's plot. (c) Plot of chemical shift changes $\Delta\delta$ of the aromatic signal of **RE** against [**G**]/[**H**] ratio. The aromatic signal of **RE** (2"-H, 3",5"-H, and 4"-H) was analyzed for (b) and (c). The least-square fitting assuming the following equilibrium, **H** + **G** ⇄ **H•G**, afforded the association constant K_a to be $(9.9 \pm 1.4) \times 10^2$ L mol^{-1} (4,6-H), corresponding to 17.1 kJ mol^{-1} in $-\Delta G_{298}$.

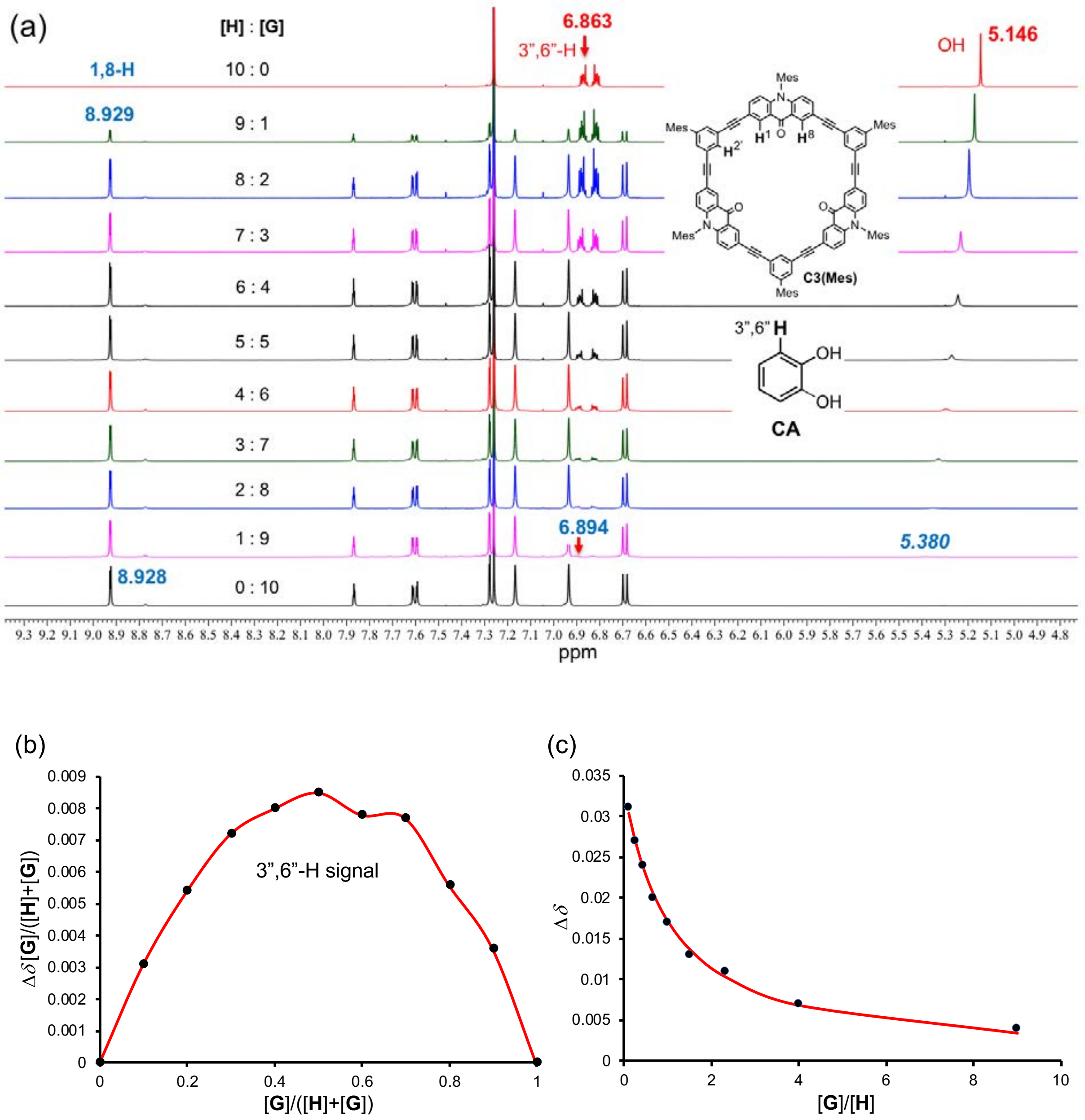

Figure S18. (a) ^{1}H NMR spectra of mixtures of **C3(Mes)** (host: **H**) and catechol **CA** (guest: **G**) at various ratios in $CDCl_3$ at 297 K. [**H**] + [**G**] = 2.0×10^{-3} mol L^{-1}. (b) Job's plot. (c) Plot of chemical shift changes $\Delta\delta$ of the aromatic signal of **CA** against [**G**]/[**H**] ratio. The aromatic signal of **CA** (3",6"-H) was analyzed for (b) and (c). The least-square fitting assuming the following equilibrium, **H** + **G** ⇄ **H•G**, afforded the association constant K_a to be $(2.3 \pm 0.2) \times 10^2$ L mol^{-1}, corresponding to 13.5 kJ mol^{-1} in $-\Delta G_{298}$.

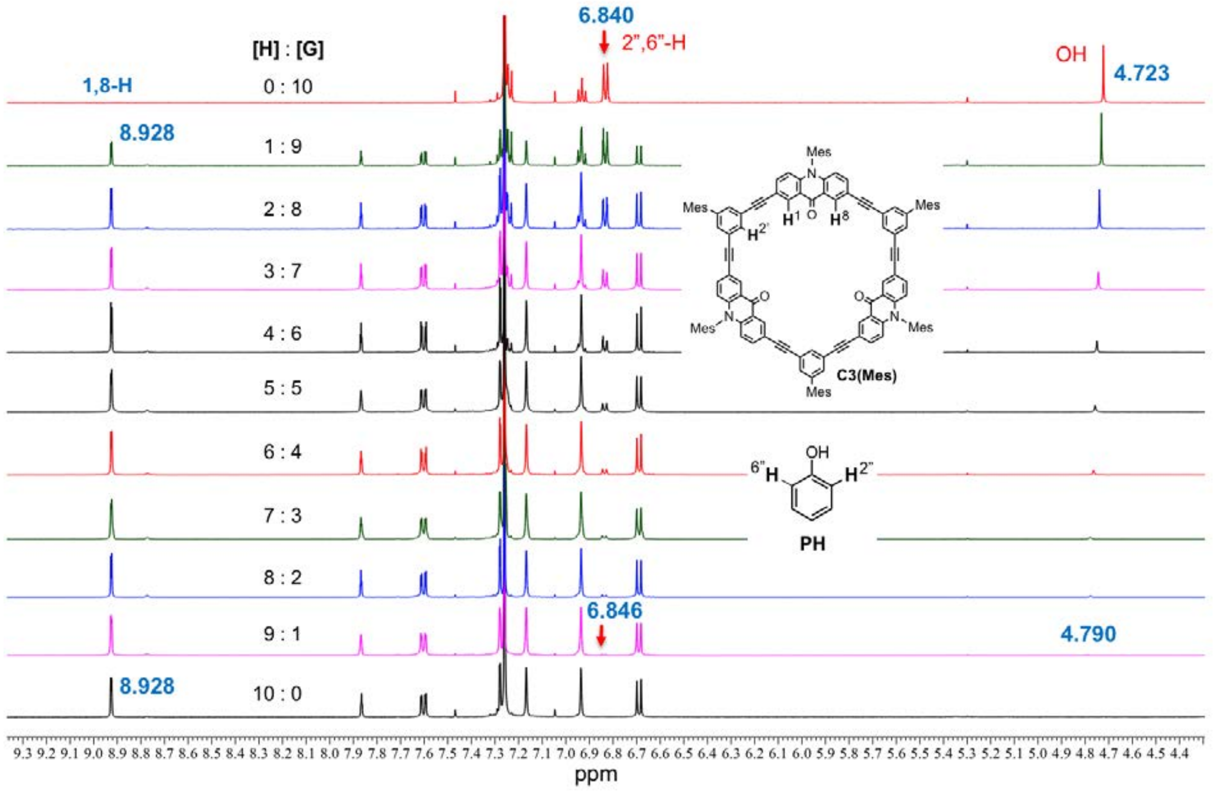

Figure S19. ^{1}H NMR spectra of mixtures of **C3(Mes)** (host: **H**) and phenol **PH** (guest: **G**) at various ratios in $CDCl_3$ at 297 K. [**H**] + [**G**] = 1.0×10^{-3} mol L^{-1}.

7. NMR Charts

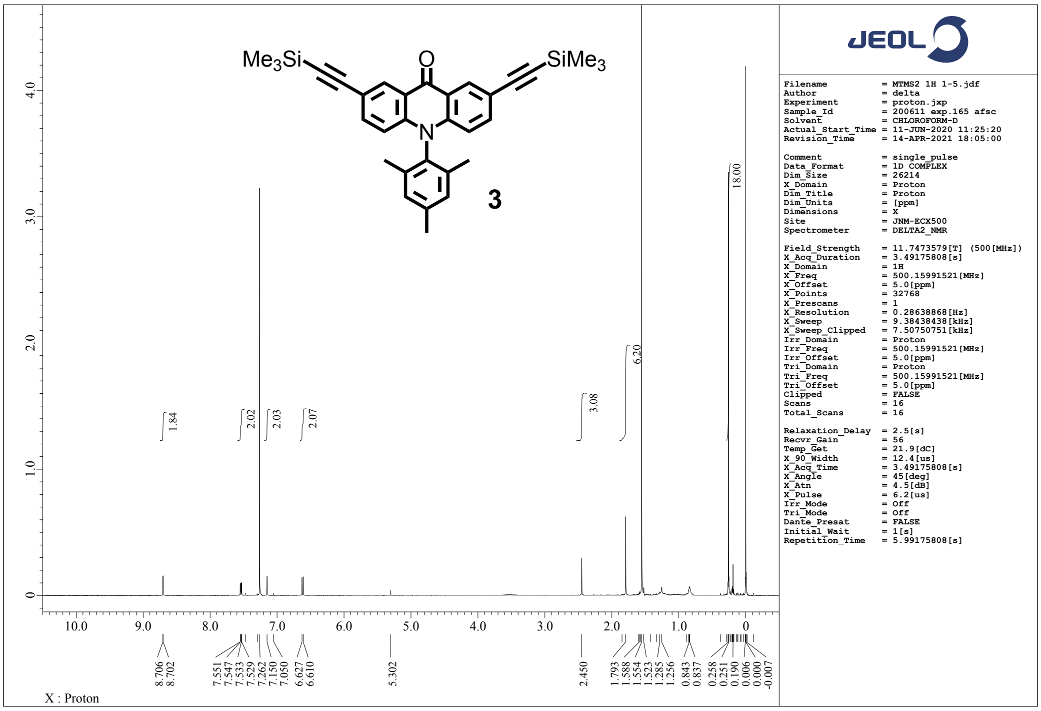

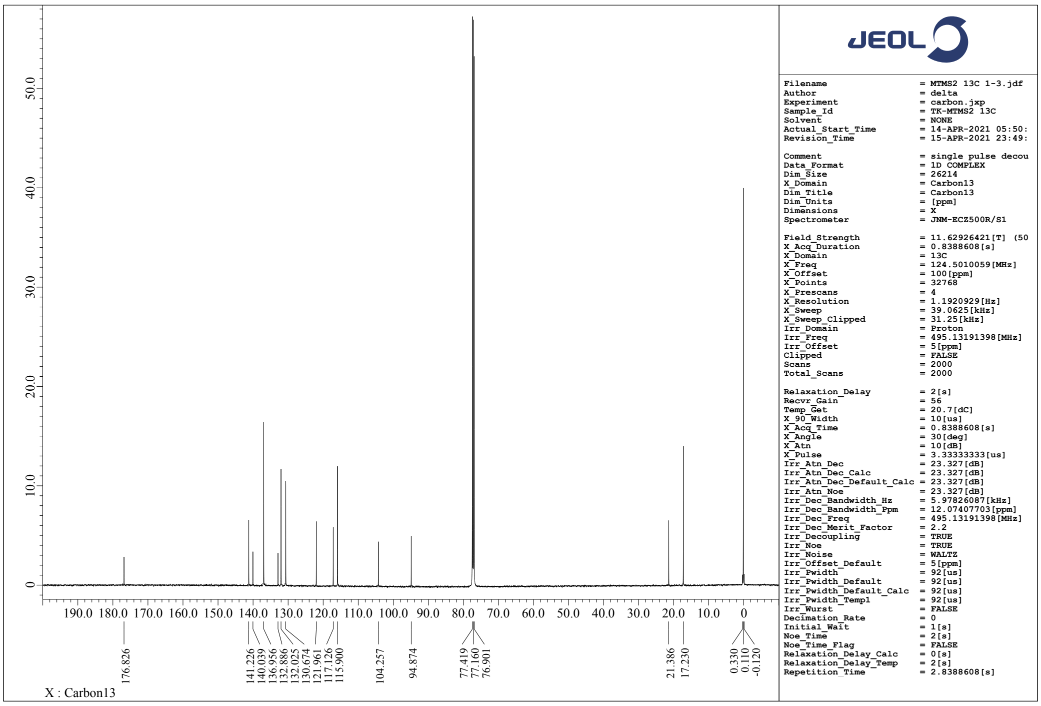

Figure S20. ^{1}H (top, 500 MHz) and ^{13}C NMR spectra (bottom, 125 MHz) of **3** in $CDCl_3$.

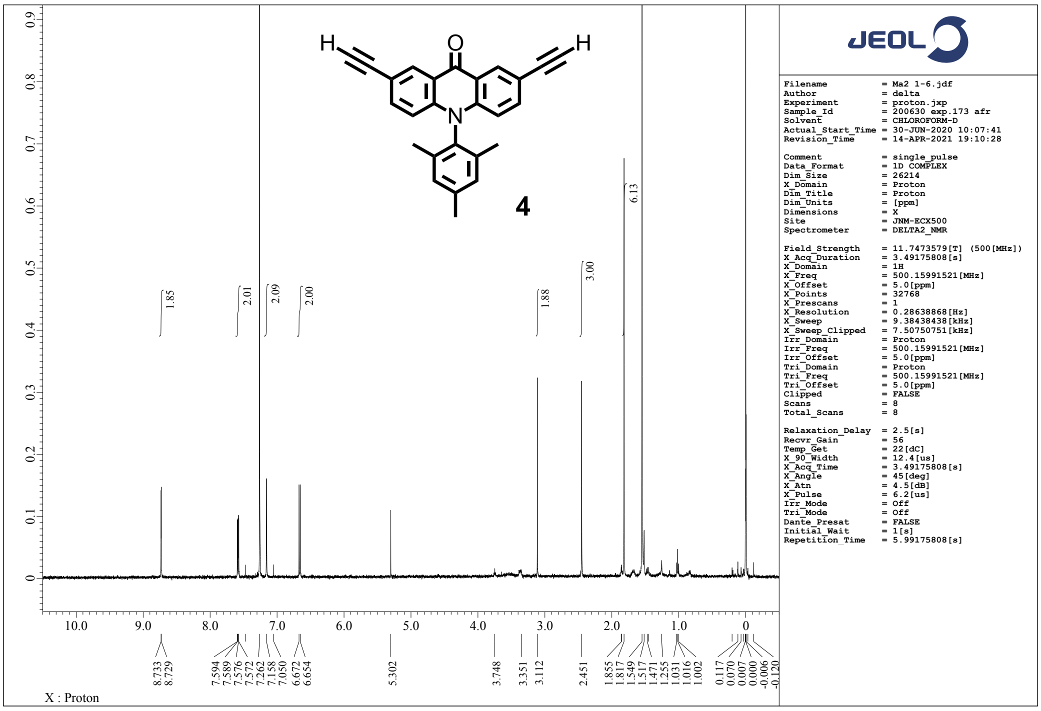

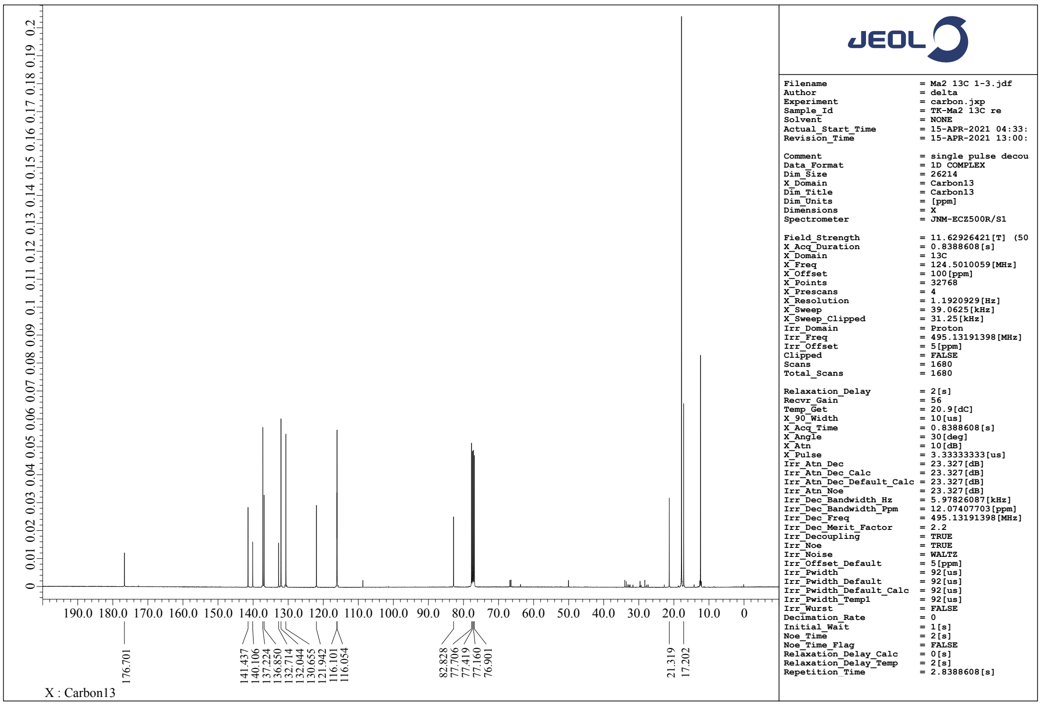

Figure S21. ^{1}H (top, 500 MHz) and ^{13}C NMR spectra (bottom, 125 MHz) of **4** in $CDCl_3$.

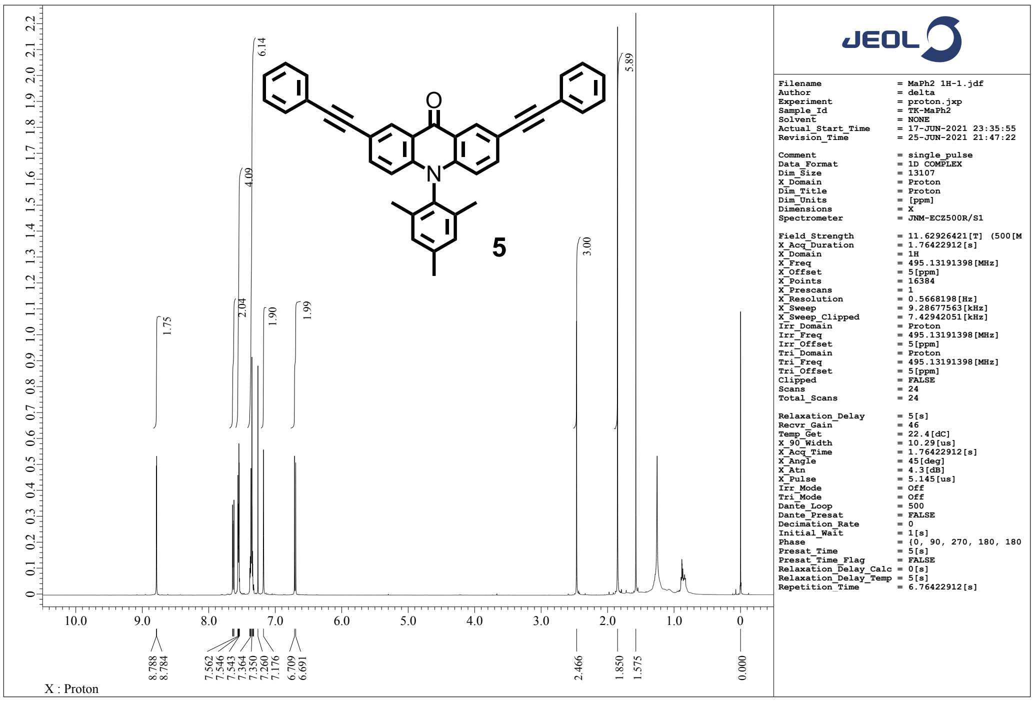

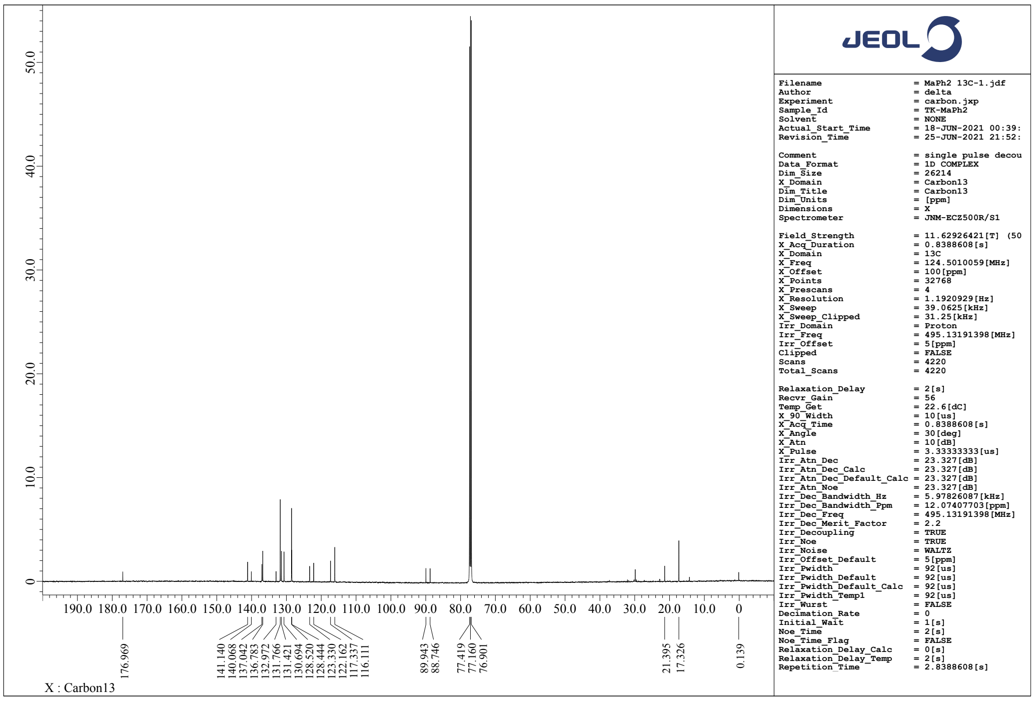

Figure S22. ^{1}H (top, 500 MHz) and ^{13}C NMR spectra (bottom, 125 MHz) of **5** in $CDCl_3$.

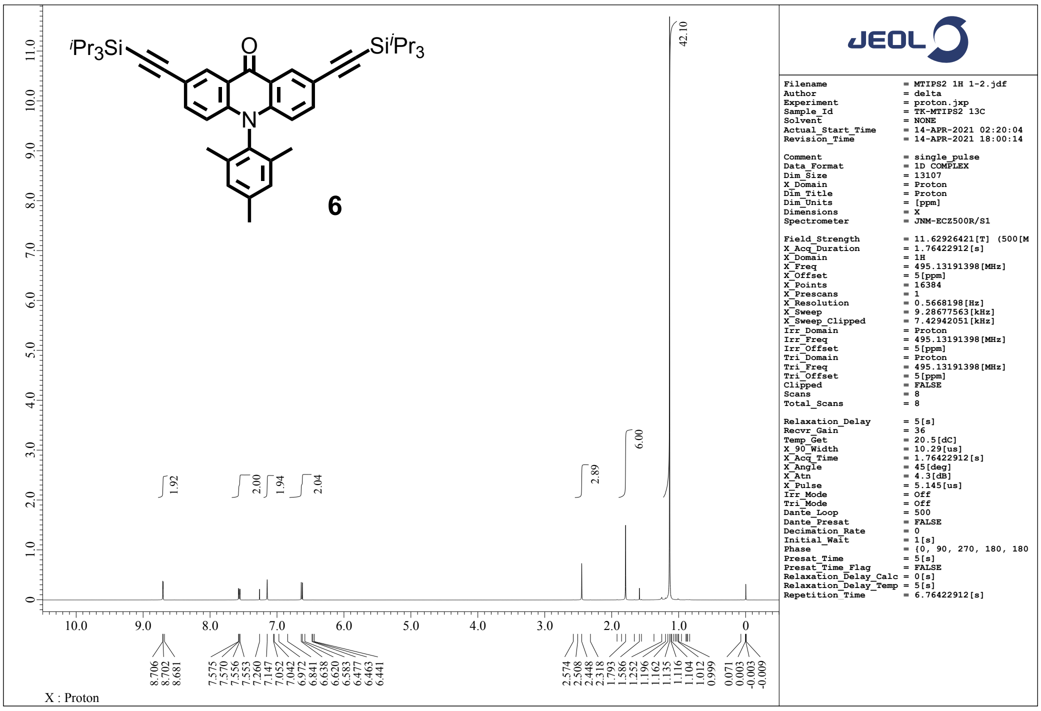

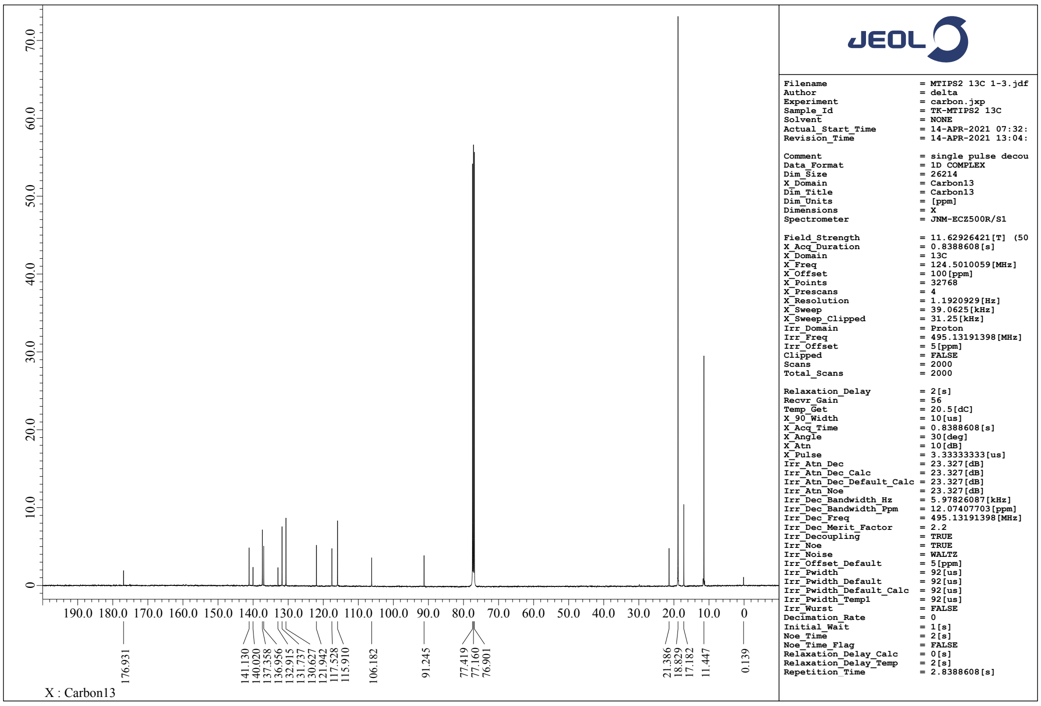

Figure S23. ^{1}H (top, 500 MHz) and ^{13}C NMR spectra (bottom, 125 MHz) of **6** in $CDCl_3$.

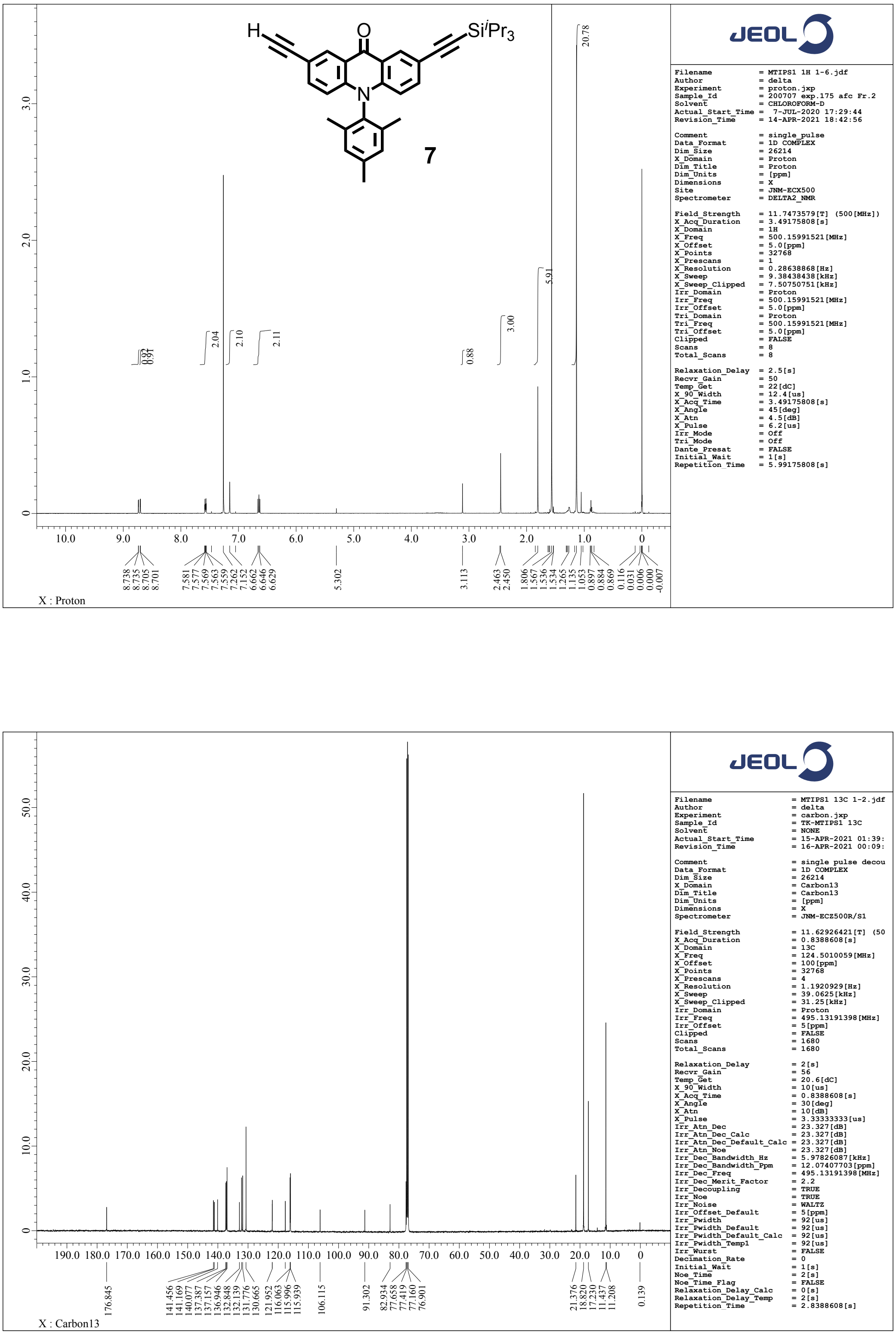

Figure S24. ^{1}H (top, 500 MHz) and ^{13}C NMR spectra (bottom, 125 MHz) of **7** in $CDCl_3$.

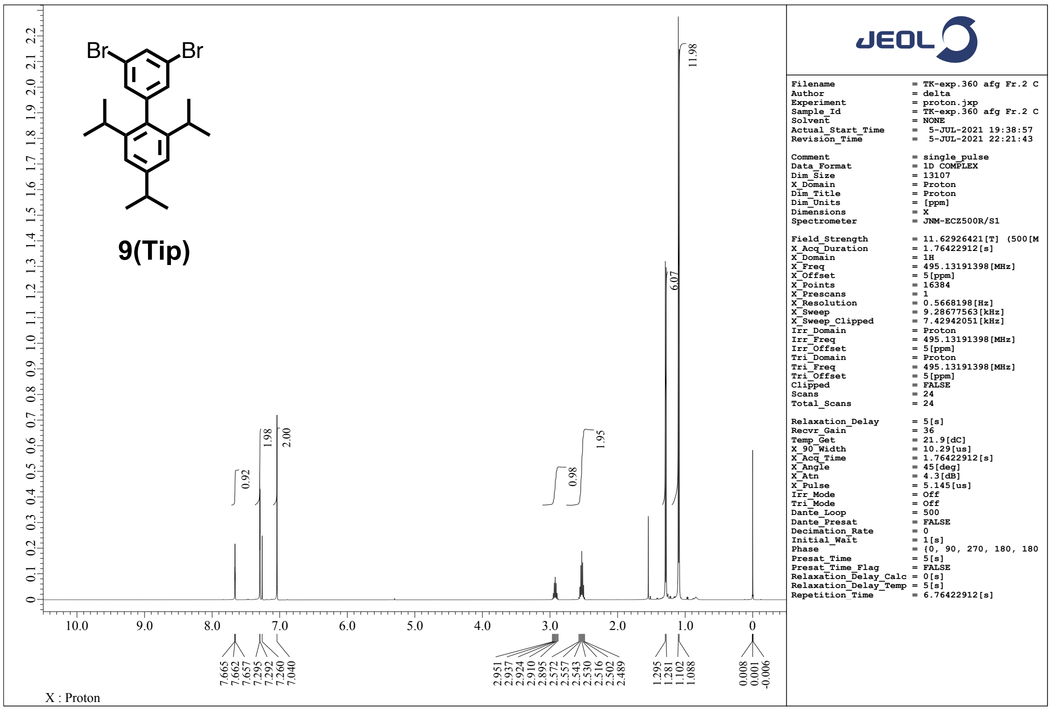

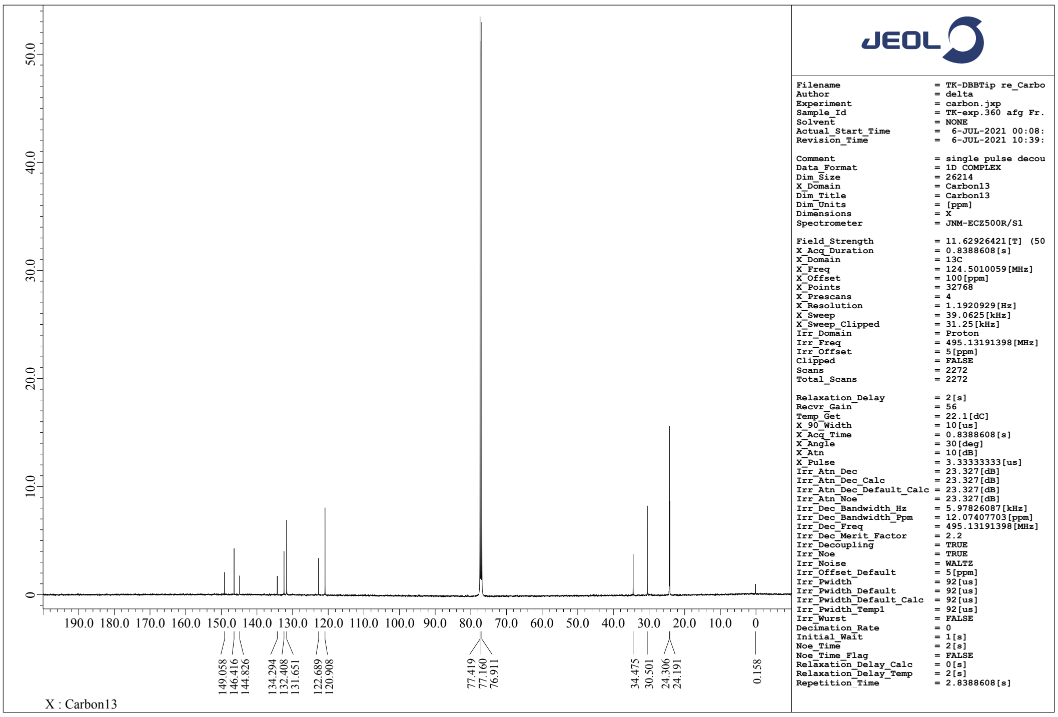

Figure S25. ^{1}H (top, 500 MHz) and ^{13}C NMR spectra (bottom, 125 MHz) of **9(Tip)** in $CDCl_3$.

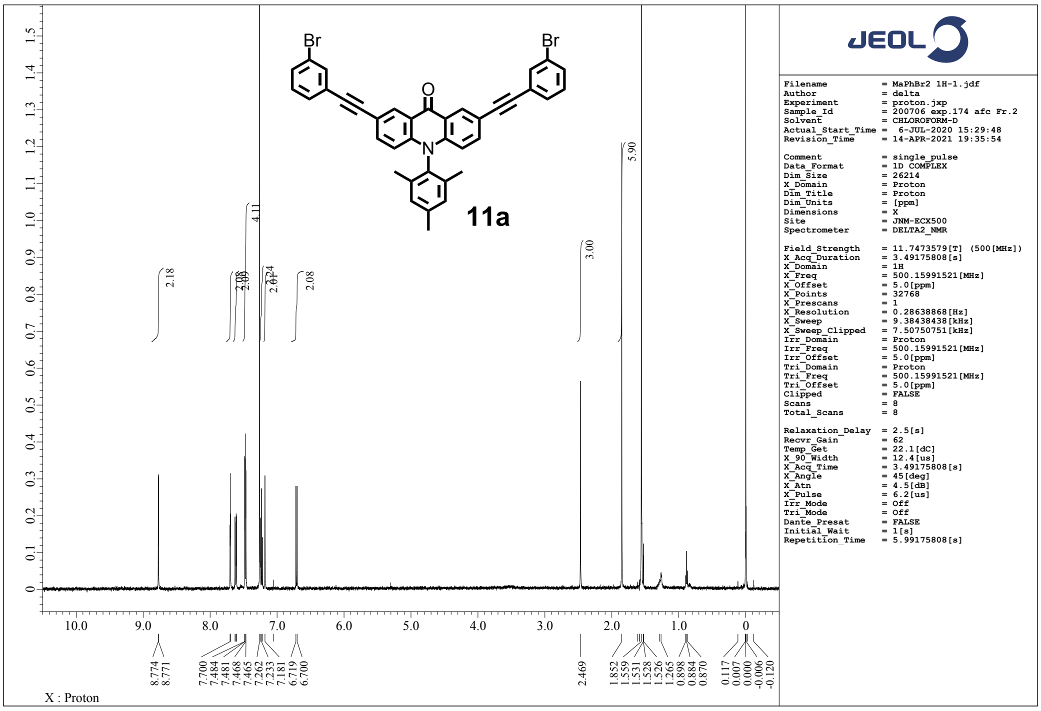

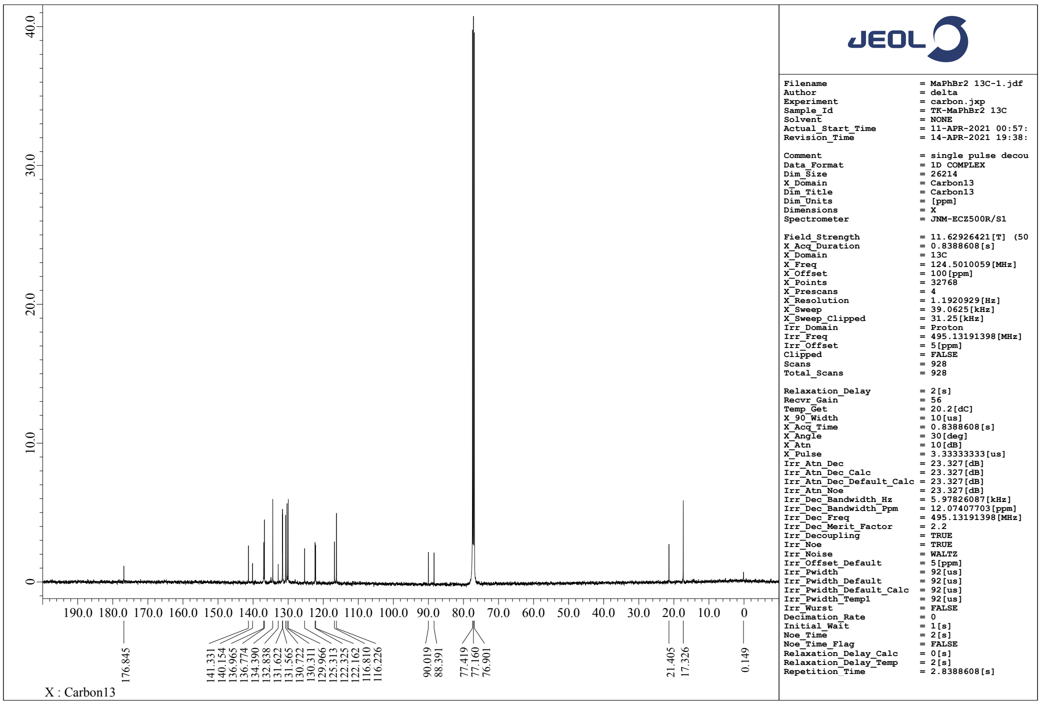

Figure S26. ^{1}H (top, 500 MHz) and ^{13}C NMR spectra (bottom, 125 MHz) of **11a** in $CDCl_3$.

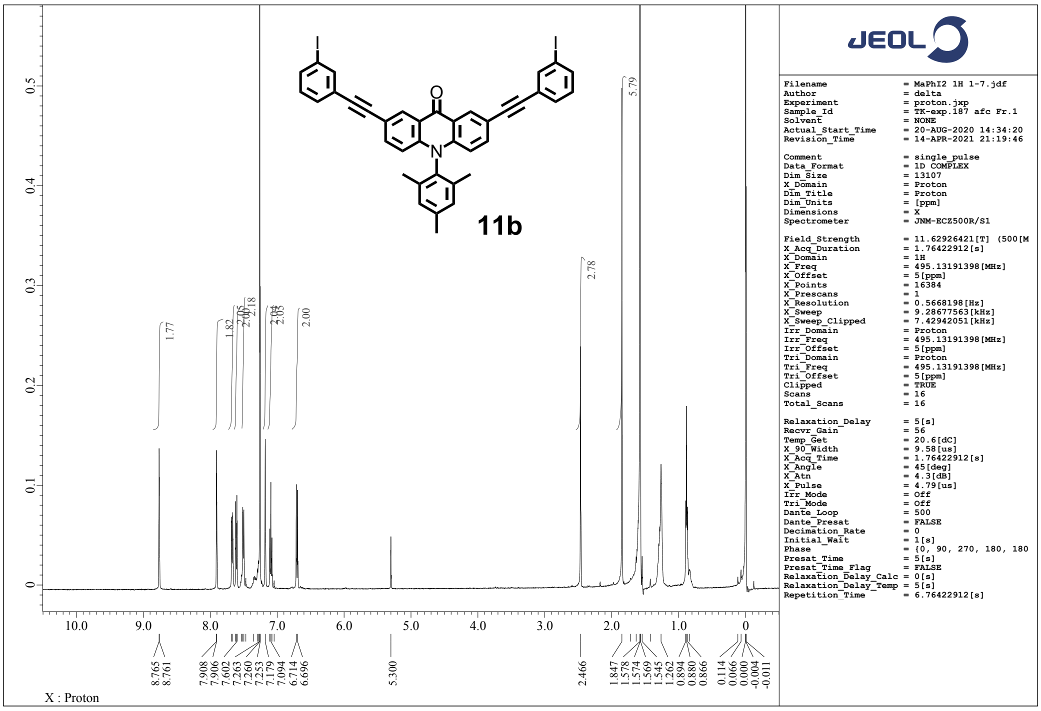

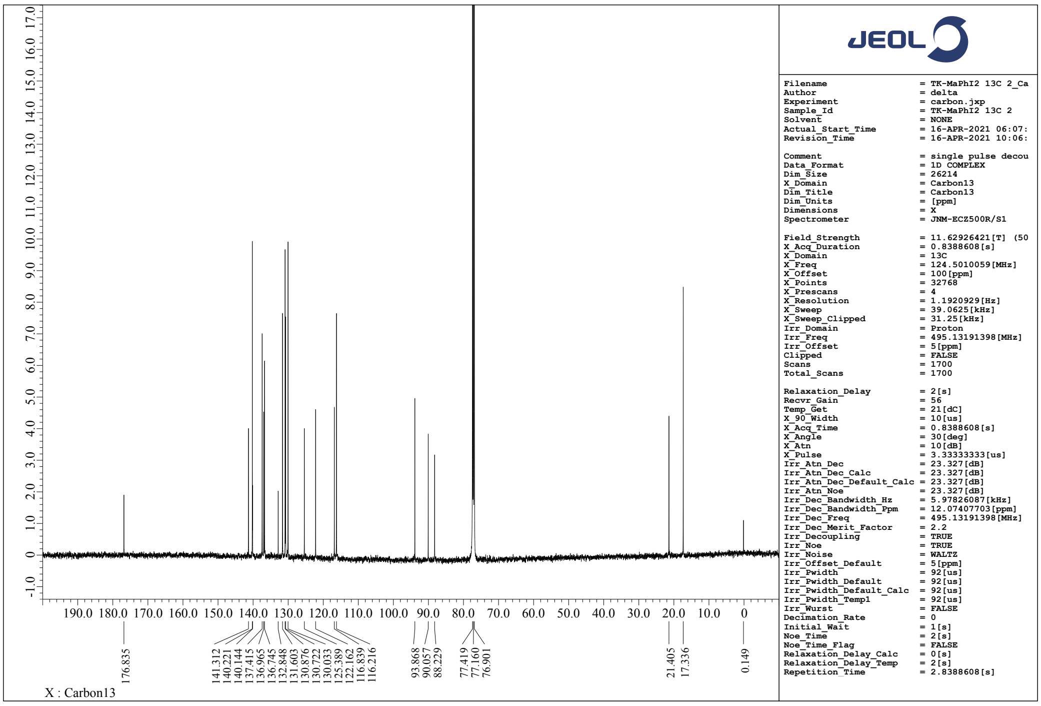

Figure S27. ^{1}H (top, 500 MHz) and ^{13}C NMR spectra (bottom, 125 MHz) of **11b** in $CDCl_3$.

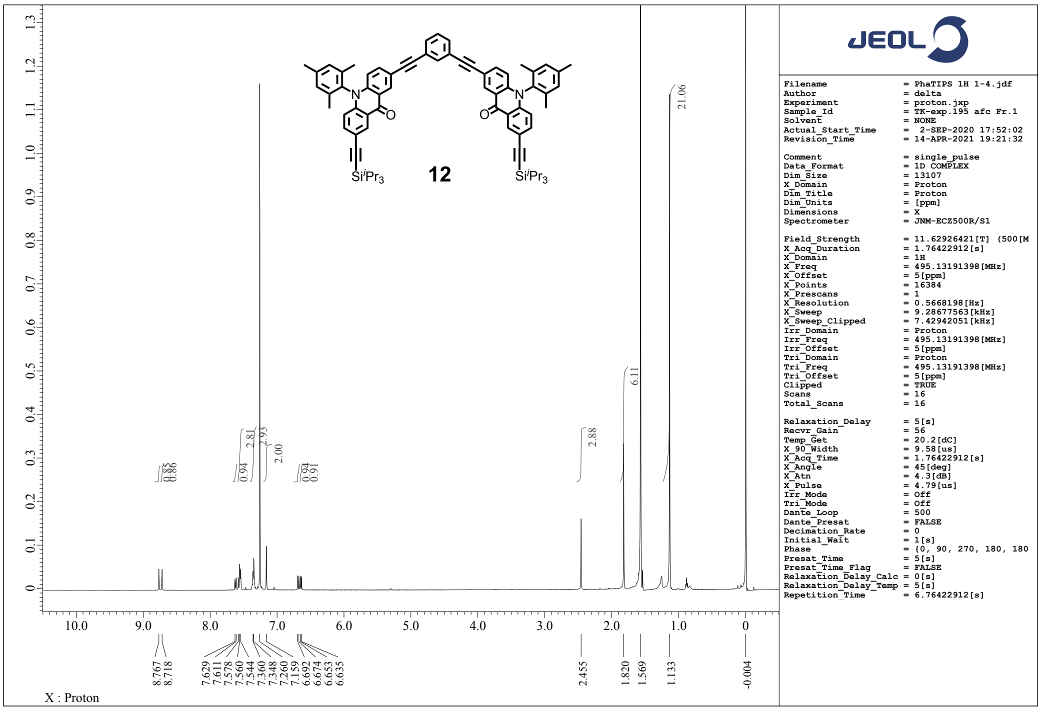

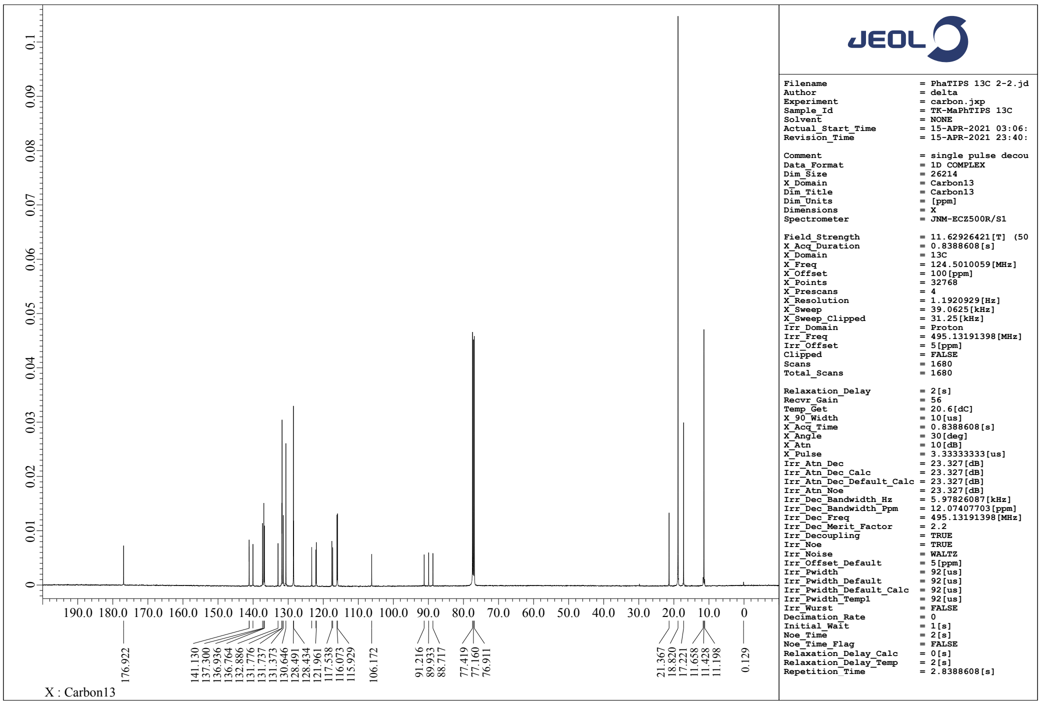

Figure S28. ^{1}H (top, 500 MHz) and ^{13}C NMR spectra (bottom, 125 MHz) of **12** in $CDCl_3$.

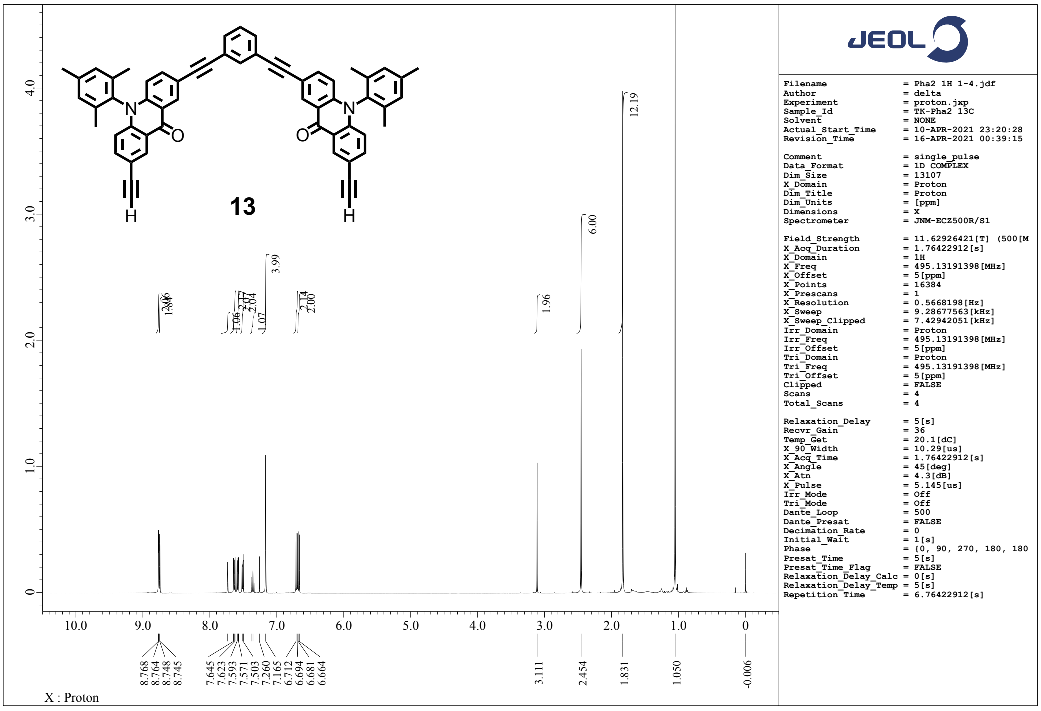

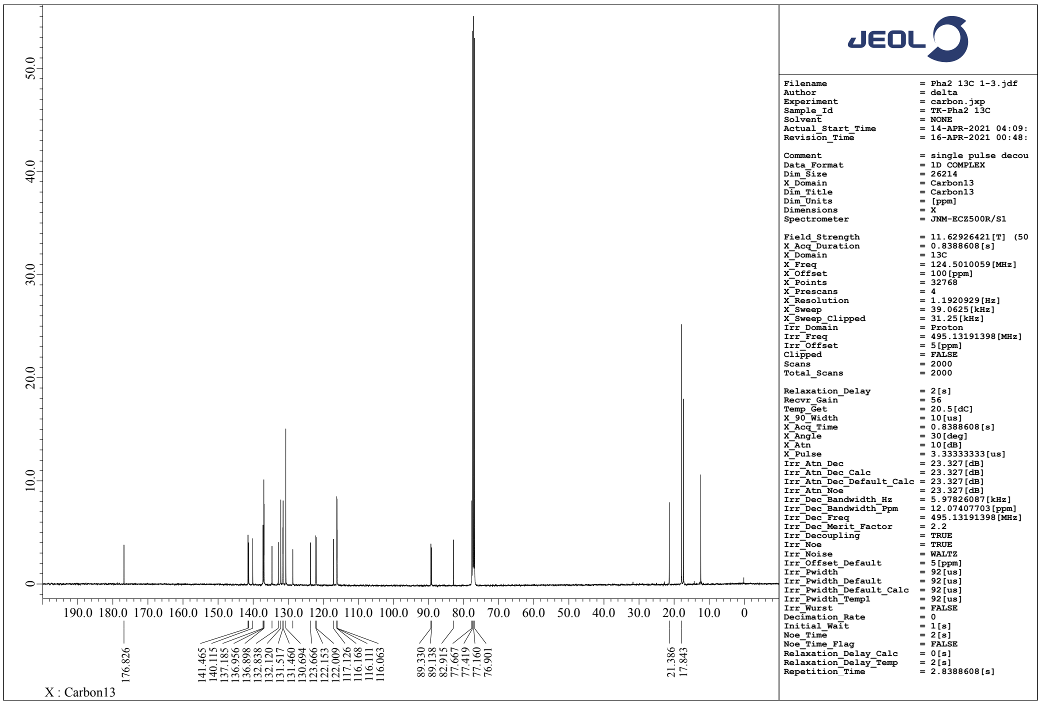

Figure S29. ^{1}H (top, 500 MHz) and ^{13}C NMR spectra (bottom, 125 MHz) of **13** in $CDCl_3$.

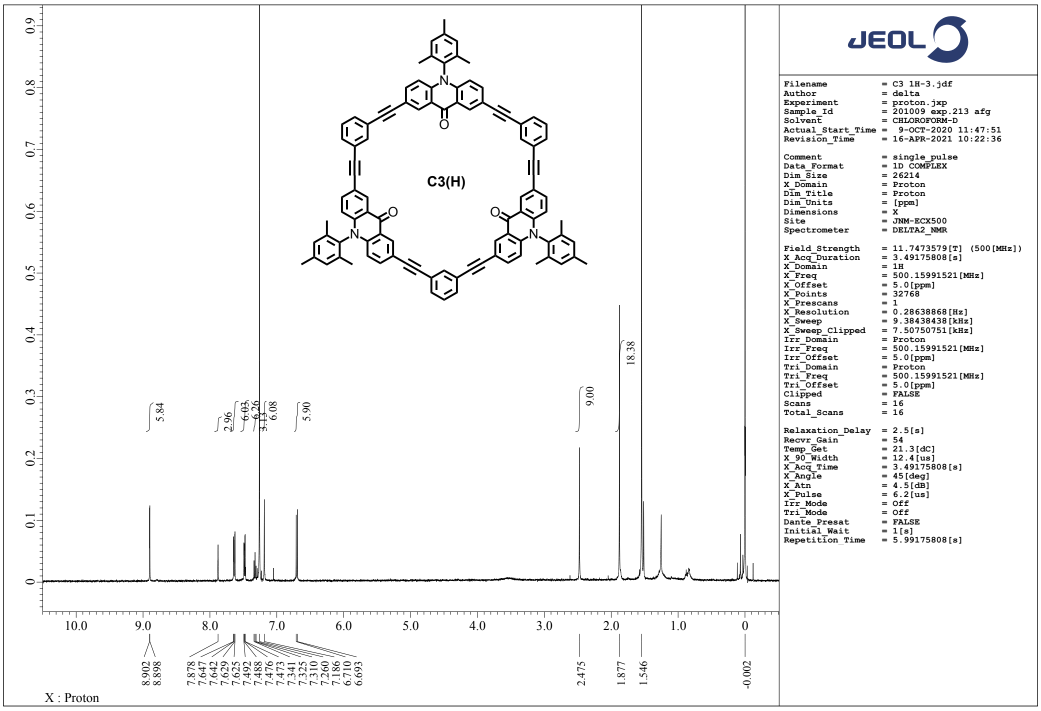

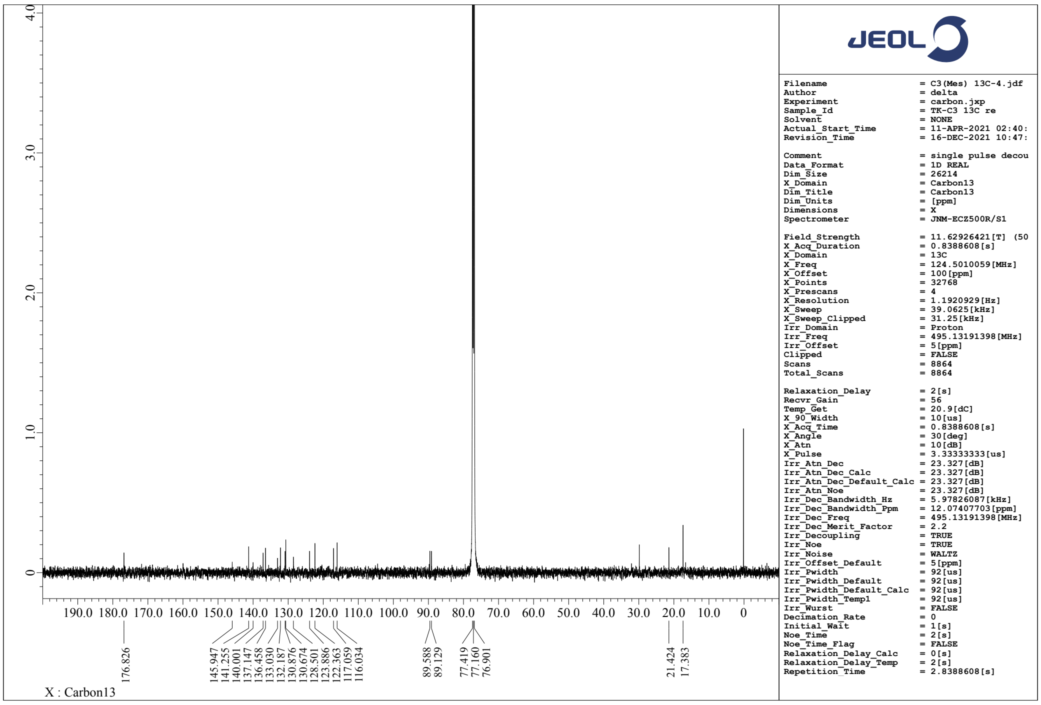

Figure S30. ^{1}H (top, 500 MHz) and ^{13}C NMR spectra (bottom, 125 MHz) of **C3(H)** in $CDCl_3$.

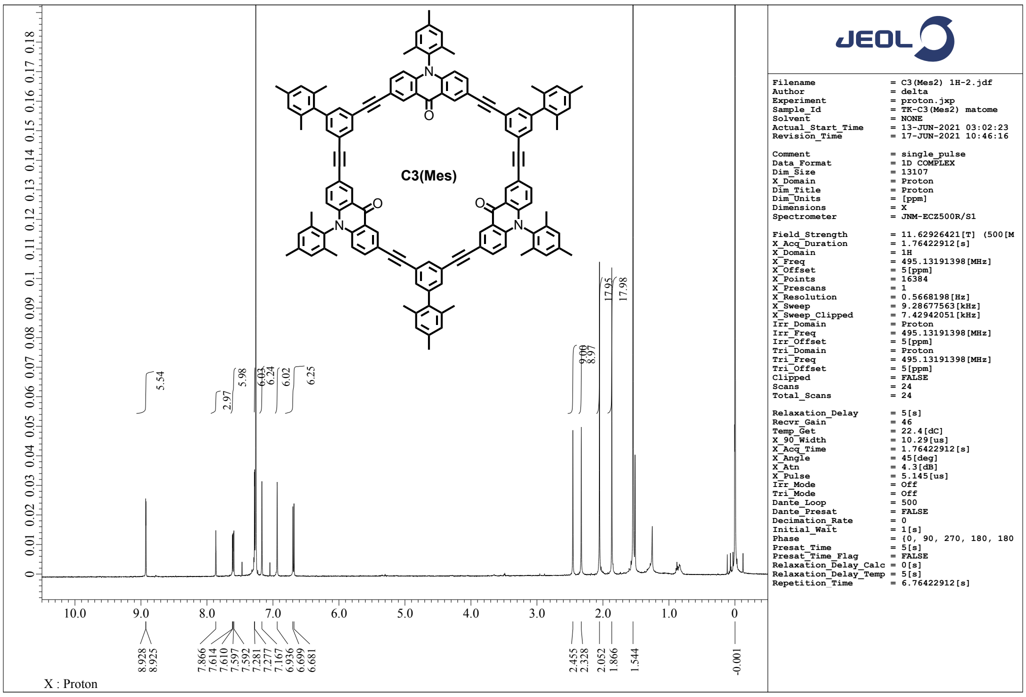

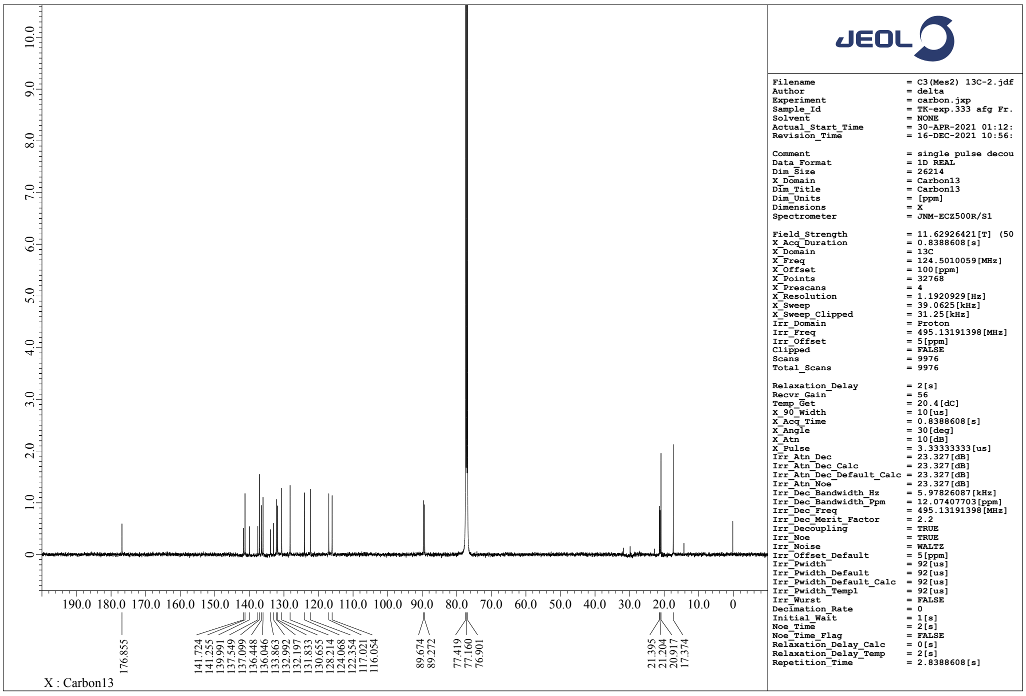

Figure S31. ^{1}H (top, 500 MHz) and ^{13}C NMR spectra (bottom, 125 MHz) of **C3(Mes)** in $CDCl_3$.

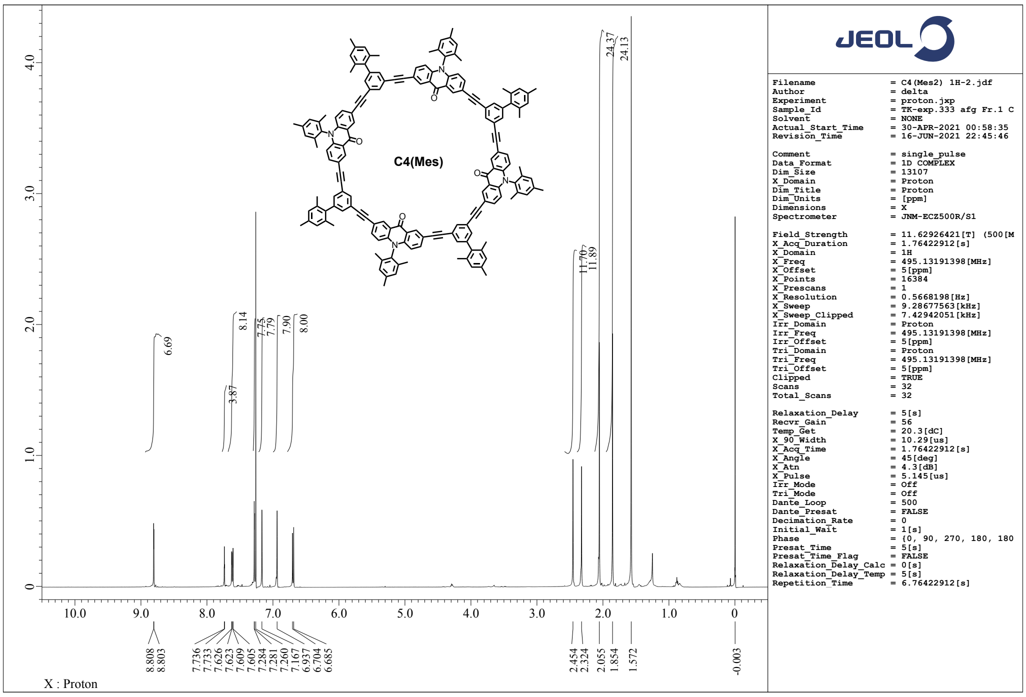

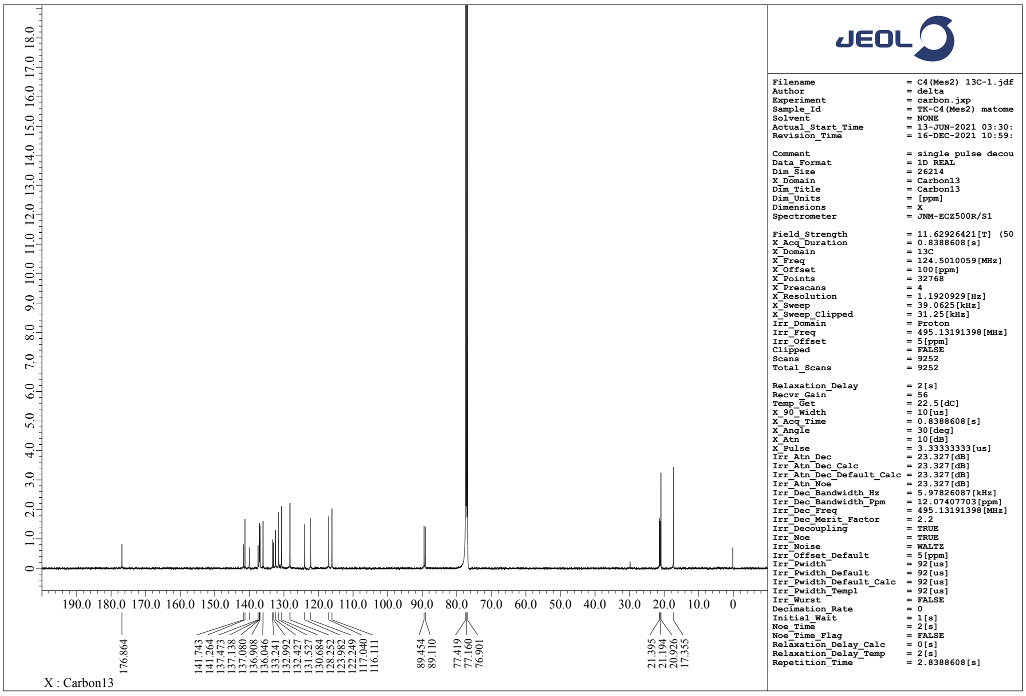

Figure S32. ^{1}H (top, 500 MHz) and ^{13}C NMR spectra (bottom, 125 MHz) of **C4(Mes)** in $CDCl_3$.

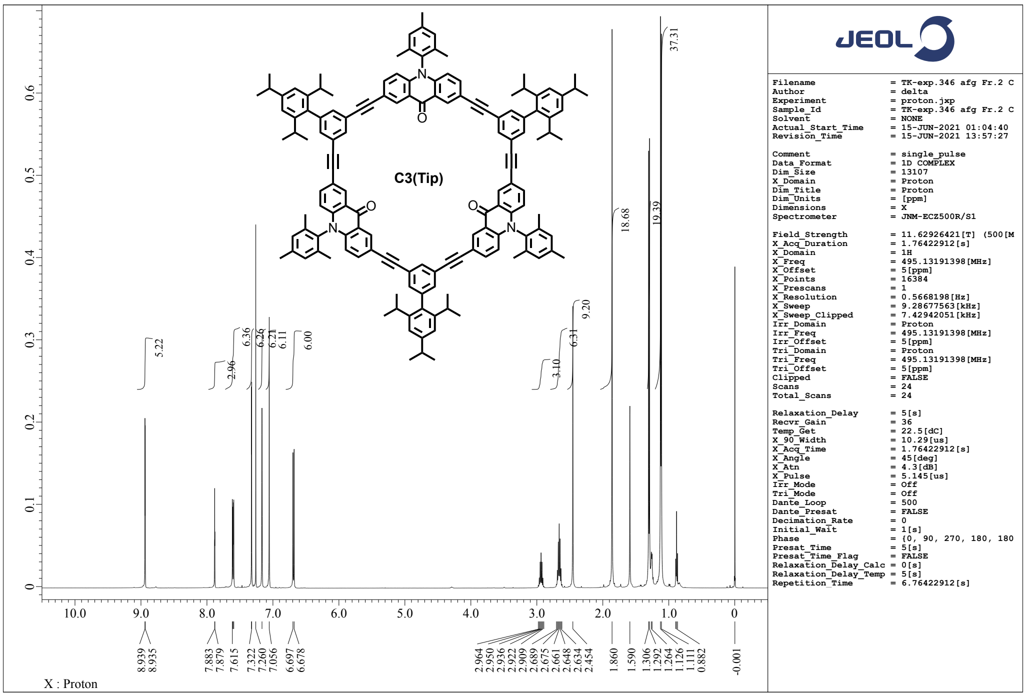

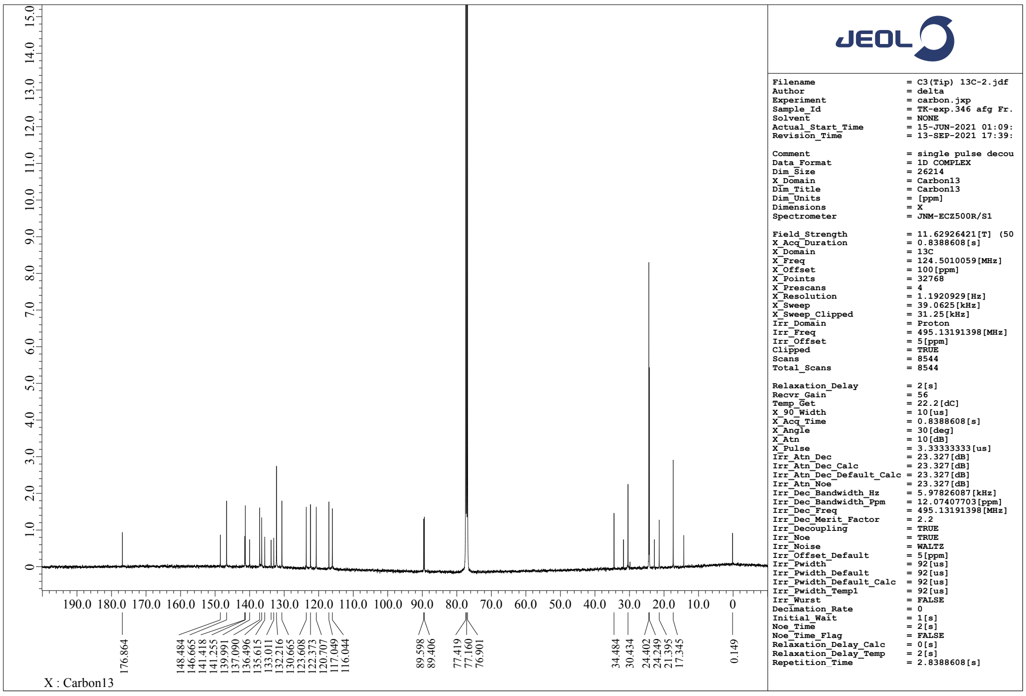

Figure S33. ¹H (top, 500 MHz) and ¹³C NMR spectra (bottom, 125 MHz) of **C3(Tip)** in $CDCl_3$.

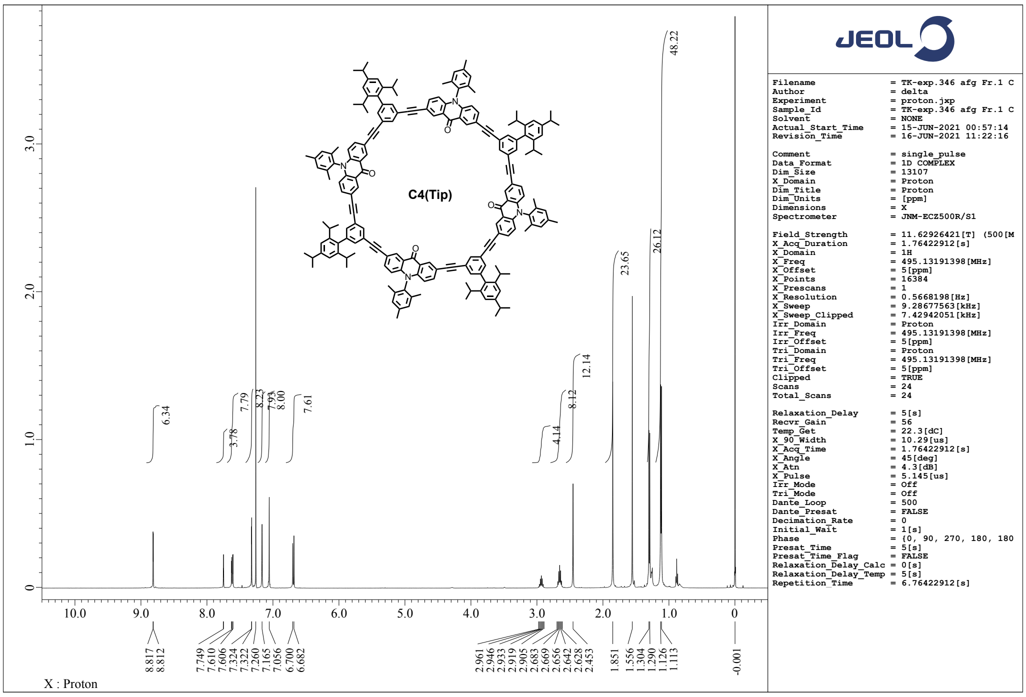

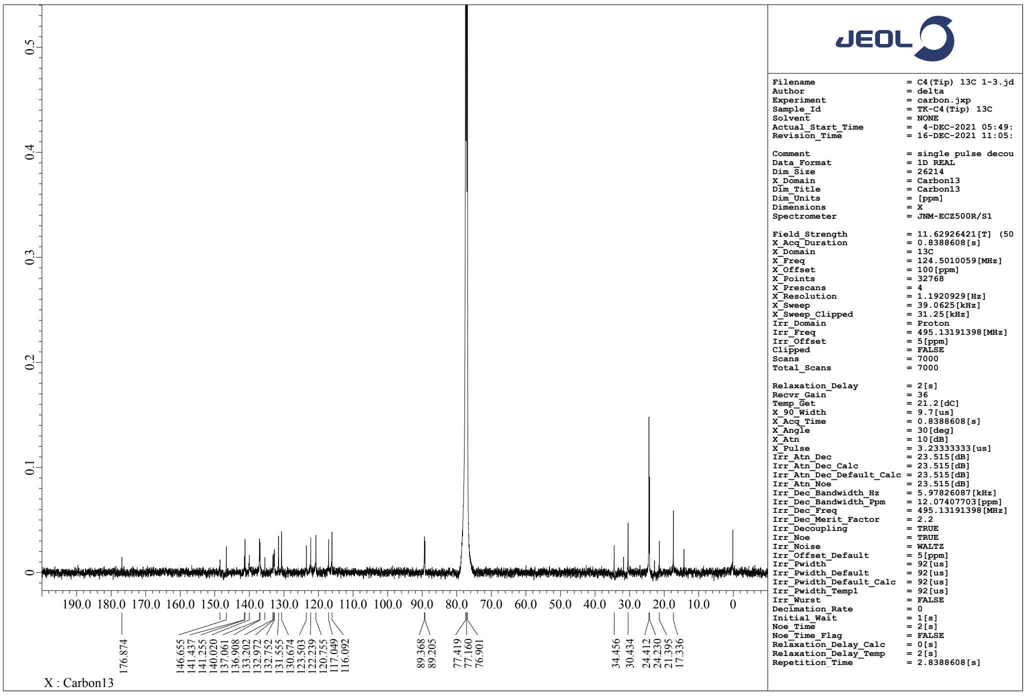

Figure S34. ^{1}H (top, 500 MHz) and ^{13}C NMR spectra (bottom, 125 MHz) of **C4(Tip)** in $CDCl_3$.

References

[S1] T. Komori, E. Tsurumaki, S. Toyota, *Asian J. Org. Chem.* published online, e202200508. (doi.org/10.1002/ajoc.202200508)

[S2] M. Cocchi, D. Virgili, V. Fattori, D. L. Rochester, J. A. G. Williams, *Adv. Funct. Mater.* **2007**, *17*, 285–289.

[S3] H. Chaumeil, S. Signorella, C. L. Drian, *Tetrahedron* **2000**, *56*, 9655–9662.

[S4] C. Reichardt, *Solvents and solvent effects in organic chemistry,* 3rd ed. Wiley-VCH, Weinheim, **2003**, chap. 7.4.

[S5] a) C. Reichardt, *Solvents and solvent effects in organic chemistry,* 3rd ed. Wiley-VCH, Weinheim, **2003**, chap. 2.2; b) V. Gutmann, *Coord. Chem. Rev.* **1976**, *18*, 225–255.

[S6] a) National Institute of Standards and Technology, XIII. Vibration data, Vibrational Frequency scaling factors. https://cccbdb.nist.gov/Intro.asp; b) A. P. Scott, L. Radom, *J. Phys. Chem.* **1996**, *100*, 16502–16513.

[S7] a) G. M. Sheldrick, *Acta Cryst*. **2015**, *A71*, 3–8; b) G. M. Sheldrick, *Acta Cryst*. **2015**, *C71*, 3–8.

[S8] Gaussian 16, Revision C.01, M. J. Frisch, G. W. Trucks, H. B. Schlegel, G. E. Scuseria, M. A. Robb, J. R. Cheeseman, G. Scalmani, V. Barone, G. A. Petersson, H. Nakatsuji, X. Li, M. Caricato, A. V. Marenich, J. Bloino, B. G. Janesko, R. Gomperts, B. Mennucci, H. P. Hratchian, J. V. Ortiz, A. F. Izmaylov, J. L. Sonnenberg, D. Williams-Young, F. Ding, F. Lipparini, F. Egidi, J. Goings, B. Peng, A. Petrone, T. Henderson, D. Ranasinghe, V. G. Zakrzewski, J. Gao, N. Rega, G. Zheng, W. Liang, M. Hada, M. Ehara, K. Toyota, R. Fukuda, J. Hasegawa, M. Ishida, T. Nakajima, Y. Honda, O. Kitao, H. Nakai, T. Vreven, K. Throssell, J. A. Montgomery, Jr., J. E. Peralta, F. Ogliaro, M. J. Bearpark, J. J. Heyd, E. N. Brothers, K. N. Kudin, V. N. Staroverov, T. A. Keith, R. Kobayashi, J. Normand, K. Raghavachari, A. P. Rendell, J. C. Burant, S. S. Iyengar, J. Tomasi, M. Cossi, J. M. Millam, M. Klene, C. Adamo, R. Cammi, J. W. Ochterski, R. L. Martin, K. Morokuma, O. Farkas, J. B. Foresman, and D. J. Fox, Gaussian, Inc., Wallingford CT, **2016**.

[S9] GaussView, Version 6.1.1, R. Dennington, T. A. Keith, J. M. Millam, Semichem Inc., Shawnee Mission, KS, **2016**.

[S10] a) R. A. Boto, F. Peccati, R. Laplaza, C. Quan, A. Carbone, J.-P. Piquemal, Y. Maday, J. Contreras-García, *J. Chem. Theory Comput.* **2020**, *16*, 4150–4158; b) E. R. Johnson, S. Keinan, P. Mori-Sánchez, J. Contreras-García, A J. Cohen, W. Yang, *J. Am. Chem. Soc.* **2010**, *132*, 6498–6506.

[S11] VMD Version 1.9.3: W. Humphrey, A. Dalke, K. Schulten, *J. Molec. Graphics* **1996**, *14*, 33–38.

[S12] P. Thordarson, *Chem. Soc. Rev.* **2010**, *40*, 1305–1323.
